# Prophylactic and Therapeutic Efficacy of Ultrasonicated *Rosmarinus officinalis* Ethanolic Extract and its Chitosan-Loaded Nanoparticles Against *Eimeria tenella* Infected Broiler Chickens

**DOI:** 10.1007/s11686-024-00793-3

**Published:** 2024-03-16

**Authors:** Shaimaa M. Kasem, Nabila M. Mira, Ibrahim B. Helal, Magdy E. Mahfouz

**Affiliations:** 1 Zoology Department, Faculty of Science, Kafrelsheikh University, Kafr ElSheikh, 33516 Egypt; 2 Zoology Department, Faculty of Science, Tanta University, EL Gharbia, 31527 Egypt

**Keywords:** Chitosan nanoparticles, Cytokines, *Eimeria tenella* oocyst, Gene expression, *Rosmarinus officinalis* extract

## Abstract

**Purpose:**

The in vivo efficacy of ultrasonicated *Rosmarinus officinalis* ethanolic extract (UROEE) and its chitosan-loaded nanoparticles (UROEE-CsNPs) was investigated as a dietary prophylactic agent and as a therapeutic treatment against *Eimeria tenella* infected broiler chickens.

**Methods:**

Chickens were infected with 4 × 10^4^ *E. tenella* oocysts at 21 days old for primary infection and with 8 × 10^4^ oocysts at 35 days old for secondary infection. Eleven experimental groups were conducted. Dietary addition of 100 mg/kg UROEE and 20 mg/kg for CsNPs as well as UROEE-CsNPs were included for prophylactic groups from day 1 to 42. The same doses were used for therapeutic treatment groups for 5 constitutive days. Oocyst output in feces was counted. Histopathological and immunohistochemical studies were conducted. Gene expression of pro-inflammatory cytokines as IFN-γ, IL-1β and IL-6 as well as anti-inflammatory cytokines as IL-10 and TGF-β4 was analyzed using semi-quantitative reverse transcriptase-PCR.

**Results:**

The results showed an efficacy of UROEE, CsNPs and UROEE-CsNPs in reduction of oocyst excretion and improving the cecal tissue architecture. CD4^+^ and CD8^+^ T lymphocytes protein expression were reduced. *E. tenella* infection lead to upregulation of pro-inflammatory cytokines as IFN-γ, IL-1β, IL-6 and anti-inflammatory cytokines as TGF-β4 following primary infection, while their expression was downregulated following secondary infection.

**Conclusion:**

The dietary prophylactic additives and therapeutic treatments with UROEE, CsNPs and UROEE-CsNPs could decrease the inflammatory response to *E. tenella* as indicated by oocyst output reduction, histopathological improvements, CD4^+^ and CD8^+^ T cells protein expression reduction as well as reducing mRNA expression levels of the tested cytokines following primary and secondary infections. Consequently, these results will help to develop better-combating strategies for the control and prevention of coccidiosis on poultry farms as a dietary prophylactic agent or as a therapeutic treatment.

**Graphical Abstract:**

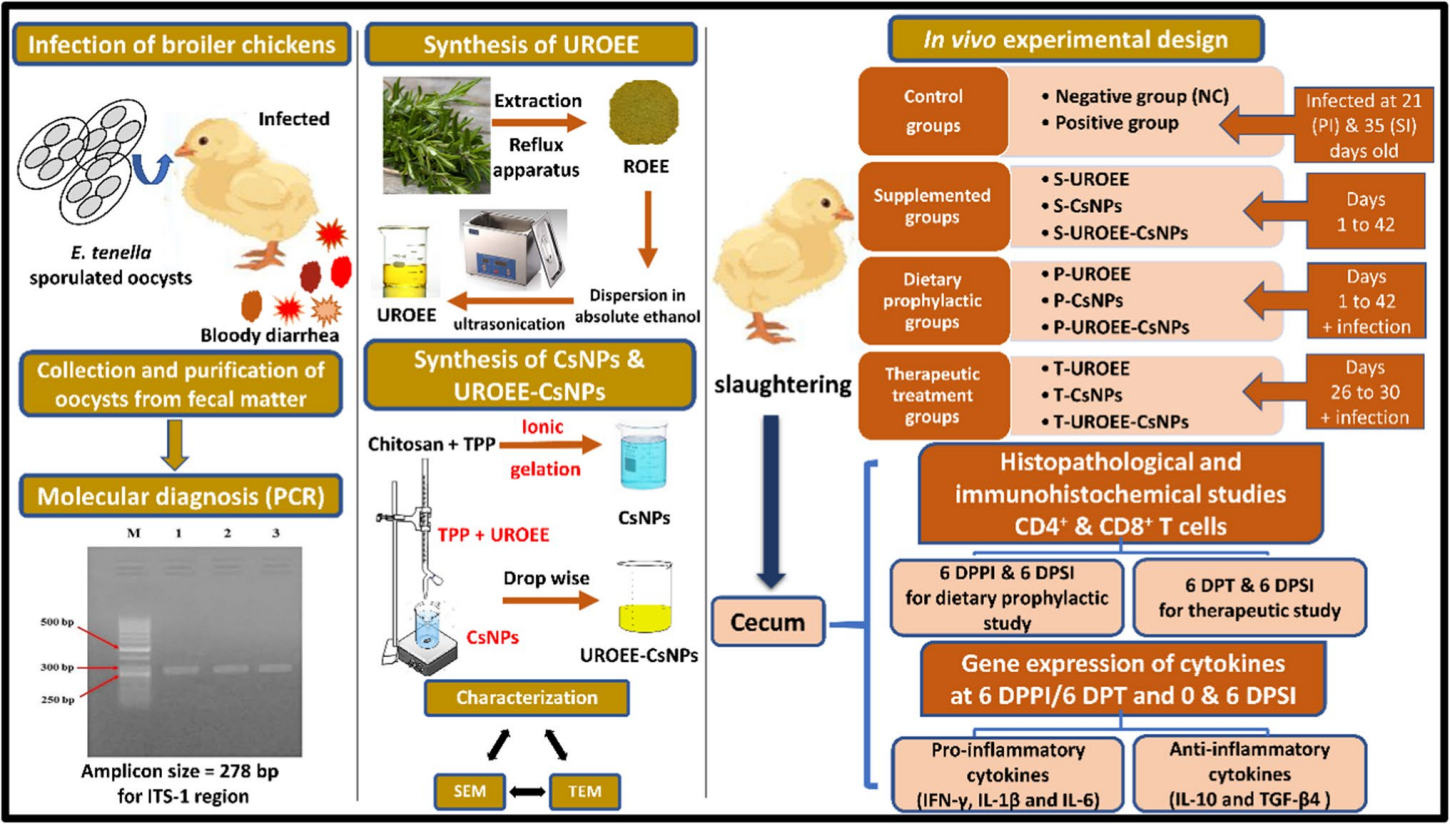

**Supplementary Information:**

The online version contains supplementary material available at 10.1007/s11686-024-00793-3.

## Introduction

Chicken coccidiosis is an avian intestinal disease caused by seven distinct species of Eimeria parasites that damage the host’s digestive system, lead to poor nutrition absorption, reduced growth and often death [1] resulting in enormous economic losses in the poultry industry that is estimated to cost more than 14.5 billion USD annual losses globally [2]. Among the seven *Eimeria* spp. is *E. tenella*; being the most pathogenic species [3] that infects the ceca of chickens [4]. Because the life cycle of *Eimeria* is complex; comprises intracellular, extracellular, asexual and sexual stages, immune responses to *Eimeria* are complex and involve many aspects of nonspecific and specific immunity, the latter involves both humoral and cellular immune mechanisms [5, 6]. However, it appears that humoral immune responses play a minor role in protective immunity against coccidiosis. Instead, cell-mediated immunity constitutes the major host response conferring resistance to parasite infection [7]. Cell-mediated immunity in avian coccidiosis is characterized by antigen-specific or non-specific activation of several immune cells such as T cells, NK cells, and macrophages. The CD4^+^ T helper and CD8^+^ T cytotoxic lymphocytes are the two major T-cell subsets that are involved in anticoccidial immunity [5, 8]. Increased populations of T cells are linked to elevated production of pro-inflammatory cytokines which has an immunoregulatory effect [9]. Cytokines are secreted proteins that regulate the nature of immune responses by affecting growth, differentiation and activation of cells. They are involved in almost all stages of immunity and inflammation, and cytokine production is induced by a variety of stimuli such as viral, bacterial or parasitic infection, cancer, inflammation, or the interaction between T cells and antigens [10].

Coccidiosis remains the most unconquerable disease in poultry fields because of its resistance to climatic change and the ability of *Eimeria* parasites to retain their infectivity for a long time [1]. Chicken coccidiosis can be controlled and treated with polyether ionophore antibiotics, chemically synthesized anticoccidial drugs and vaccines [3]. However, the excessive use of anticoccidial drugs has resulted in the development of resistant strains of *Eimeria* species [11]. Furthermore, the continuous usage of such drugs led to pronounced toxic effects on birds [12] and food residues inducing deleterious effects on human health [13]. Although the use of vaccines is an effective alternative to anticoccidial drugs, its use is limited due to the lengthy manufacturing process, high cost of developing and licencing new vaccines, as well as the risk of pathogen transmission [14]. With an increasing demand for poultry meat, dietary supplements from natural sources could modulate the microbiome, enhance innate immunity and reduce financial losses due to enteric diseases such as coccidiosis [15, 16]. Rosemary (*Rosmarinus officinalis* L.), which belongs to the Lamiaceae family, is an aromatic non-toxic plant that is usually used as a common household culinary spice for flavoring [17]. It has been found to exert various pharmacological activities, particularly antioxidant [18], anti-inflammatory [19] and antiparasitic [20, 21] effects. Furthermore, rosemary extracts are widely used in alternative medicine [22, 23]. Nanotechnology is an exciting research area with numerous applications, particularly in the medicine and poultry industry [24, 25]. Nanomedicine involves the use of nano-structured materials for the diagnosis, prevention, or treatment of diseases [26]. In addition, bioactive nanomaterials can also act as carriers to enhance efficacy and precision by delivering therapeutic or diagnostic agents to cells and tissues [27, 28]. Chitosan is a naturally occurring polymer extracted mainly from crustacean shells and has been used to construct nanoparticles, which are biocompatible, biodegradable, less toxic, easy to prepare and can function as effective drug delivery systems. Furthermore, chitosan is generally recognized as safe (GRAS) by the US Food and Drug Administration (U.S. FDA) [26] plus its antiparasitic [29, 30], anti-inflammatory, immunological and antioxidant effects [31].

A previous study by [21] demonstrated that the loading of ultrasonicated *Rosmarinus officinalis* ethanolic extract (UROEE) on chitosan nanoparticles (CsNPs) had the potential to be used as an in vitro anticoccidial agent against *E. tenella* oocysts of chickens. Therefore, in the present study, the aim of this research is to study the in vivo efficacy of UROEE and its loaded CsNPs against *E. tenella* infected broiler chickens as a dietary prophylactic agent and as a therapeutic treatment using a number of investigations as oocyst output, mortality rate, histopathological and immunohistochemical studies as well as gene expression of pro- and anti- inflammatory cytokines.

## Materials and Methods

### Materials

Low molecular weight chitosan (90–95% deacetylation of about 50 kDa), Tris HCl (MW = 157.60), Tris buffer base (Mw = 121.14 g/mol) and boric acid (Mw = 61.83 g/mol) were obtained from Oxford, Mumbai, India. Agarose was provided by Genetix Biotech, Asia, India. Sodium tripolyphosphate (TPP) (Mw = 367.86 g/mol) was purchased from Sigma-Aldrich. Potassium dichromate (MW = 249.19 g/mol), sodium chloride (MW = 58.44 g/mol), zinc sulfate (MW = 287.56 g/mol) and EDTA disodium salt (MW = 372.23 g/mol) were obtained from Raheja Centre, Mumbai, India. Glacial acetic acid (99.5%) and absolute ethanol (99.9%) were purchased from ADWIC, Egypt.

### Methods

#### *Rosmarinus officinalis* (Rosemary) Extract Preparation

*Rosmarinus officinalis* ethanolic extract (ROEE) was prepared as previously described [21, 32]. The powdered extract yield was kept in amber bottles at 4 °C for the forthcoming purposes.

#### Synthesis of Chitosan Nanoparticles (CsNPs) and Ultrasonicated *Rosmarinus officinalis* Ethanolic Extract-Chitosan Loaded Nanoparticles (UROEE-CsNPs)

ROEE (1 mg/ml absolute ethanol) was sonicated using a 40 kHZ Ultrasonic Water Bath (PT-ZPS-3A, PRISMA TECH, USA) for 15 min forming UROEE. CsNPs were prepared using the ionic gelation method as previously described [33] and UROEE-CsNPs was prepared as previously indicated [21]. Chitosan solution was used at pH 5.

#### Characterization Techniques

The synthesized nanoparticles were characterized using scanning electron microscopy (SEM, JEOL, JSM-IT-100—operated at a voltage of 20 kV) to observe their morphology. The shape and grain size of these nanoparticles were determined using transmission electron microscopy (TEM–JEOL, JEM 2100).

#### Collection and Sporulation of Parasite

*Eimeria tenella* oocysts were obtained from the cecum of naturally infected Ross broiler chickens and stored in 2.5% potassium dichromate solution at 4 °C for further use. The obtained unsporulated oocysts were allowed to sporulate to be infective by incubation at 25–29 °C for 48 h in partially covered Petri dishes to allow the oxygen to be passaged. Humidity was allowed by placing distilled H_2_O in two Petri dishes in the incubator. After oocysts being sporulated, they were counted per gram feces (OPG) [34, 35] using the McMaster counting chamber technique.

#### Propagation of *E. tenella*

A total of ten Ross broiler chickens (14 days old) were infected with 4 × 10^4^ sporulated *E. tenella* oocysts. Beginning from 5 days post infection, fresh fecal matter was collected from different sites of the bedding material, purified with a concentration flotation technique using zinc sulphate saturated solution [36], sporulated and stored in 2.5% potassium dichromate solution at 4 °C for subsequent use.

### Molecular Identification of *E. tenella* Species

#### Extraction of Genomic DNA from *E. tenella* Oocysts

Sporulated oocysts that collected and stored in a 2.5% potassium dichromate solution were used for DNA extraction. Genomic DNA was extracted using QIAamp DNA stool mini kit (Qiagen, Germany) as per the manufacturer’s protocol. The purified sporulated oocysts were washed 3 times through centrifugation in autoclaved phosphate-buffered saline solution at 14.000 rpm (for 5 min, each wash) to remove the potassium dichromate used in preservation. Ten cycles of freezing using liquid nitrogen and thawing in a shaking water bath at 50 °C, were carried out for complete rupturing of oocysts walls without adding sodium hypochlorite or use of glass beads [37]. A drop of the sample was examined using a microscope under a 40 × objective to confirm disruption of the oocyst wall. Following the confirmation of oocysts wall disruption, sporulated oocysts suspension (200 µl) were pipetted into a 2 ml microcentrifuge tube. About 1.4 ml of stool lysis buffer (buffer ASL) were added to remove inhibitory substances from stool samples, vortexed continuously for 1 min and heated for 5 min at 70 °C. The tube was then vortexed for 15 s and centrifuged at 14.000 rpm for 1 min to pellet stool particles. The supernatant (1.2 ml) was transferred by pipetting into a new 2 ml microcentrifuge tube and the pellet was discarded. The subsequent steps were processed as per the QIAamp DNA Stool kit protocol. The DNA was eluted in 50 µl Tris–EDTA (TE, pH 8.0) buffer. DNA concentration was measured at A260 nm, while DNA purity was detected at A_260_/_280_ ratio using a Nanodrop One Microvolume UV–Vis Spectrophotometer (Thermo Scientific™) and kept temporarily at 4 °C.

#### DNA Amplification by Polymerase Chain Reaction (PCR)

Three aliquots of genomic DNA isolated from sporulated oocysts were used for PCR amplification of the internal transcribed spacer-1 (ITS-1) region for *E. tenella*. The PCR species-specific forward primer (5′-AATTTAGTCCATCGCAACCCTTG-3′) and reverse primer (5′-CGAGCGCTCTGCATACGACA-3′) [38, 39] were used to identify *E. tenella*. The primers were synthesized by metabion international AG, (Germany). Following the delivery, the primers were equilibrated at room temperature and dissolved in TE buffer (pH 8.0) to get 100 µM stock. Stock primers were diluted using TE buffer (pH 8.0) to get a working solution of 10 µM and kept at − 20 °C until use. The PCR reaction mixture was carried out in 50 µl volume reaction using 1 µl of DNA template (100 ng/µl), 1 µl of each forward and reverse primers (10 µM), 25 µl of 2X PCR master mix (Bioline, Germany) in a Thermal Cycler (Lab cycler, SENSOQUEST, Germany) and the rest was completed with double distilled H_2_O. The best PCR reaction conditions were optimized at an initial denaturation at 95 °C for 3 min, followed by 35 cycles of denaturation at 95 °C for 30 s, annealing at 58 °C for 30 s, and extension at 72 °C for 45 s, and a final extension step of 72 °C for 10 min. Using the gradient PCR method, three different annealing temperatures (52, 58 and 62 °C) were tested. The results revealed that the optimal annealing temperature was at 58 °C. Samples were kept at 4 °C until analyzed.

#### Gel Electrophoresis

PCR product samples (8.3 µl), mixed with 1.7 µl of 6X DNA loading buffer (Cat. No. B002S, enzynomics, Korea) were separated by running in 1% agarose gel electrophoresis for 35 min at 100 Volts, stained with ethidium bromide (0.5 µg/ml) and a 50 bp ladder (SiZer™-50 plus DNA Marker, Cat. No. 24072; iNtRON Biotechnology, Inc., Korea) was used to confirm the product size. The stained gels were visualized and photographed under a UV Transilluminator (UVP Photo-Doc-It™ Imaging system, VWR International, LLC, Jena).

#### Experimental Design, Diets and Infection

A total of 165 one-day-old male Ross broiler chicks were purchased from a commercial hatchery in Kafr ElSheikh City, Egypt. Upon arrival, the chicks were reared on an open-sided and previously fumigated battery cages to obtain a coccidian-free environment. On day one, the chicks were wing banded, and randomly allocated to 11 equal groups (15 chicks/group). Each group had three replicate pens (5 chicks/pen). Animals were acclimatized and kept in an animal facility room with a regulated temperature between 28 ± 2 °C, humidity (50 ± 5%) and light/dark cycle (18/6 h) till the end of the experiment (Trial period was 42 days).

#### Experimental Infection of Broiler Chickens with *E. tenella* Oocysts

After counting of sporulated *E. tenella* oocysts as previously described, each chick in the infected groups was orally inoculated intra-crop with a dose of 4 × 10^4^ *E. tenella* sporulated oocysts suspended in normal saline solution using an oral gavage at 21 days old to induce a primary infection. Secondary infection was performed following the same procedure as above but orally infected with 8 × 10^4^ sporulated *E. tenella* oocysts suspended in normal saline solution using an oral gavage at 14 days post-primary infection (DPPI) (day 35).

#### Feeding of Experimental Broiler Chickens

All the experimental groups were fed a basal diet without any anticoccidial drugs during the experiment. Food and tap water were provided ad libitum. The feeders and drinkers were washed daily using boiling water to reduce the risk of contamination. These basal diets were formulated for starter (1–14 d), grower (15–28 d) and finisher (29–42 d) growth periods (El Tawheed Co., Kafr ElSheikh, Egypt). Table 1 presents the ingredients and the composition of the basal diet.

**Table 1:** Composition (%) of the basal diet used in the experimental design

Ingredients	Starting diet (1–14 d)	Growing diet (15–28 d)	Finishing diet (29–42 d)
Yellow corn	50.57	55.34	61.25
Soybean meal, 44%	36.38	30.14	25.20
Corn gluten, 60%	4.72	5.50	5.30
Soybean oil	4.11	4.92	4.35
Calcium carbonate	1.66	1.44	1.30
Calcium dibasic phosphate	1.63	1.67	1.80
NaCl	0.43	0.43	0.40
Vitamin and mineral Permix (1826)	0.30	0.30	0.30
L Lysine, Hcl	0.10	0.10	0.10
DL-methionine	0.10	0.16	–
Calculated composition			
Metabolisable Energy, (Kcal/kg)	3030	3150	3130
Crude protein (%)	23.00	21.00	19.00
Crude fat (%)	6.67	7.59	7.17
Crude fibre (%)	3.93	3.58	3.35

The experimental groups were as follows; chickens in

Group 1 (negative control); were fed a basal diet without any additives.

Group 2 (Positive control); were infected with *E. tenella* sporulated oocysts at 21 days old for primary infection and at 14 DPPI (35 days old) for secondary infection.

Groups 3, 4 and 5 (supplemented groups);

Group 3: were administrated with dietary addition of UROEE (100 mg/kg).

Group 4: were administrated with dietary addition of CsNPs (20 mg/kg).

Group 5: were administrated with dietary addition of UROEE-CsNPs (20 mg/kg).

Each group was supplemented with these dietary additives from day 1 to day 42.

Groups 6, 7 and 8 (prophylactic groups);

Group 6: were supplemented with a dietary addition of UROEE (100 mg/kg).

Group 7: were supplemented with dietary addition of CsNPs (20 mg/kg).

Group 8: were supplemented with dietary addition of UROEE-CsNPs (20 mg/kg).

Each group was supplemented with these dietary additives from day 1 to day 42 and infected with *E. tenella* sporulated oocysts at 21 days old for primary infection and at 14 DPPI (35 days old) for secondary infection.

Groups 9, 10 and 11 (therapeutic groups);

were infected with *E. tenella* sporulated oocysts at 21 days old for primary infection and at 14 DPPI (35 days old) for secondary infection and treated with UROEE (100 mg/kg body weight), CsNPs (20 mg/kg body weight) and UROEE-CsNPs (20 mg/kg body weight), respectively after 5 days of primary infections (From the beginning of symptoms) for 5 constitutive days. A treatment period of five days was selected as this was the estimated period of oxidant insult induced by the coccidian parasite [40, 41].

## Measurements

### Oocysts Output

Oocysts output following *E. tenella* infection was used as an assessment for anticoccidial activity and resistance to coccidial infection. Beginning from 4 days after experimental infection with *E. tenella* sporulated oocysts, the fecal matter was examined daily until oocysts were observed. The first appearance of oocysts in any group was recorded as the prepatent period. When oocysts were found and determining the prepatent period, fresh fecal samples were collected every two days and the number of oocysts per gram of feces (OPG) was estimated towards the end of the experiment using the McMaster counting chamber method as previously mentioned.

### Mortality Rate

The mortality rate was determined using the formula:

$$\mathrm{Mortality }\left(\mathrm{\%}\right)= \frac{\mathrm{Number\; of\; dead\; chicks\; in\; the\; group}}{\mathrm{Initial\; total\; number\; of\; birds\; in\; the\; group}}\times 100$$

### Histopathological Studies

To observe the effects induced by UROEE, CsNPs and UROEE-CsNPs, histopathological analysis of the cecum was conducted. At 27 (6 DPPI) and 41 (6 days post secondary infection; DPSI) days old for the prophylactic groups as well as at 31 (6 days post treatment; DPT) and 41 (6 DPSI) days old for the therapeutic groups, about one centimeter from the cecum (Apex; distal end) was collected, washed with phosphate buffer saline (pH 7.4) and immediately fixed in 10% neutral buffered formalin for histopathological studies. The tissue samples were embedded in paraffin and the tissue sections were cut in 5 µm thickness using microtome. All of the sections were stained with hematoxylin and eosin (H& E) [42]. The dried stained cecal sections were examined using a light microscope (LEICA DM650, Germany) connected to a computer system and photographs were taken with a LEICA ICC50 HD photomicroscope camera (Germany), observed for possible histopathological changes and prepared for better illustrations.

### Immunohistochemical Studies

To explore whether the different anticoccidial effects were caused by UROEE, CsNPs and UROEE-CsNPs in the cecum, also immunohistochemistry analysis was used to evaluate the expression of characteristic markers of anti-chicken CD4 and CD8 T lymphocytes. At 27 (6 DPPI) and 41 (6 DPSI) days old for the dietary prophylactic groups as well as at 31 (6 DPT) and 41 (6 DPSI) days old for the therapeutic treatment groups, tissue samples of cecum (Apex; distal end) were taken for immunohistochemical studies. The tissues were washed with normal physiological saline solution and immediately fixed in 10% neutral buffered formalin at room temperature. The tissues were dehydrated within ascending grades of alcohol and then cleared using xylene. Impregnation and embedding of the tissues in hard paraffin blocks was included. The tissue blocks were sectioned using a rotary microtome (Reichert-Jung, 820 H, USA) at 4 µm thickness on glass slides and kept for 15 min in an incubator at 60 °C. The tissue sections on glass slides were deparaffinized by placing in xylol for 15 min twice. Sections were then rehydrated in increasing ethyl alcohol concentrations (100, 100, 95, 80 and 70%) for 5 min each. Endogenous enzymes activity was blocked by placing the sections in 3% hydrogen peroxide (H_2_O_2_) in ethanol at room temperature for 15 min, after which the sections were washed with phosphate-buffered saline (PBS, pH 7.0). Antigen retrieval was conducted by incubating the slides in citrate buffer solution (pH 6.0) for 20 min in the water bath at 98 °C. The tissue sections were then left in room temperature for 30 min and the sections were washed again three times (5 min for each wash) by PBS. Non-specific antigen binding was blocked with blocking buffer (normal goat serum) for 30 min at room temperature in dark humified chambers and the sections were washed again three times (5 min for each wash) by PBS. Primary chicken anti-CD4 protein polyclonal (1: 200, catalog No: bs-0647R-TR, BiOSS ANTIBODIES, US) and anti-CD8 alpha protein polyclonal antibodies (1: 200, catalog No: bs-4791R, BiOSS ANTIBODIES, US) were used to recognize anti-chicken CD4 and CD8 T lymphocytes, respectively. These primary antibodies were spilled over the sections, incubated in a dark humified chamber overnight at 4 °C and washed by PBS three times (5 min for each wash). Then, incubated with UltraVision One HRP Polymer for 15 min. DAB chromogen solution was added to DAB buffer solution in a 1: 1 ratio volume. Each tissue slide was covered with 200 µl of DAB chromogen. The sections were then washed by distilled water three turnover 5 min each. Counterstaining of sections was performed with Mayer’s hematoxylin for four sec. The sections were dehydrated, cleared with xylene, mounted with Canada balsam, covered with coverslips and left to dry. Observation of tissue sections was done using 100 × and 400 × magnification and images were taken with a LEICA light microscope (DM750, Germany) equipped with a LEICA ICC50 HD microscope camera (Germany) using LAS EZ imaging software (version 2.1.0).

### Semi-quantitative Analysis

To evaluate the immunohistochemical reaction, a semi-quantitative analysis of T lymphocytes (CD4^+^ and CD8^+^) was performed. Approximately 100 visual microscopic fields were visualized under a microscope at 100 × and 400 × magnification. Scoring results were considered to be negative (-) in the absence of labeling and positive according to the following scores: mild (+, score 1) for ≤ 25 positive microscopic fields, moderate (++, score 2) for 26–50 positive fields, strong (+++, score 3) for 51–75 positive fields, and very strong (++++, score 4) for > 76 positive fields.

### Gene Expression of Some Immune Genes of Pro-inflammatory and Anti-inflammatory Cytokines

At 27 (6 DPPI), 35 (0 DPSI) and 41 (6 DPSI) days for the prophylactic groups as well as at 31 (6 DPT), 35 (0 DPSI) and 41 (6 DPSI) days for the therapeutic groups, three chickens/group were slaughtered and the ceca from each chicken were removed, washed with sterile ice-cold physiological phosphate buffered saline solution, cut into small pieces (about 30 mg), stored immediately in the RNA stabilizing agent; RNA later (BioFlux, Cat. No. BSC54M1, Hangzhou Bioer Technology Co., Ltd) according to the manufacturer’s instructions at a volume ratio of (1 tissue: 5 RNA later), left overnight at 4 °C, frozen and stored at − 20 °C until use for RNA extraction.

### Total RNA Extraction and cDNA Synthesis

After thawing the tissue preserved in RNA later, 30 mg was used to extract total RNA using a BioFlux RNA extraction kit (Simply P Total RNA Extraction kit, Cat. No. BSC52M2, Hangzhou Bioer Technology Co., Ltd) following the manufacturer’s instructions included in the kit. Purified RNA was eluted in 50 µl elution buffer. RNA concentrations were quantified by measuring at A260, while RNA purity was detected at A_260_/_280_ ratio using a Nanodrop One microvolume UV–Vis Spectrophotometer (Quawell, Q9000, USA) and stored at − 80 °C until use. For cDNA synthesis, RNA was reverse transcribed using EasyScript® first strand cDNA synthesis SuperMix kit (Cat. No. AE301, TransGen Biotech Co., Ltd) according to manufacturer’s recommendations and stored at − 20 °C.

### Amplification of cDNA of Some Chickens' Immune Genes by Semi-quantitative Reverse Transcriptase-PCR (RT-PCR)

The mRNA gene sequences of a target organism (*Gallus gallus*) were downloaded in FASTA format from the National Center for Biotechnology Information (NCBI) database (https://www.ncbi.nlm.nih.gov/genbank/) by using the respective accession number as a search query (Table 2). A theoretical test was carried out computer-aided (in silico) to confirm the matching of the selected primers and the expected product size. According to the mRNA immune gene sequences of pro-inflammatory cytokines as interferon-gamma (IFN-γ), interleukin-1beta (IL-1β) and interleukin-6 (IL-6) and anti-inflammatory cytokines as interleukin-10 (IL-10) and transforming growth factor-beta4 (TGF-β4) as well as glyceraldehyde-3-phosphate dehydrogenase (GAPDH) that used as the reference gene, all the primers were synthesized by Metabion International AG, Germany. Primers for GAPDH, IFN-γ, IL-1β, IL-6, IL-10 and TGF-β4 are listed in Table 2. The primers were equilibrated at room temperature, dissolved in TE buffer (pH 8.0) to get 100 µM stock. Stock primers were diluted using TE buffer (pH 8.0) to get a working solution of 10 µM and kept at − 20 °C until use.

**Table 2:** Primers sequences used for the gene encoding mRNAs of some selected proteins using semi-quantitative RT-PCR

RNA target	Type	Primer sequences (5′→3′)	Expected PCR product (bp)	Annealing temperature	Accession no	References
GAPDH	Forward	5ʹ-GGTGGTGCTAAGCGTGTTAT-3ʹ	264	58 °C	K01458	[43, 79, 87, 88]
	Reverse	5ʹ-ACCTCTGTCATCTCTCCACA-3ʹ				
IFN-γ	Forward	5ʹ-AGCTGACGGTGGACCTATTATT-3ʹ	259	58 °C	NM_205149	[43, 79, 87, 88]
	Reverse	5ʹ-GGCTTTGCGCTGGATTC-3ʹ				
IL-1β	Forward	5ʹ-TGGGCATCAAGGGCTACA-3ʹ	244	56 °C	Y15006	[43, 79, 87]
	Reverse	5ʹ-TCGGGTTGGTTGGTGATG-3ʹ				
IL-6	Forward	5ʹ-CAAGGTGACGGAGGAGGAC-3ʹ	254	57 °C	AJ309540	[43, 79, 87, 88]
	Reverse	5ʹ-TGGCGAGGAGGGATTTCT-3ʹ				
IL-10	Forward	5ʹ-CGGGAGCTGAGGGTGAA-3ʹ	272	57 °C	AJ621254	[79, 87]
	Reverse	5ʹ-GTGAAGAAGCGGTGACAGC-3ʹ				
TGF-β4	Forward	5ʹ-CGGGACGGATGAGAAGAAC-3ʹ	258	57 °C	M31160	[43, 43, 79, 87]
	Reverse	5ʹ-CGGCCCACGTAGTAAATGAT-3ʹ				

*GAPDH* glyceraldehyde-3-phosphate dehydrogenase, *IFN-γ* interferon gamma, *IL-1β* interleukin 1 beta, *IL-6* interleukin 6, *IL-10* interleukin 10, *TGF-β4* transforming growth factor beta 4, *bp* base pair

Semi-quantitative RT-PCR was performed using the conventional 2X PCR Master Mix (2X TOPsimple™ DyeMix-*n*Taq, Cat. No. 170523, enzynomics, Korea). The PCR reaction mixture was carried out in 20 µl volume reaction using a cDNA template (500 ng), 2 µl of each forward and reverse primers (10 µM), 10 µl of 2X PCR master mix and if needed, RNase-free water was added to complete the total volume to 20 µl in a Techne TC-3000X PCR Thermal cycler. The reaction was subjected to an initial denaturation at 95 °C for 3 min, followed by 32 cycles of denaturation at 94 °C for 30 s, annealing (according to each specific primer in Table 2) for 30 s, and extension at 72 °C for 1 min, and a final extension step of 72 °C for 5 min. Samples were then kept at – 20 °C until analyzed.

### Agarose Gel Electrophoresis

PCR product samples (5 µl) were directly separated by running in 1% agarose gel electrophoresis for 35 min at 100 Volts. The gels were visualized and photographed under a UV Transilluminator (UVP Photo-Doc-It™ Imaging system, VWR International, LLC, Jena). The gel images were saved (in tiff. format) on a computer for digital image analysis.

### Calculation of Relative mRNA Expression

A semiquantitative analysis of the band intensities was performed using ImageJ software (Java 1.83.0) as described before [44]. GAPDH was used as a reference gene. The intensities of the bands of the target genes of interest were normalized against that of GAPDH and relative gene expression was estimated as fold change.

### Statistical Analysis

Data were analyzed using Statistical Package for Social Science (SPSS, version 20). One-way analysis of variance (ANOVA) was used. Tukey HSD test was applied to determine the statistical differences between means. For values not normally distributed, the non-parametric analysis of the Mann–Whitney *U* test was employed. The results are presented as (mean values ± standard deviation) and considered statistically significant when probability values (*P* values) were less than 0.05 (*P* < 0.05).

## Results

### Nanoparticles Characterization Results

**Fig. 1:**
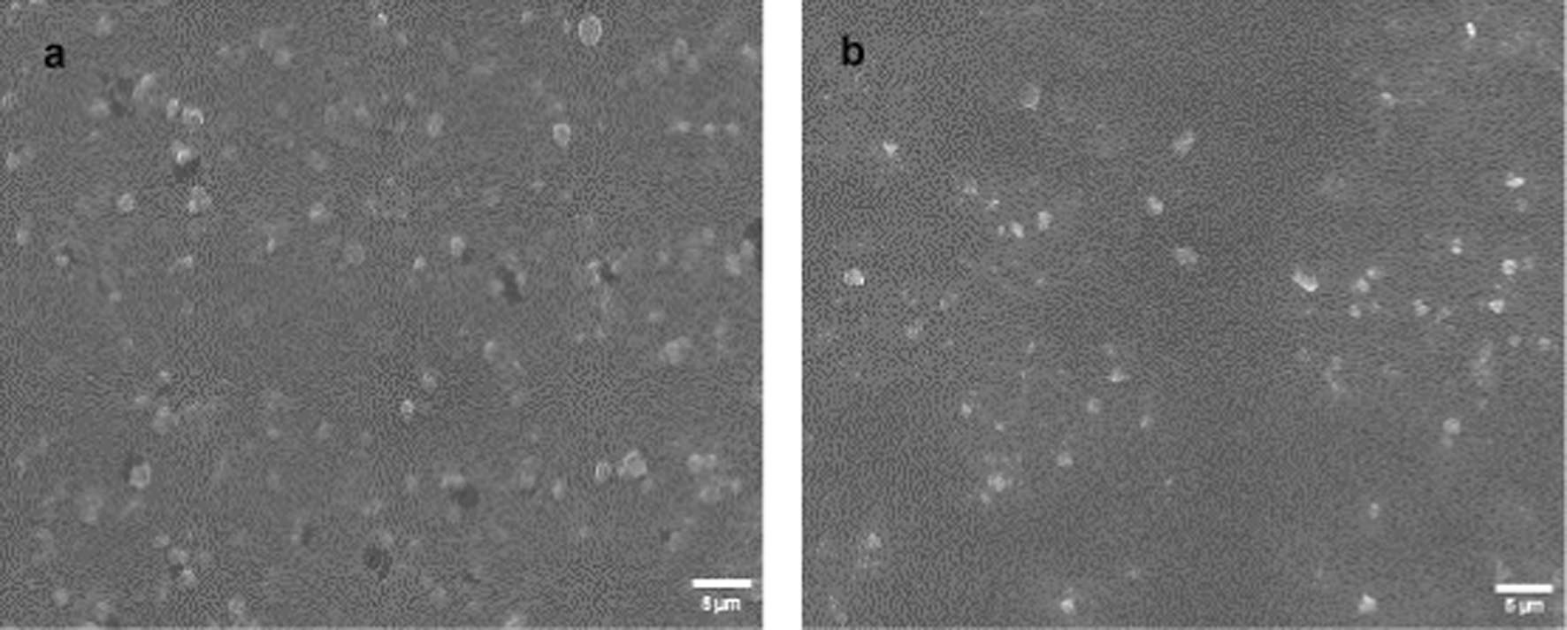
SEM micrographs of **a** CsNPs. **b** UROEE-CsNPs. CsNPs; chitosan nanoparticles. UROEE-CsNPs; ultrasonicated *Rosmarinus officinalis* ethanolic extract-chitosan loaded nanoparticles. Scale bar = 5 µm

**Fig. 2:**
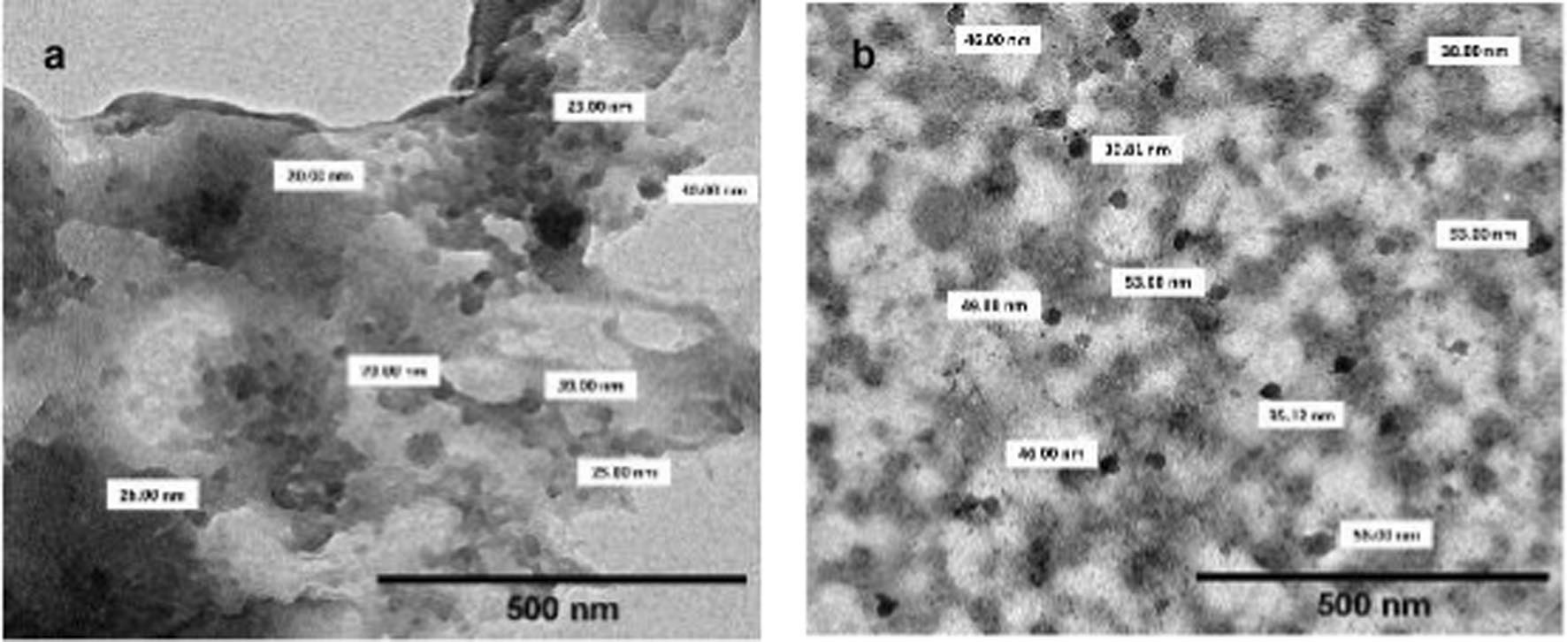
TEM micrographs of **a** CsNPs. **b** UROEE-CsNPs. CsNPs; chitosan nanoparticles. UROEE-CsNPs; ultrasonicated *Rosmarinus officinalis* ethanolic extract-chitosan loaded nanoparticles. Scale bar = 500 nm

SEM micrographs indicated that CsNPs and UROEE-CsNPs had a regular spherical shape (Fig. 1). TEM images confirmed their spherical morphology and showed that CsNPs size was 28.70 ± 3.95 nm, while UROEE-CsNPs size exhibited 48.80 ± 6.84 nm (Fig. 2).

### Molecular Diagnosis Results of *E. tenella* Species

**Fig. 3:**
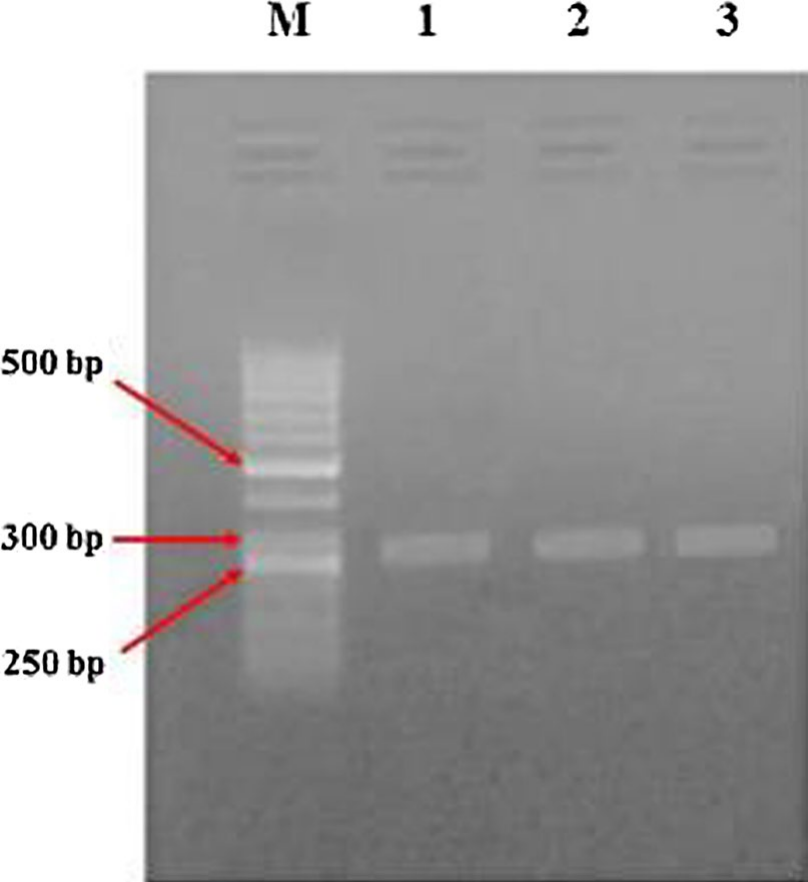
Agarose gel electrophoresis of PCR products for *E. tenella* diagnosis. Lane 1, 2, 3: Representative amplified PCR products using species-specific primers targeting ITS-1 region of *E. tenella*. ITS-1: internal transcribed spacer-1, Lane M: marker, 50 bp ladder

The results of PCR amplification of three samples of genomic DNA extracted from stored sporulated oocysts in potassium dichromate solution that collected from different regions of the chicken’s bedding material revealed the appearance of amplified fragments at 278 bp (Fig. 3) using species-specific primers targeting ITS-1 region of *E. tenella* on 1% agarose gel.

### Clinical Signs

In this study, no coccidian clinical symptoms were observed in chickens of the NC group, where the chickens remained healthy and showed normal appetite throughout the experimental period. However, chickens in the infected positive control group exhibited the typical symptoms of coccidiosis including depression, ruffled feathers, reduction of food intake, weight loss, emaciation, decreased activity and bloody diarrhea, accompanied by progressive weakness.

### Oocysts Per Gram (OPG) Shedding and Mortality Rate

**Fig. 4:**
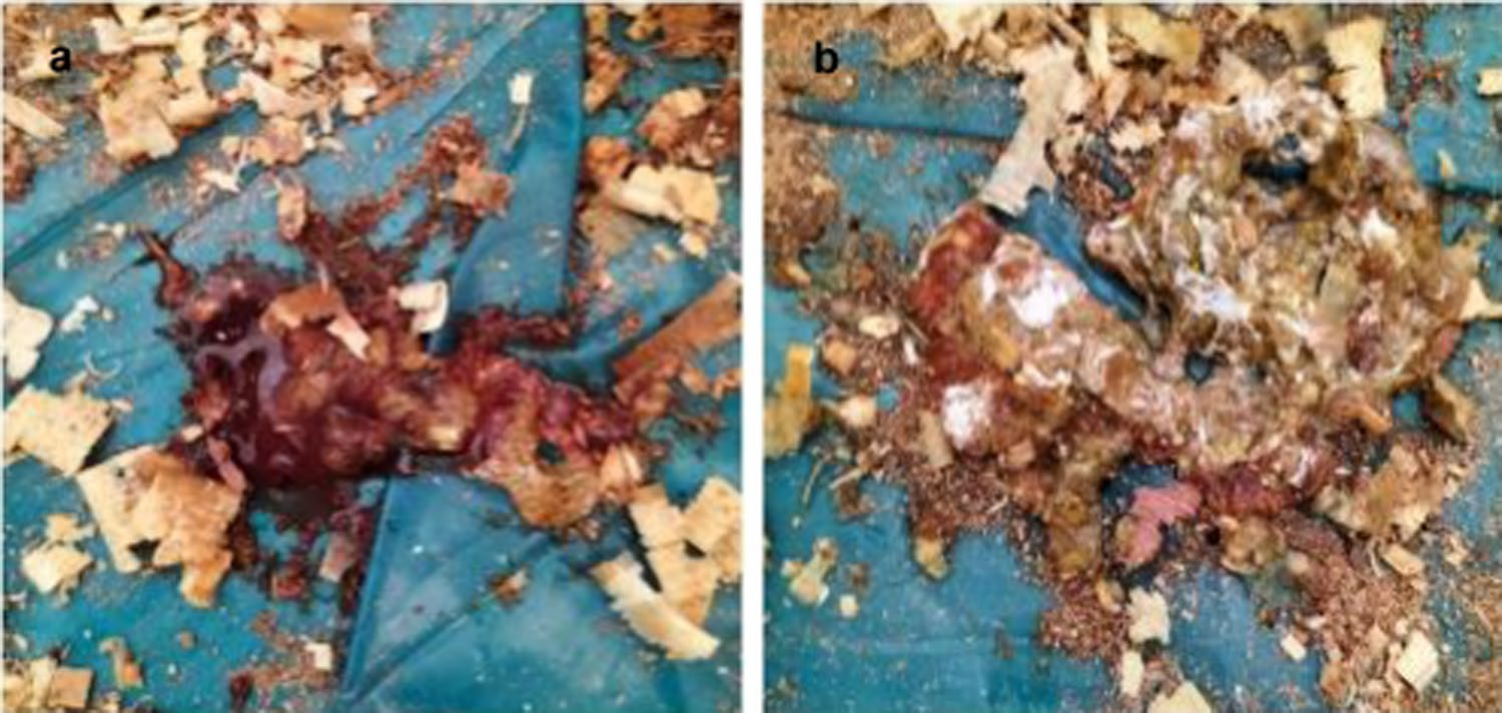
A comparison between the degree of bloody diarrhea in **a** infected, therapeutic treatment groups and **b** dietary prophylactic groups

**Fig. 5:**
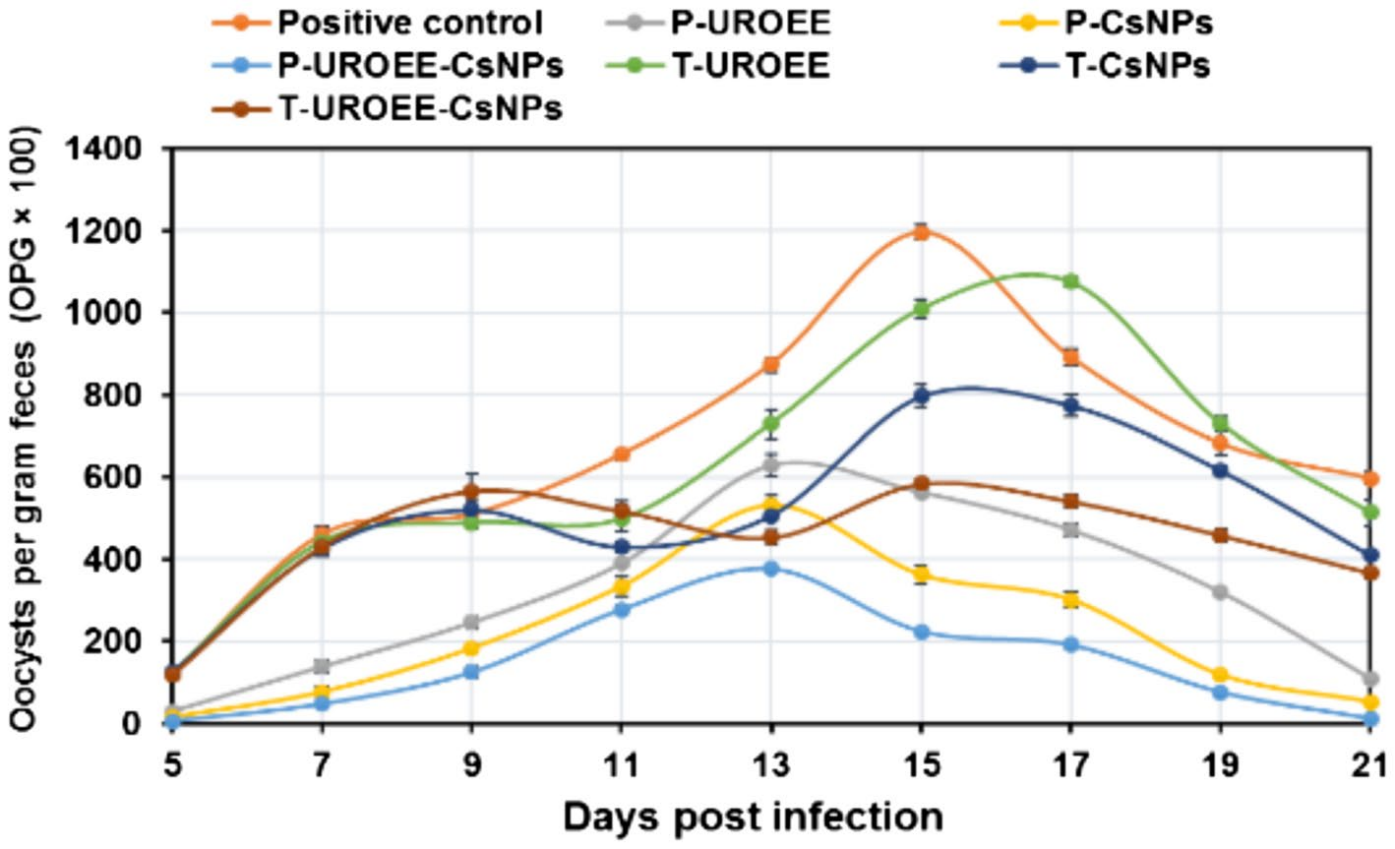
Comparison between the prophylactic and therapeutic effect of ultrasonicated *Rosmarinus officinalis* ethanolic extract and its chitosan-loaded nanoparticles on litter oocysts excretion of infected broiler chickens with *E. tenella* (Data are means ± standard deviation). P-UROEE; dietary prophylactic group with ultrasonicated *Rosmarinus officinalis* ethanolic extract at 100 mg/kg diet, P-CsNPs; dietary prophylactic group with chitosan nanoparticles at 20 mg/kg diet, P-UROEE-CsNPs; dietary prophylactic group with ultrasonicated *Rosmarinus officinalis* ethanolic extract-chitosan loaded nanoparticles at 20 mg/kg diet, T-UROEE; therapeutic treatment group with ultrasonicated *Rosmarinus officinalis* ethanolic extract at 100 mg/kg B.W., T-CsNPs; therapeutic treatment group with chitosan nanoparticles at 20 mg/kg B.W., T-UROEE-CsNPs; therapeutic treatment group with ultrasonicated *Rosmarinus officinalis* ethanolic extract- chitosan loaded nanoparticles at 20 mg/kg B.W

Five DPPI, extensive bloody diarrheal feces were observed in the positive control, T-UROEE, T-CsNPs and T-UROEE-CsNPs groups (Fig. 4a). Whereas, mild bloody diarrhea was seen in P-UROEE, P-CsNPs and P-UROEE-CsNPs groups (Fig. 4b). The prophylactic dietary addition of P-UROEE, P-CsNPs and P-UROEE-CsNPs demonstrated significant (*P* < 0.05) decreases in OPG output from day 26 (5 DPPI) till day 42 (21 DPPI) in comparison to the positive control group indicating that P-UROEE-CsNPs group exhibited the lowest number of OPG output. However, there are no significant differences between the therapeutic groups (T-UROEE, T-CsNPs) and positive control group till day 30 (9 DPPI), while T-UROEE-CsNPs revealed significant (*P* < 0.05) decreases in OPG output related to the positive control group from day 30 (9 DPPI) till day 42 (21 DPPI). In addition, the significant (*P* < 0.05) differences between the three therapeutic groups (T-UROEE, T-CsNPs and T-UROEE-CsNPs groups) were observed from day 36 (15 DPPI). However, there was an observed significant (*P* < 0.05) difference in OPG output between the prophylactic and therapeutic groups (Table 3, Fig. 5). In addition, as presented in Table 5, no deaths were recorded in either the NC or the dietary prophylactic groups (P-UROEE, P-CsNPs and P-UROEE-CsNPs). However, the mortality rate was recorded as 20% in the positive control group within 5–7 days of infection. Moreover, the mortality rate was 15%, 5% and 10% in T-UROEE, T-CsNPs and T-UROEE-CsNPs groups, respectively.

**Table 3:** Comparison between the prophylactic and therapeutic effect of ultrasonicated *Rosmarinus officinalis* ethanolic extract and its chitosan-loaded nanoparticles on litter oocysts excretion of broiler chickens infected with *E. tenella*

Oocysts excretion (×10^2^/g excreta)								
Age (days)	NC	Positive control	Prophylactic groups			Therapeutic groups		
			P-UROEE	P-CsNPs	P-UROEE-CsNPs	T-UROEE	T-CsNPs	T-UROEE-CsNPs
25 (4 DPPI)	0.00	0.00	0.00	0.00	0.00	0.00	0.00	0.00
26 (5 DPPI)		119.67 ± 3.51^•^	29.00 ± 2.00^•*^	15.67 ± 1.16^•*^	5.67 ± 1.16^•*^	122.67 ± 9.07^•^	124.00 ± 11.53^•^	118.00 ± 6.00^•^
28 (7DPPI)		460.33 ± 20.53^•^	138.00 ± 15.10^•*^	76.67 ± 10.79^•*^	47.33 ± 4.16^•*^	443.00 ± 36.37^•^	426.67 ± 12.26^•^	431.67 ± 19.14^•^
30 (9 DPPI)		510.00 ± 20.00^•^	245.33 ± 14.50^•*^	183.33 ± 20.2^•*^	124.33 ± 13.65^•*^	489.67 ± 13.87^•^	520.67 ± 20.65^•^	567.33 ± 40.82^•*^
32 (11 DPPI)		655.67 ± 11.68^•^	389.67 ± 9.50^•*^	334.00 ± 24.52^•*^	277.33 ± 8.74^•*^	499.00 ± 31.76^•*^	430.33 ± 10.60^•*^	518.33 ± 24.03^•*^
34 (13 DPPI)		874.67 ± 18.34^•^	629.00 ± 27.18^•*^	532.00 ± 24.98^•*^	376.67 ± 6.81^•*^	730.33 ± 35.16^•*^	507.00 ± 8.89^•*^	453.67 ± 17.47^•*^
36 (15 DPPI)		1195.33 ± 19.14^•^	563.33 ± 7.64^•*^	362.67 ± 23.0^•*^	224.33 ± 7.57^•*^	1010.67 ± 22.50^•*^	796.67 ± 26.54^•*^	584.67 ± 11.59^•*^
38 (17 DPPI)		891.67 ± 19.14^•^	471.67 ± 15.37^•*^	302.00 ± 19.92^•*^	191.33 ± 7.51^•*^	1075.33 ± 10.60^•*^	775.00 ± 27.50^•*^	542.33 ± 16.04^•*^
40 (19 DPPI)		682.00 ± 28.51^•^	320.33 ± 6.43^•*^	119.00 ± 7.21^•*^	76.33 ± 9.29^•*^	730.30 ± 18.45^•*^	617.33 ± 10.69^•*^	459.33 ± 16.50^•*^
42 (21 DPPI)		597.33 ± 15.57^•^	109.00 ± 9.00^•*^	53.00 ± 4.00^•*^	12.00 ± 2.65^•*^	514.33 ± 30.89^•*^	410.00 ± 8.66^•*^	367.33 ± 13.61^•*^
Mortality rate (%)	0	25	0	0	0	18.75	6.25	12.5

NC; negative control group, P-UROEE; dietary prophylactic group with ultrasonicated *Rosmarinus officinalis* ethanolic extract at 100 mg/kg diet, P-CsNPs; dietary prophylactic group with chitosan nanoparticles at 20 mg/kg diet, P-UROEE-CsNPs; dietary prophylactic group with ultrasonicated *Rosmarinus officinalis* ethanolic extract- chitosan loaded nanoparticles at 20 mg/kg diet, T-UROEE; therapeutic treatment group with ultrasonicated *Rosmarinus officinalis* ethanolic extract at 100 mg/kg B.W., T-CsNPs; therapeutic treatment group with chitosan nanoparticles at 20 mg/kg B.W., T-UROEE-CsNPs; therapeutic treatment group with ultrasonicated *Rosmarinus officinalis* ethanolic extract- chitosan loaded nanoparticles at 20 mg/kg B.W. Data are means ± standard deviation

*Significant (*P* < 0.05), when compared to positive control group

^•^Significant (*P* < 0.05), when compared to negative control group

### Histopathological Findings

**Fig. 6:**
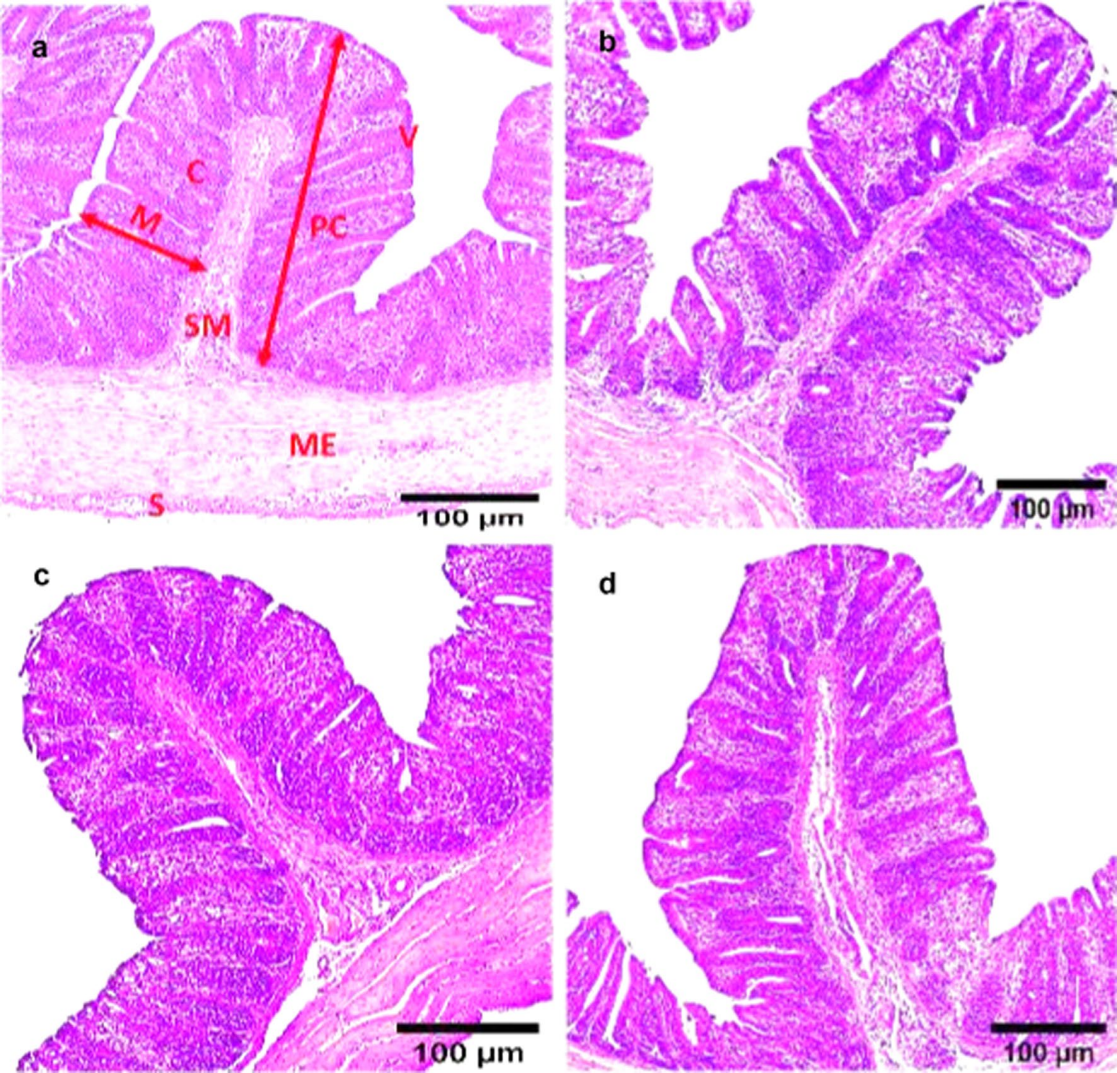
Photomicrographs of cecum of broiler chickens fed basal diet supplemented with ultrasonicated *Rosmarinus officinalis* ethanolic extract and its chitosan-loaded nanoparticles at 27 days old. **a** Negative control group showing normal cecal architecture with four cecal layers: the mucosa (M), Submucosa (SM), Muscularis externa (ME) and Serosa (S). Broad intact circular folds (plicae circulares: PC) with great number of crypts (C) and villi (V) were seen. Dietary additive groups with** b** ultrasonicated *Rosmarinus officinalis* ethanolic extract at 100 mg/kg, **c** chitosan nanoparticles at 20 mg/ kg, and **d** ultrasonicated *Rosmarinus officinalis* ethanolic extract**-**chitosan loaded nanoparticles at 20 mg/kg; showing normal cecal architecture with normal mucosal epithelium, H&E stain, Scale bar = 100 µm

**Fig. 7:**
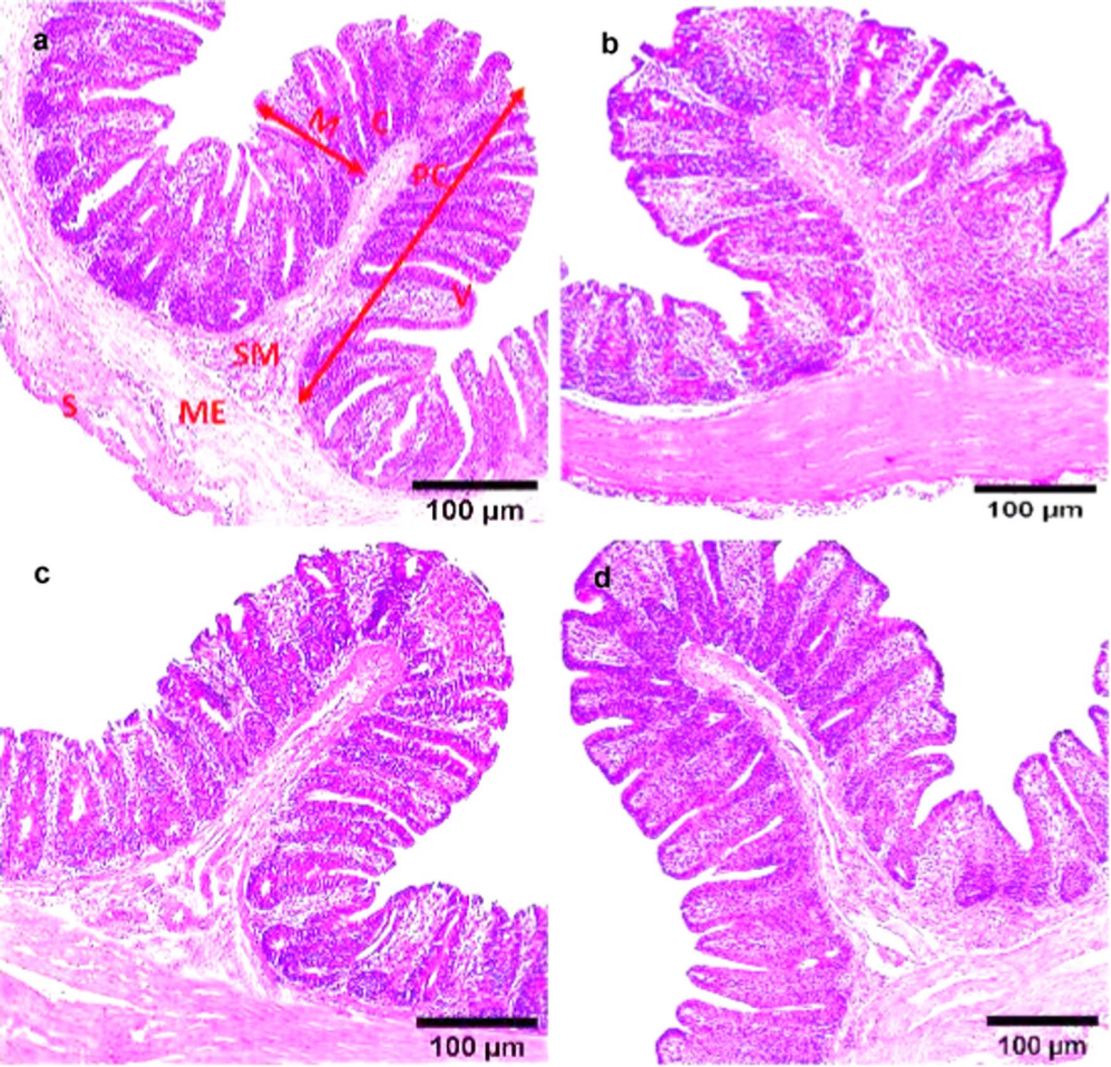
Photomicrographs of cecum of broiler chickens fed basal diet supplemented with ultrasonicated *Rosmarinus officinalis* ethanolic extract and its chitosan-loaded nanoparticles at 41 days old. **a** Negative control group showing normal cecal architecture with four cecal layers: the mucosa (M), Submucosa (SM), Muscularis externa (ME) and Serosa (S). Broad intact circular folds (plicae circulares: PC) with great number of crypts (C) and villi (V) were seen. Dietary additive group with** b** ultrasonicated *Rosmarinus officinalis* ethanolic extract at 100 mg/kg, **c** chitosan nanoparticles at 20 mg/ kg, and **d** ultrasonicated *Rosmarinus officinalis* ethanolic extract**-**chitosan loaded nanoparticles at 20 mg/kg; showing normal cecal architecture with normal mucosal epithelium, H&E stain, Scale bar = 100 µm

Microscopically, the cecal tissue of non-infected, non-treated broiler chickens (NC group) appeared with normal histological structure that consists of four histological layers; mucosa, submucosa, muscularis externa and serosa. However, Broad intact plicae circulares (folds) along the inner surface of the cecal mucosa with several villi were observed. The villi were integrity and arranged regularly. Villus covering epithelium contains simple columnar cells and long, simple, non-branched tubular glands called Lieberkühn glands or crypts at 27 and 41 days old (Figs. 6a, 7a). The cecal tissues of dietary supplemented groups; S-UROEE, S-CsNPs and S-UROEE-CsNPs appeared with normal histopathological features as intact plicae circulares that extended along the inner surface into the cecal lumen with normal mucosa as evidenced by regular cecal glands with its normal small basophilic nuclei and normal villi at 27 days old (Fig. 6b–d) and 41 days old (Fig. 7b–d).

**Fig. 8:**
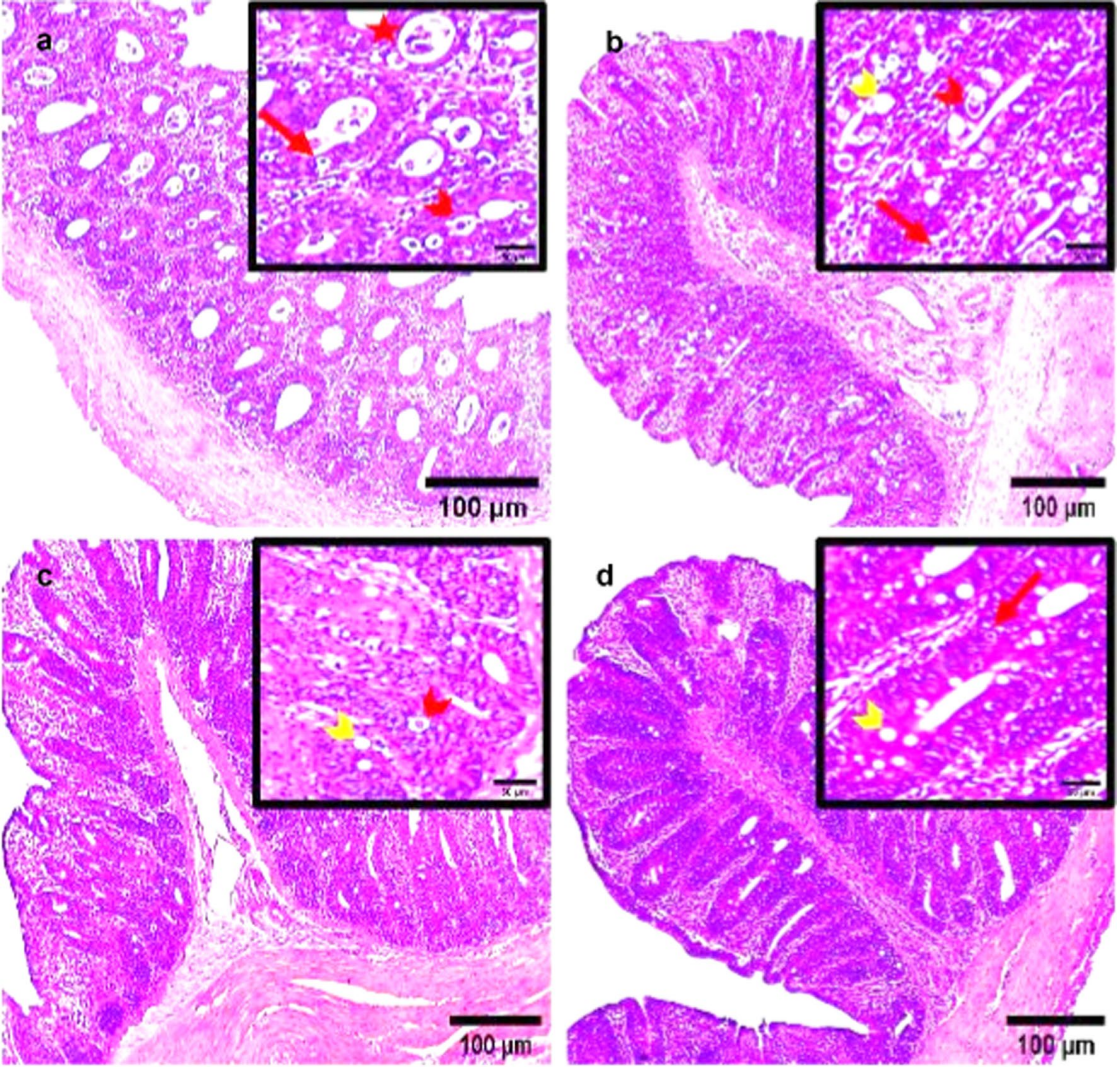
Effect of dietary prophylactic additives of ultrasonicated *Rosmarinus officinalis* ethanolic extract and its chitosan-loaded nanoparticles on histopathological changes of cecum of broiler chickens infected with *E. tenella* at 6 days post primary infection (27 days old). **a** Infected group with *E. tenella* showing marked villus atrophy and inflamed damaged cecal lining, desquamation of superficial epithelium, inflammatory cell infiltration with observed heavy infection with different parasitic developmental stages including oocysts (red arrow), schizonts (arrowhead) and merozoites (star). **b** Dietary prophylactic group with ultrasonicated *Rosmarinus officinalis* ethanolic extract at 100 mg/kg diet showing normality in cecal architecture with moderate amount of oocysts (red arrow), schizonts (red arrowhead) with abnormal vacuolated schizonts (yellow arrowhead) **c** Dietary prophylactic group with chitosan nanoparticles at 20 mg/kg diet showing normality in cecal architecture with mild amount of schizonts (red arrowhead) and vacuolated schizonts (yellow arrowhead) also seen **d** Dietary prophylactic group with ultrasonicated *Rosmarinus officinalis* ethanolic extract**-**chitosan loaded nanoparticles at 20 mg/kg diet showing normality in cecal architecture with few oocysts (red arrow) and large amount of vacuolated schizonts (yellow arrowhead), H&E stain, Scale bar = 100 µm (color figure online)

**Fig. 9:**
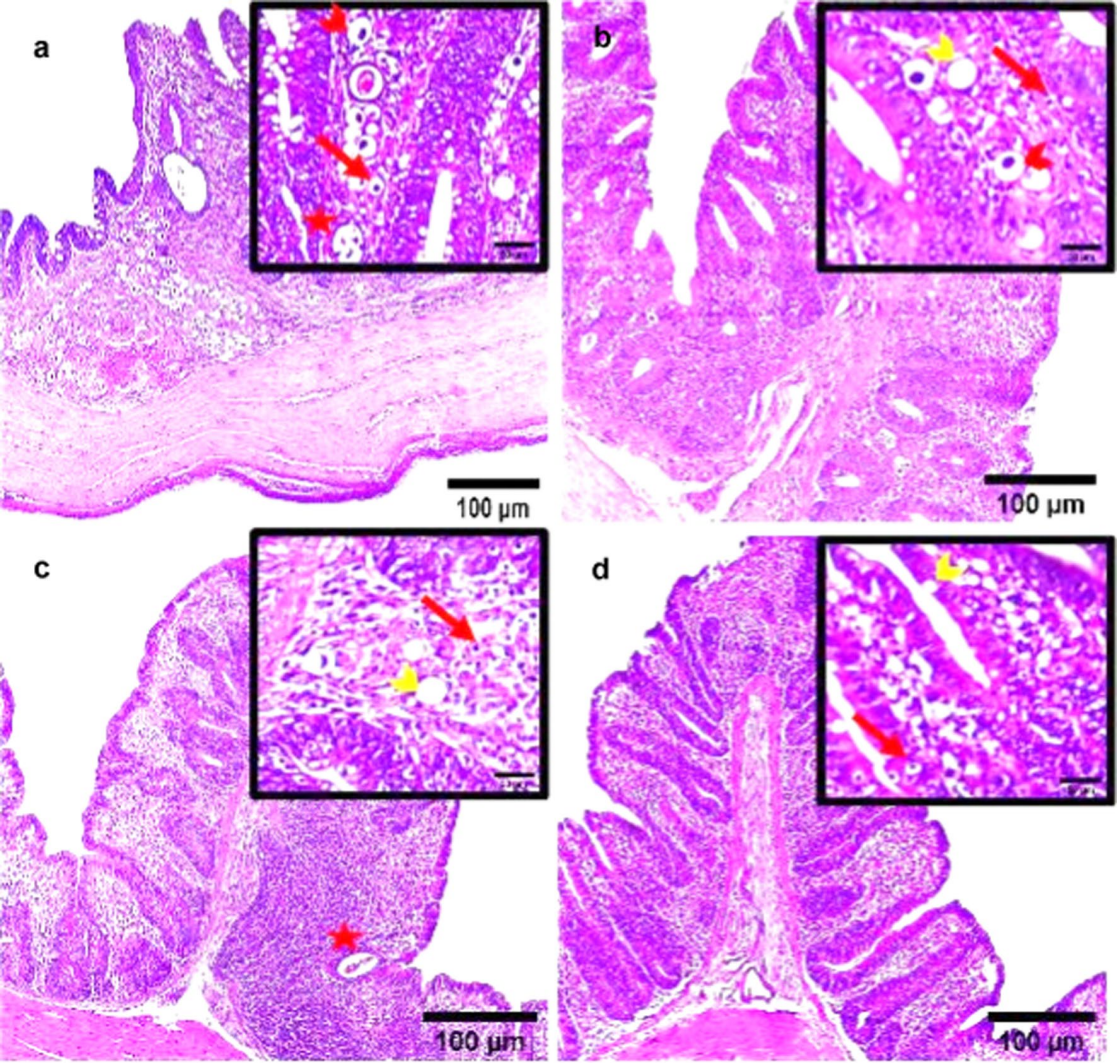
Effect of dietary prophylactic additives of ultrasonicated *Rosmarinus officinalis* ethanolic extract and its chitosan-loaded nanoparticles on histopathological changes of cecum of broiler chickens infected with *E. tenella* at 6 days post-secondary infection (41 days old). **a** Infected group with *E. tenella* showing obvious damaged cecal lining with villus atrophy, sloughing off superficial epithelial cells of folds and marked infiltration of the mucosa with inflammatory cells and different endogenous developmental parasitic stages as schizonts (arrowhead), merozoites (star) and oocysts (red arrow). **b** Dietary prophylactic group with ultrasonicated *Rosmarinus officinalis* ethanolic extract at 100 mg/kg diet showing semi-normality in cecal architecture with mild number of endogenous parasitic stages including oocysts (red arrow), schizonts (red arrowhead) and vacuolated schizonts (yellow arrowhead). **c** Dietary prophylactic group with chitosan nanoparticles at 20 mg/kg diet showing normality in cecal architecture with low amount of oocysts (red arrow), vacuolated schizonts (yellow arrowhead), merozoites (star) and aggregated lymphocytes were observed **d** Dietary prophylactic group with ultrasonicated *Rosmarinus officinalis* ethanolic extract**-**chitosan loaded nanoparticles at 20 mg/kg diet showing normality in cecal structure with rare observed oocysts (red arrow) and vacuolated schizonts (yellow arrowhead), H&E stain, Scale bar = 100 µm (color figure online)

**Fig. 10:**
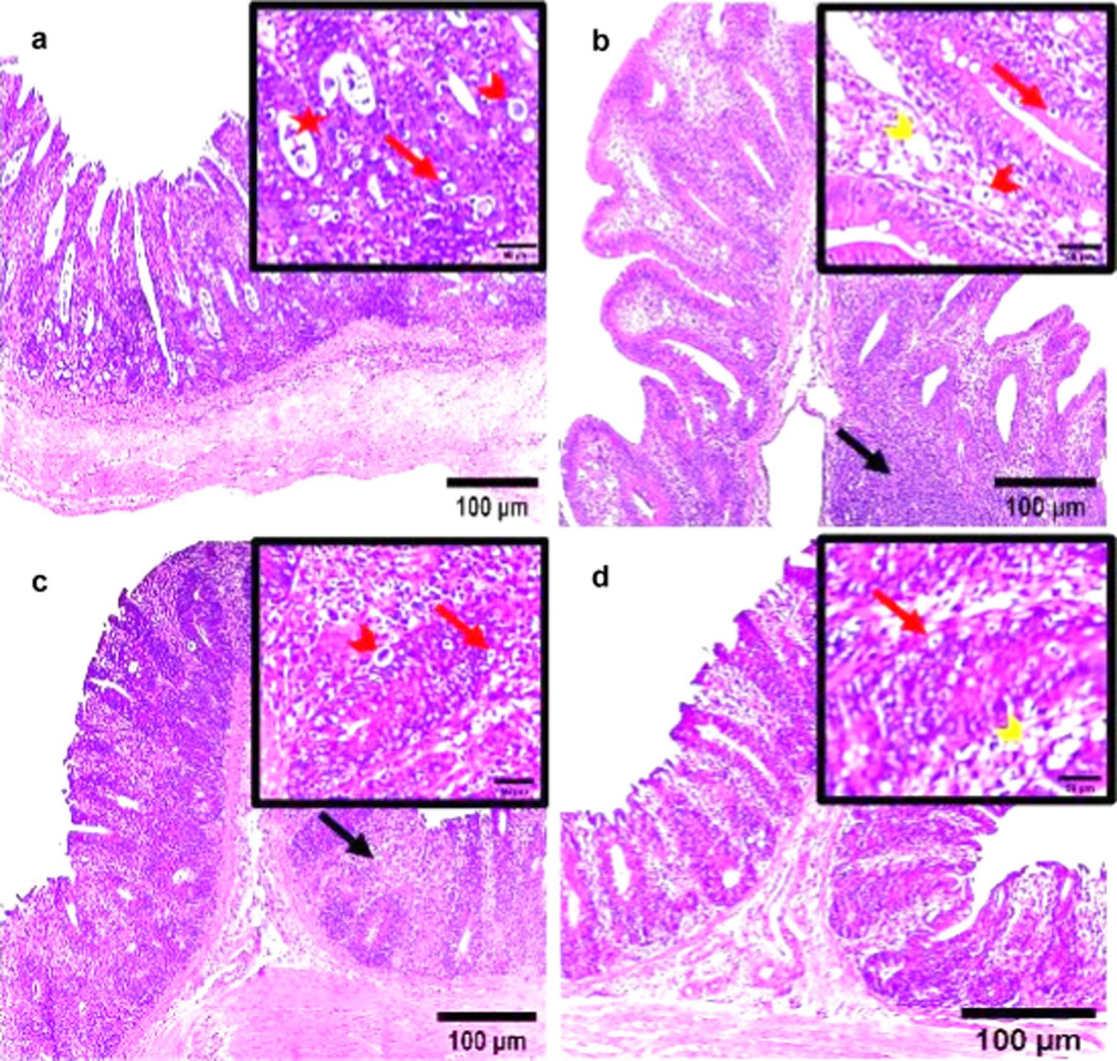
Effect of therapeutic treatment with ultrasonicated *Rosmarinus officinalis* ethanolic extract and its chitosan-loaded nanoparticles on histopathological changes of cecum of broiler chickens infected with *E. tenella* at 6 days post treatment (31 days old). **a** Infected group with *E. tenella* showing irregular villus pattern, marked villus atrophy and damaged cecal lining with observed heavy infection with various parasitic endogenous developmental stages including oocysts (red arrow), schizonts (arrowhead) and merozoites (star). **b** Therapeutic treatment group with ultrasonicated *Rosmarinus officinalis* ethanolic extract at 100 mg/kg B.W. showing semi-normality in cecal architecture with large amount of oocysts (red arrow), schizonts (red arrowhead), vacuolated schizonts (yellow arrowhead) and aggregated lymphocytes (black arrow) were seen **c** Therapeutic treatment group with chitosan nanoparticles at 20 mg/kg B.W. showing semi-normality in cecal architecture with clusters of oocysts (red arrow), few schizonts (red arrowhead) and aggregated lymphocytes (black arrow) were observed **d** Therapeutic treatment group with ultrasonicated *Rosmarinus officinalis* ethanolic extract**-**chitosan loaded nanoparticles at 20 mg/kg B.W. showing normality in cecal architecture with few oocysts (red arrow) and clusters of vacuolated schizonts (yellow arrowhead), H&E stain, Scale bar = 100 µm (color figure online)

**Fig. 11:**
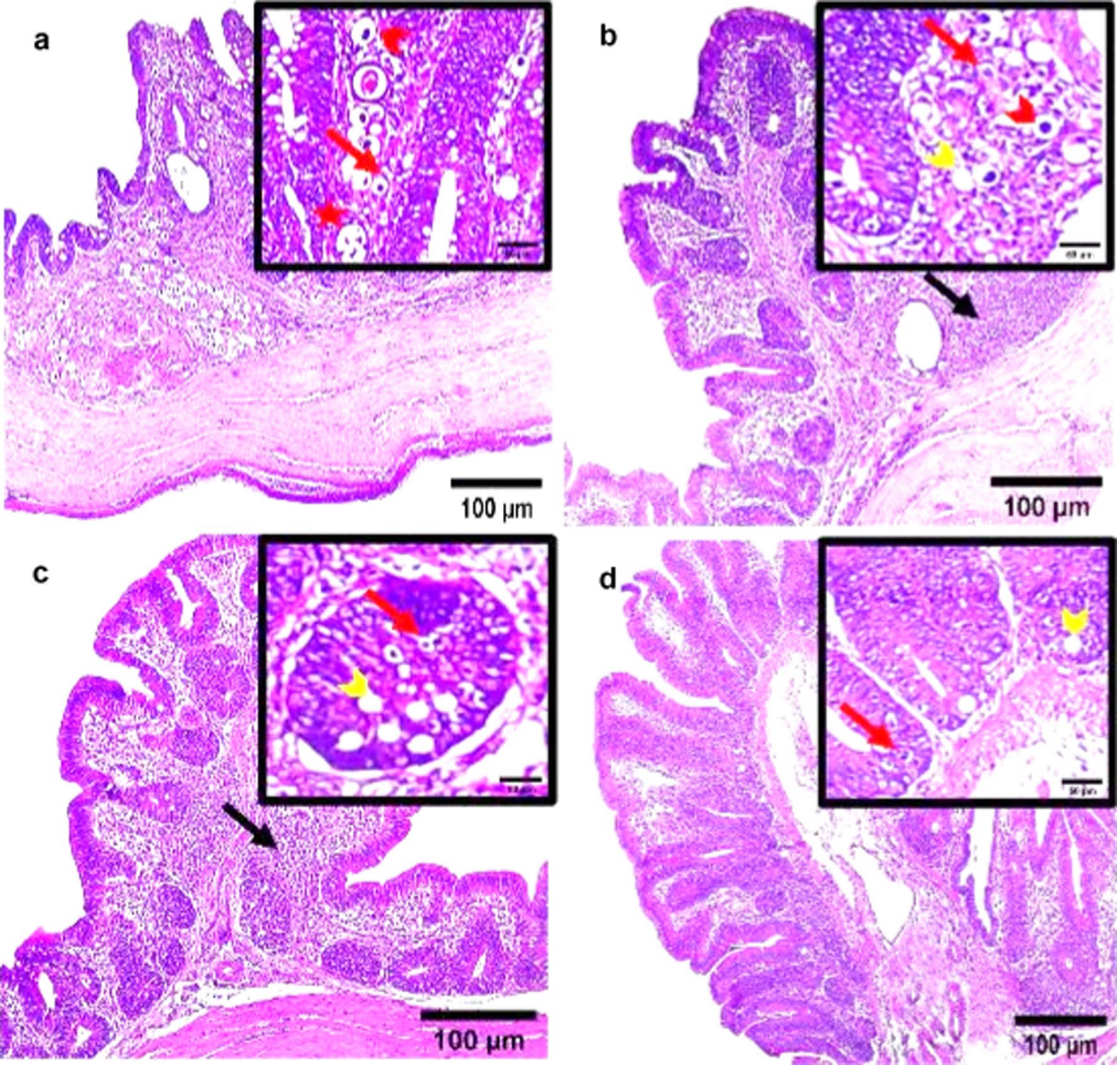
Effect of therapeutic treatment with ultrasonicated *Rosmarinus officinalis* ethanolic extract and its chitosan-loaded nanoparticles on histopathological changes of cecum of broiler chickens infected with *E. tenella* at 6 days post-secondary infection (41 days old). **a** Infected group with *E. tenella* showing obvious damaged cecal lining, desquamation of superficial epithelium with villus atrophy and marked infiltration of the mucosa with different endogenous developmental parasitic stages as schizonts (red arrowhead), merozoites (star), and oocysts (red arrow) **b** Therapeutic treatment group with ultrasonicated *Rosmarinus officinalis* ethanolic extract at 100 mg/kg B.W. showing semi-abnormality in cecal architecture with moderate amount of oocysts (red arrow), schizonts (red arrowhead), vacuolated schizonts (yellow arrowhead) and aggregated lymphocytes were also seen (black arrow). **c** Therapeutic treatment group with chitosan nanoparticles at 20 mg/kg B.W. showing semi-normality in cecal architecture with little number of oocysts (red arrow), vacuolated schizonts (yellow arrowhead) and aggregated lymphocytes (black arrow). **d** Therapeutic treatment group with ultrasonicated *Rosmarinus officinalis* ethanolic extract**-**chitosan loaded nanoparticles at 20 mg/kg B.W. showing semi-normality in cecal architecture with few oocysts (red arrow) and vacuolated schizonts (yellow arrowhead), H&E stain, Scale bar = 100 µm (color figure online)

Microscopic observations of the cecal tissue of the positive infected group with *E. tenella* at 6 DPPI appeared damaged with severe inflammation and inflammatory cells infiltration, villous atrophy, desquamation of superficial epithelium and the structure was vague. Lieberkühn’s crypts epithelium lining was infected with a remarkable enormous amount of different developmental endogenous parasitic stages including immature oocysts and multiple oval structures of schizonts containing banana-shaped merozoites occupying the sites of the absorptive epithelium (Fig. 8a). Additionally, the cecal tissue of *E. tenella* infected chickens at 6 DPSI exhibited obvious disintegration of cecal lining, sloughing off superficial epithelial cells of folds with villus atrophy and marked infiltration of the mucosa with inflammatory cells and different endogenous developmental parasitic stages including schizonts, merozoites, and oocysts (Fig. 9a). On the other hand, the three dietary prophylactic groups (P-UROEE, P-CsNPs and P-UROEE-CsNPs) showed appeared improvements in cecal tissue architecture as the shape of villi, glands and lamina propria became normal. Decreased occurrence of developmental stages as oocysts, schizonts and several abnormal vacuolated schizonts were documented in P-UROEE group at 6 DPPI (Fig. 8b) and 6 DPSI (Fig. 9b). A few oocysts and mild amount of vacuolated schizonts were shown in P-CsNPs group at 6 DPPI (Fig. 8c) and 6 DPSI (Fig. 9c), while little number of oocysts and enormous vacuolated schizonts were seen in P-UROEE-CsNPs group at 6 DPPI (Fig. 8d) and 6 DPSI (Fig. 9d). Moreover, the three therapeutic treatment groups (T-UROEE, T-CsNPs and T-UROEE-CsNPs) revealed moderate semi-normality in cecal tissue structure appearance, alleviating the observed damage in the cecum of the PC group at 6 DPT (Fig. 10) and 6 DPSI (Fig. 11). Several oocysts, vacuolated schizonts and aggregated lymphocytes were documented in T-UROEE group at 6 DPT (Fig. 10b) and 6DPSI (Fig. 11b). In addition, in T-CsNPs groups, clusters of oocysts, few schizonts and aggregation of lymphocytes were observed in cecal tissue at 6 DPT (Fig. 10c), while little number of oocysts, vacuolated schizonts and also aggregated lymphocytes were shown at 6 DPSI (Fig. 11c). Furthermore, few oocysts and large amount of vacuolated schizonts were seen in T-UROEE-CsNPs at 6 DPT (Fig. 10d) and 6 DPSI (Fig. 11d).

### Immunohistochemical Results

#### CD4^+^ and CD8^+^ T Lymphocytes Protein Expression Levels in *E. tenella*-Infected Cecum Tissues of Broiler Chickens

**Fig. 12:**
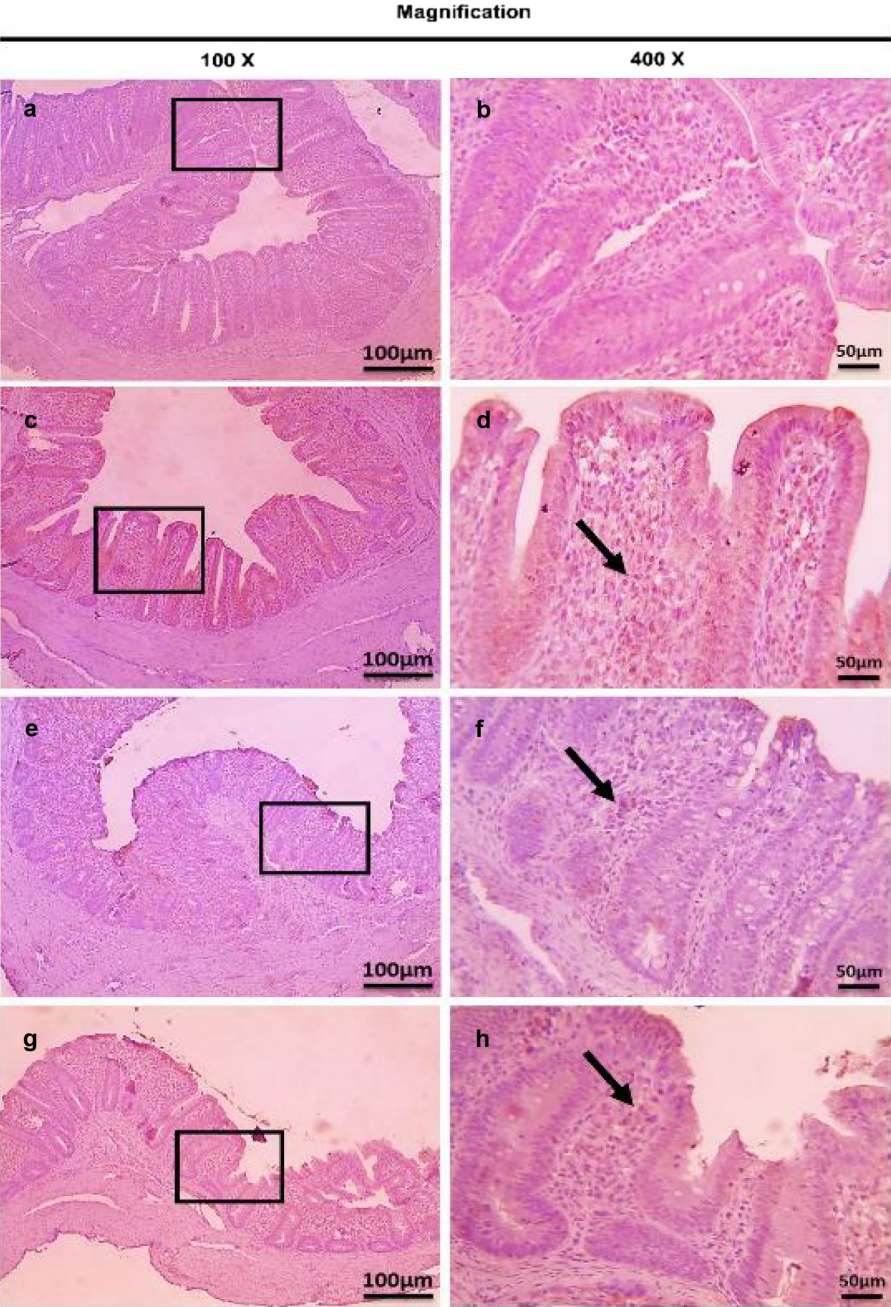
Microscopic pictures of immunostained cecal sections of broiler chickens of the supplemented groups against CD4^+^ T lymphocytes at 27 days old; showing negative expression in negative control group (**a**, 100× and **b**, 400×), very mild positive brown expression (black arrows) in dietary supplemented groups with ultrasonicated *Rosmarinus officinalis* ethanolic extract at 100 mg/kg diet (**c**, 100 × and **d**, 400×), chitosan nanoparticles at 20 mg/kg diet (**e**, 100× and **f**, 400×), ultrasonicated *Rosmarinus officinalis* ethanolic extract-chitosan loaded nanoparticles at 20 mg/kg diet (**g**, 100× and **h**, 400×). Bars = 100 µm for 100× and 50 µm for 400×

**Fig. 13:**
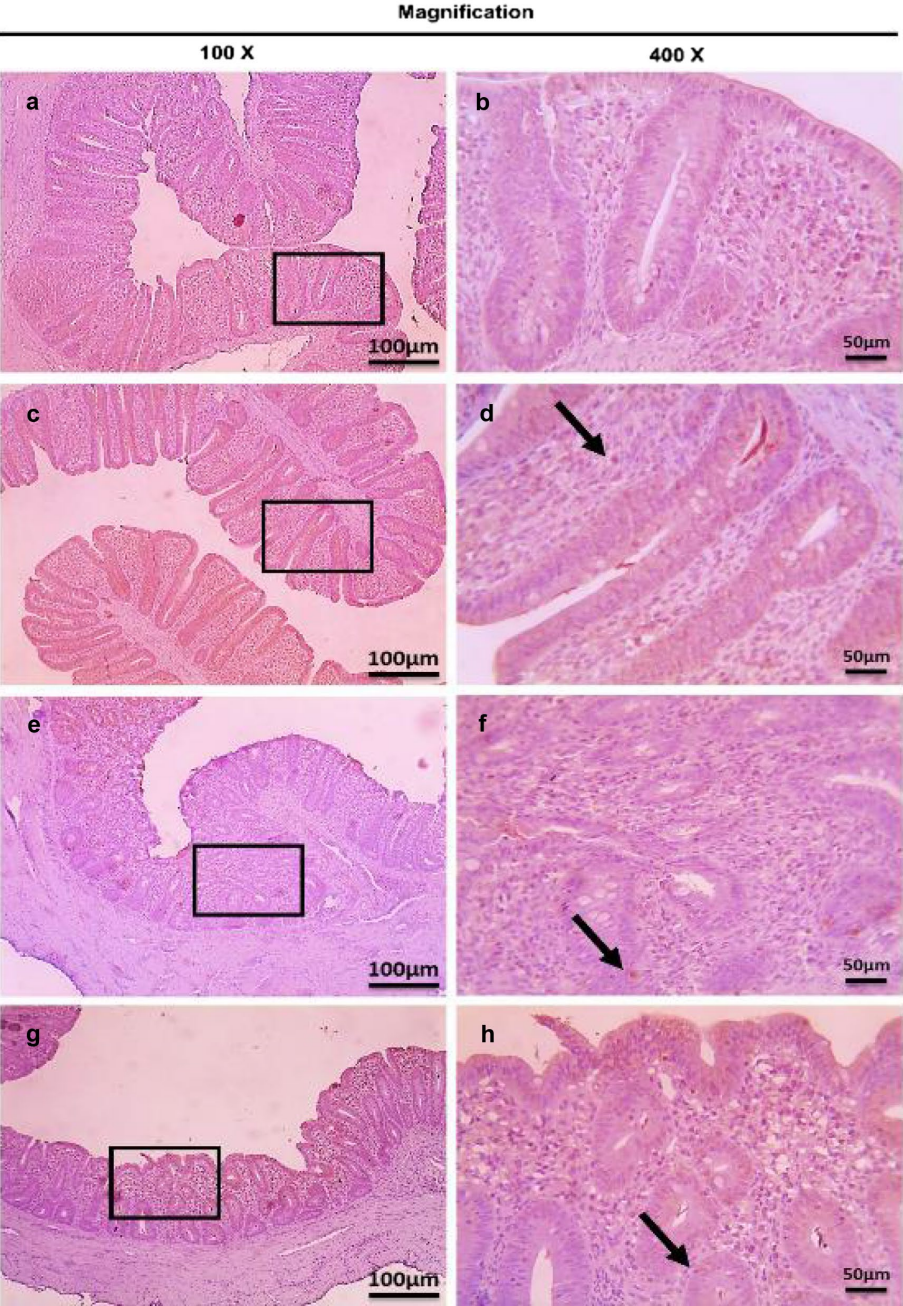
Microscopic pictures of immunostained cecal sections of broiler chickens of the supplemented groups against CD8^+^ T lymphocytes at 27 days old; showing negative expression in negative control group (**a**, 100× and **b**, 400×), very mild positive brown expression (black arrows) in dietary supplemented groups with ultrasonicated *Rosmarinus officinalis* ethanolic extract at 100 mg/kg diet (**c**, 100 × and **d**, 400×), chitosan nanoparticles at 20 mg/kg diet (**e**, 100× and **f**, 400×), and ultrasonicated *Rosmarinus officinalis* ethanolic extract-chitosan loaded nanoparticles at 20 mg/kg diet (**g**, 100× and **h**, 400×). Bars = 100 µm for 100 × and 50 µm for 400 x

**Fig. 14:**
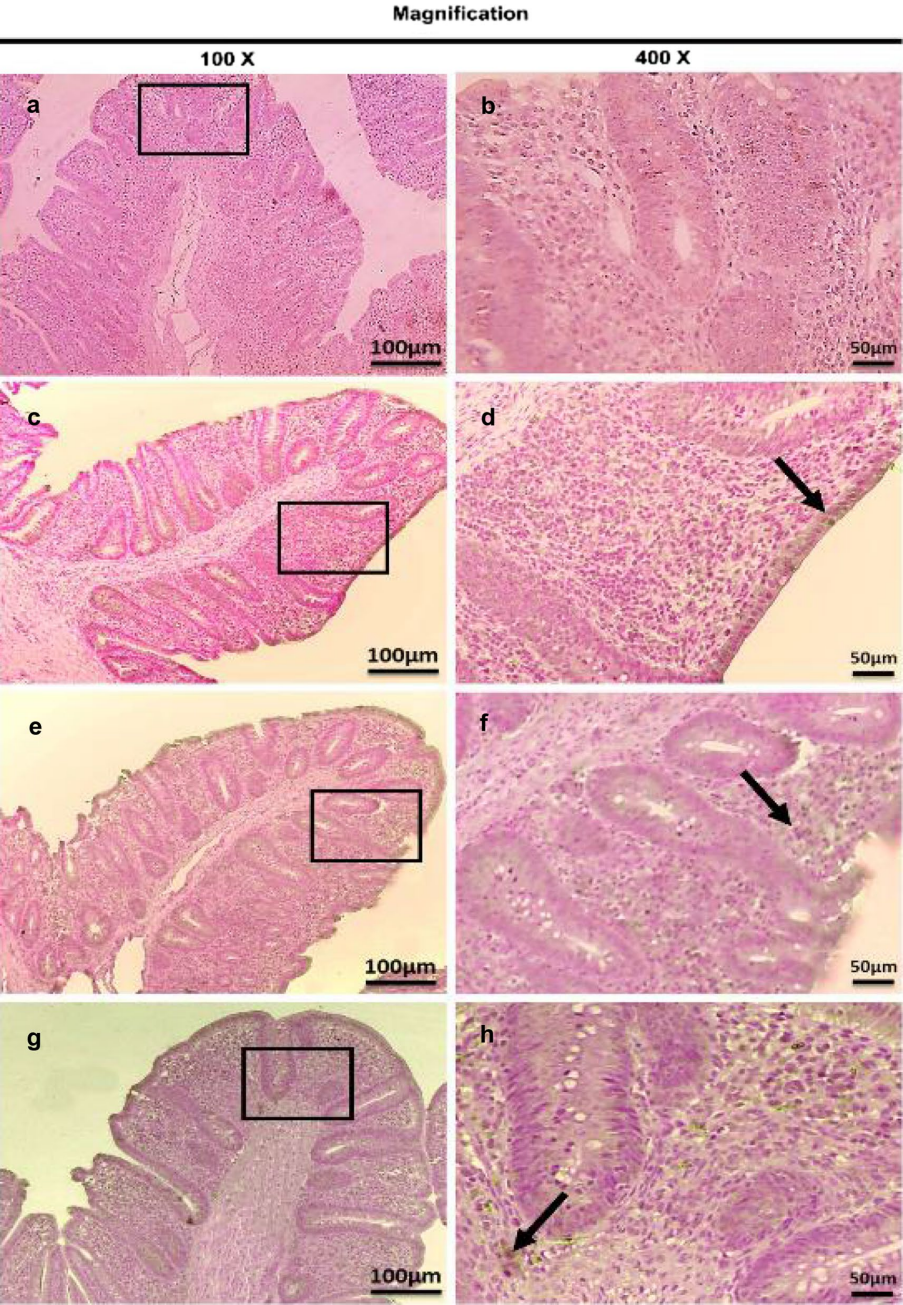
Microscopic pictures of immunostained cecal sections of broiler chickens of the supplemented groups against CD4^+^ T lymphocytes at 41 days old; showing negative expression in negative control group (**a**, 100× and **b**, 400×), very mild positive brown expression (black arrows) in dietary supplemented groups with ultrasonicated *Rosmarinus officinalis* ethanolic extract at 100 mg/kg diet (**c**, 100× and **d**, 400×), chitosan nanoparticles at 20 mg/kg diet (**e**, 100× and **f**, 400×), ultrasonicated *Rosmarinus officinalis* ethanolic extract- chitosan loaded nanoparticles at 20 mg/kg diet (**g**, 100× and **h**, 400×). Bars = 100 µm for 100× and 50 µm for 400×

**Fig. 15:**
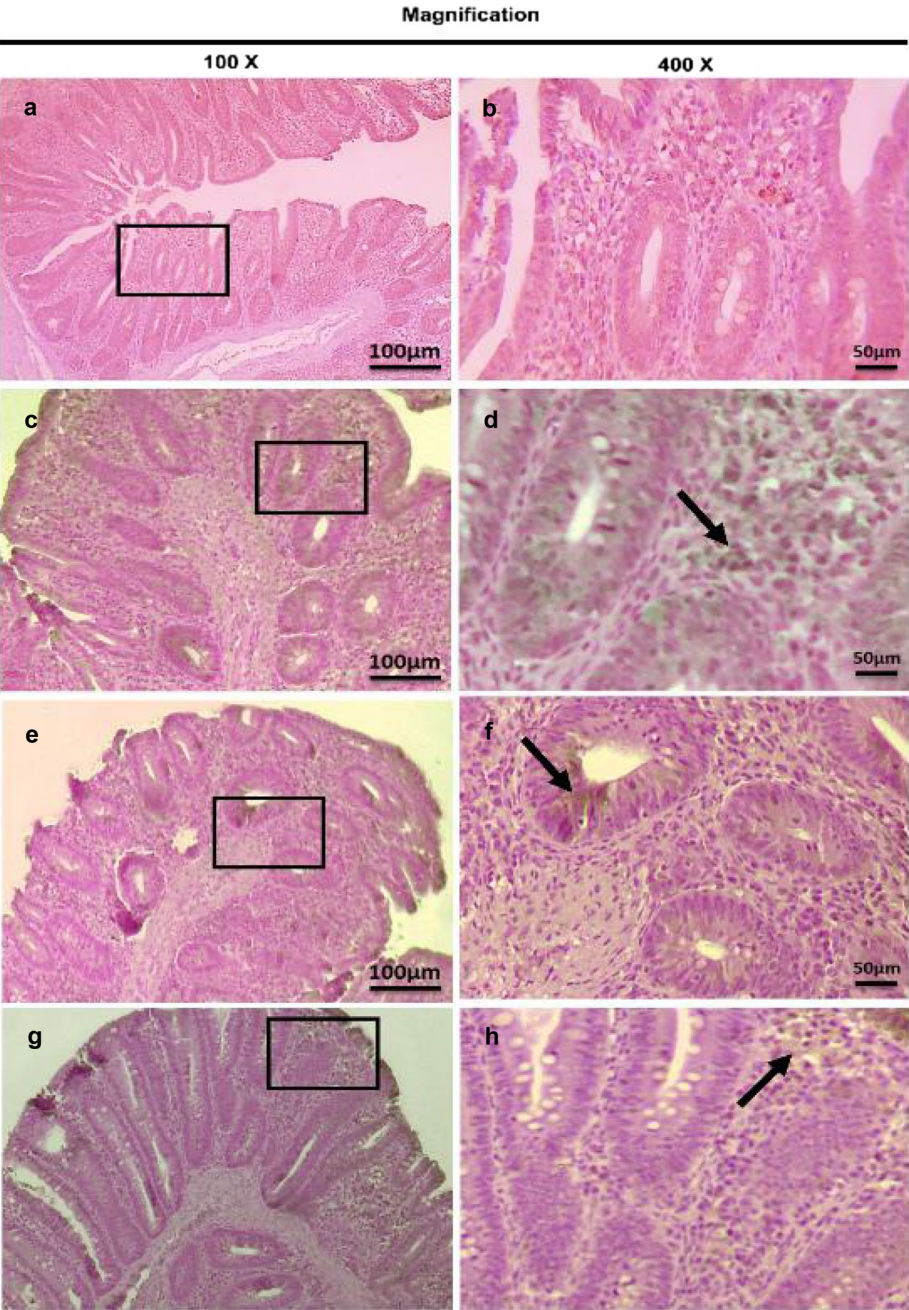
Microscopic pictures of immunostained cecal sections of broiler chickens of the supplemented groups against CD8^+^ T lymphocytes at 41 days old; showing negative expression in negative control group (**a**, 100× and **b**, 400×), very mild positive brown expression (black arrows) in dietary supplemented groups with ultrasonicated *Rosmarinus officinalis* ethanolic extract at 100 mg/kg diet (**c**, 100× and **d**, 400×), chitosan nanoparticles at 20 mg/kg diet (**e**, 100× and **f**, 400×), and ultrasonicated *Rosmarinus officinalis* ethanolic extract-chitosan loaded nanoparticles at 20 mg/kg diet (**g**, 100× and **h**, 400×). Bars = 100 µm for 100× and 50 µm for 400×

**Fig. 16:**
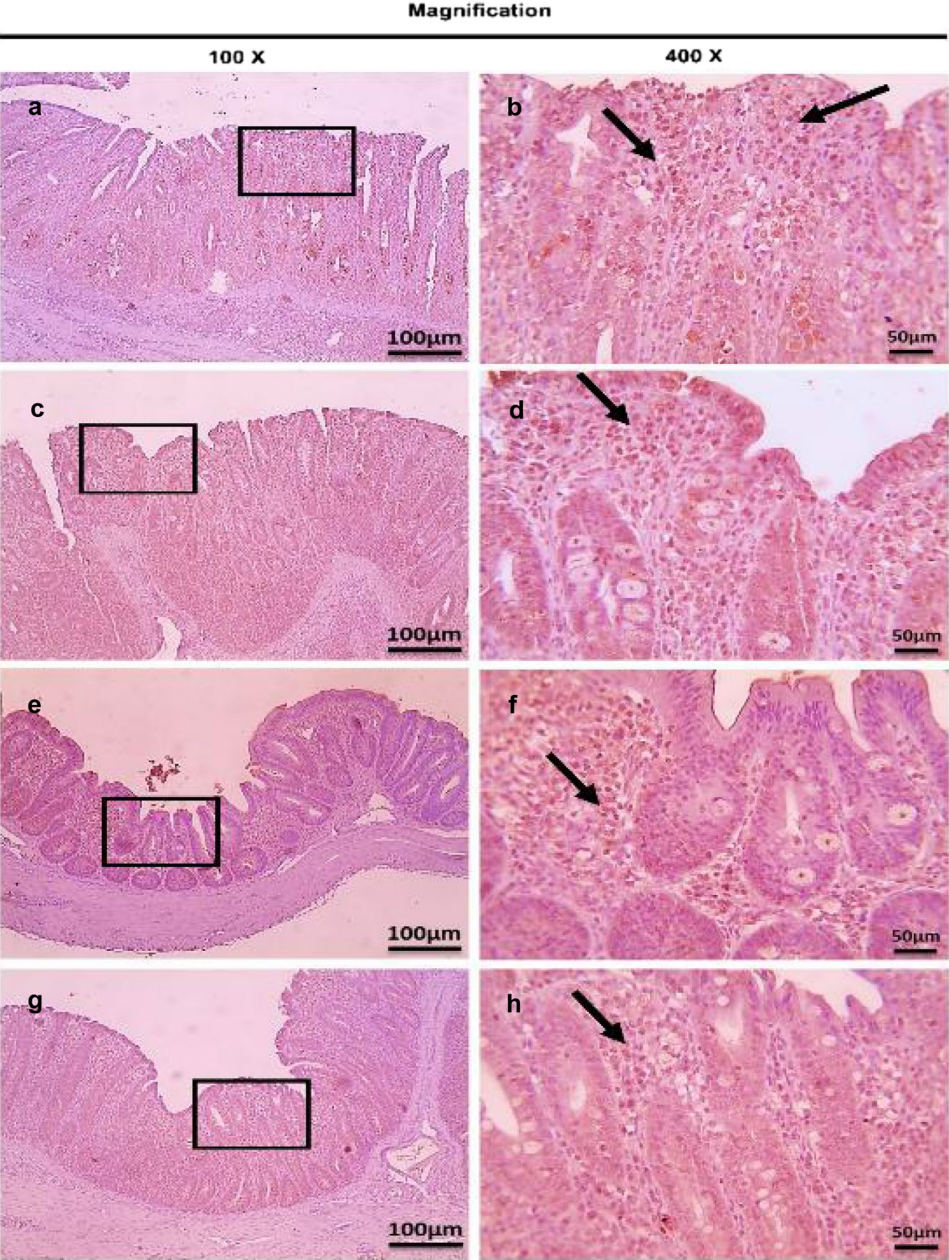
Microscopic pictures of immunostained cecal sections of broiler chickens of the dietary prophylactic groups against CD4^+^ T lymphocytes at 6 days post primary infection with *E. tenella* (27 days old); showing marked positive brown expression (black arrows) in positive control group associated with severe *E. tenella* infestation (**a**, 100× and **b**, 400×), slightly decreased positive brown expression (black arrows) in dietary prophylactic group with ultrasonicated *Rosmarinus officinalis* ethanolic extract at 100 mg/kg diet (**c**, 100× and **d**, 400×), moderately decreased positive brown expression (black arrows) in dietary prophylactic group with chitosan nanoparticles at 20 mg/kg diet (**e**, 100× and **f**, 400×), and markedly decreased positive brown expression (black arrows) in dietary prophylactic group with ultrasonicated *Rosmarinus officinalis* ethanolic extract-chitosan loaded nanoparticles at 20 mg/kg diet (**g**, 100× and **h**, 400×). Bars = 100 µm for 100× and 50 µm for 400×

**Fig. 17:**
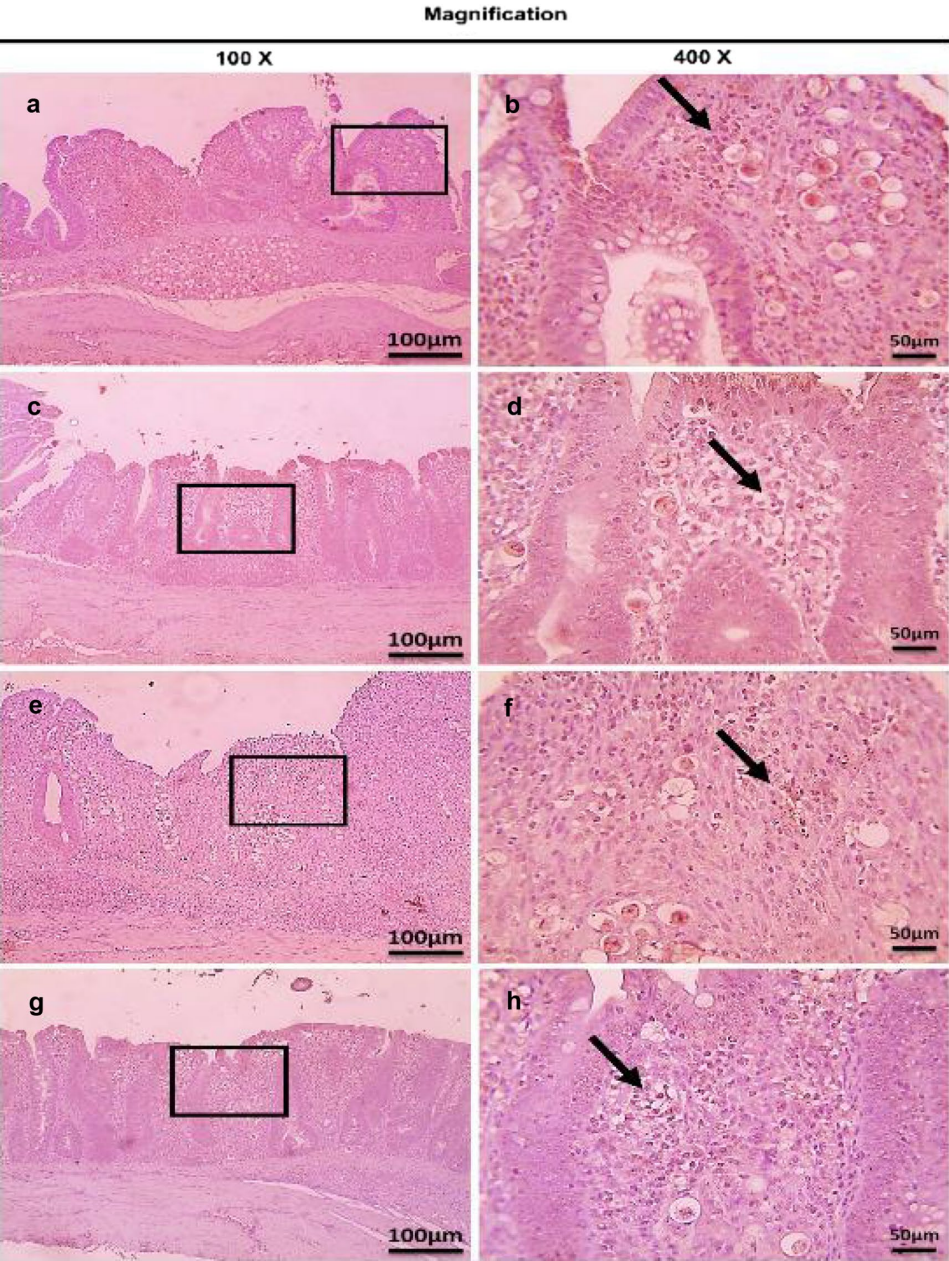
Microscopic pictures of immunostained cecal sections of broiler chickens of the dietary prophylactic groups against CD4^+^ T lymphocytes at 6 days post-secondary infection with *E. tenella* (41 days old); showing marked positive brown expression (black arrow) in positive control group associated with severe *E. tenella* infestation (**a**, 100× and **b**, 400×), markedly decreased positive brown expression (black arrow) in dietary prophylactic group with ultrasonicated *Rosmarinus officinalis* ethanolic extract at 100 mg/kg diet (**c**, 100× and **d**, 400×), slightly decreased positive brown expression (black arrow) in dietary prophylactic group with chitosan nanoparticles at 20 mg/kg diet (**e**, 100× and **f**, 400x), and markedly decreased positive brown expression (black arrow) in dietary prophylactic group with ultrasonicated *Rosmarinus officinalis* ethanolic extract-chitosan loaded nanoparticles at 20 mg/kg diet (**g**, 100× and **h**, 400×). Bars = 100 µm for 100× and 50 µm for 400×

**Fig. 18:**
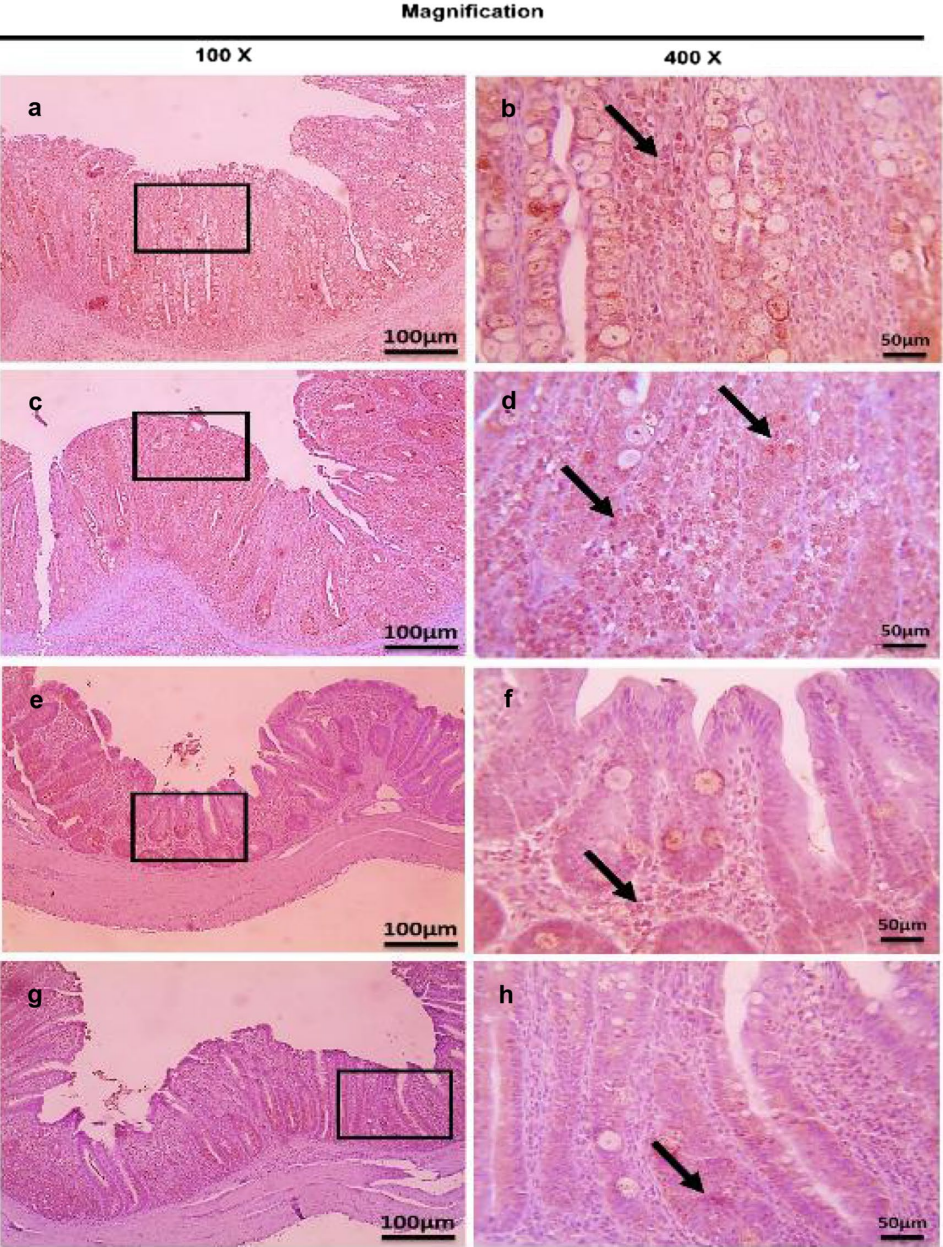
Microscopic pictures of immunostained cecal sections of broiler chickens of the dietary prophylactic groups against CD8^+^ T lymphocytes at 6 days post primary infection with *E. tenella* (27 days old); showing remarkably positive brown expression (black arrows) in positive control group associated with severe *E. tenella* infestation (**a**, 100× and **b**, 400×), slightly decreased positive brown expression (black arrows) in dietary prophylactic group with ultrasonicated *Rosmarinus officinalis* ethanolic extract at 100 mg/kg diet (**c** 100× and **d**, 400×), moderately decreased positive brown expression (black arrow) in dietary prophylactic group with chitosan nanoparticles at 20 mg/kg diet (**e**, 100× and **f**, 400×), and markedly decreased positive brown expression (black arrow) in dietary prophylactic group with ultrasonicated *Rosmarinus officinalis* ethanolic extract-chitosan loaded nanoparticles at 20 mg/kg diet (**g**, 100× and **h**, 400×). Bars = 100 µm for 100× and 50 µm for 400×

**Fig. 19:**
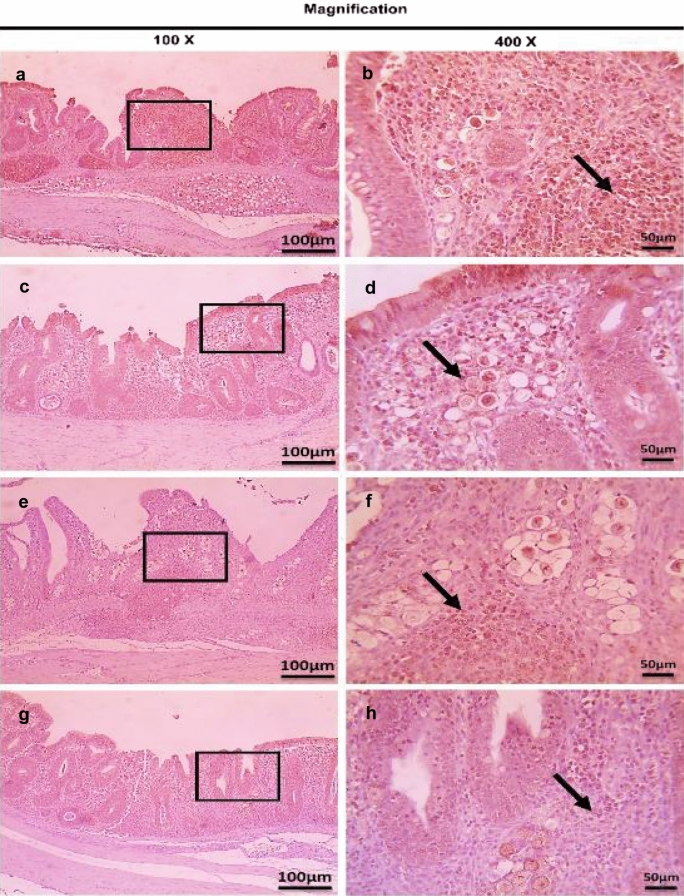
Microscopic pictures of immunostained cecal sections of broiler chickens of the dietary prophylactic groups against CD8^+^ T lymphocytes at 6 days post-secondary infection with *E. tenella* (41 days old); showing marked positive brown expression (black arrow) in positive control group associated with severe *E. tenella* infestation (**a**, 100× and **b**, 400×), moderately decreased positive brown expression (black arrows) in dietary prophylactic groups with ultrasonicated *Rosmarinus officinalis* ethanolic extract at 100 mg/kg diet (**c**, 100× and **d**, 400×) and chitosan nanoparticles at 20 mg/kg diet (**e**, 100× and **f**, 400×), and markedly decreased positive brown expression (black arrow) in dietary prophylactic group with ultrasonicated *Rosmarinus officinalis* ethanolic extract-chitosan loaded nanoparticles at 20 mg/kg diet (**g**, 100× and **h**, 400×). Bars = 100 µm for 100× and 50 µm for 400×

Immunohistochemistry was performed to determine CD4^+^ and CD8^+^ T lymphocytes protein expression in infected cecum tissues with *E. tenella*. This study showed that there were no observed significant changes (*P* > 0.05) in CD4^+^ and CD8^+^ T lymphocytes protein expression (very mild positive brown expression) in the cecum of the three dietary-supplemented groups (S-UROEE, S-CsNPs and S-UROEE-CsNPs), when compared to the NC group at 27 (Table 4, Figs. 12 and 13) and 41 days old (Table 4, Figs. 14 and 15). However, the semi-quantitative analysis revealed that there were remarkable significant increases (*P* < 0.05) in CD4^+^ T lymphocytes protein expression in the PC group that associated with severe *E. tenella* infestation in relation to the NC group at 6 DPPI (score 4.00 ± 0.00;++++) (Table 4, Fig. 16) and 6 DPSI (score 3.17 ± 0.41;+++) (Table 4, Fig. 17). The same results were also observed in CD8^+^ T lymphocytes protein expression; as the PC group exhibited highly significant (*P* < 0.05) strong positive brown expression associated with severe *E. tenella* infestation at 6 DPPI (score 3.83 ± 0.41;++++) (Table 4, Fig. 18) and 6 DPSI (score 3.00 ± 0.00;+++) (Table 4, Fig. 19) related to the NC group. Correspondingly, CD4^+^ and CD8^+^ T lymphocytes protein expression increases following primary infection than secondary infection.

**Table 4:** Average scores of semi-quantitative analysis of CD4^+^ and CD8^+^ T lymphocytes protein expression in cecum of infected broiler chickens with *E. tenella* and treated with ultrasonicated *Rosmarinus officinalis* ethanolic extract and its chitosan-loaded nanoparticles incorporated to diet (prophylactic effect)

Groups	CD4^+^ T lymphocytes		CD8^+^ T lymphocytes	
	Day 27 (6 DPPI)	Day 41 (6 DPSI)	Day 27 (6 DPPI)	Day 41 (6 DPSI)
NC	0.00 ± 0.00	0.17 ± 0.41	0.00 ± 0.00	0.00 ± 0.00
Positive control	4.00 ± 0.00^•^	3.17 ± 0.41^•^	3.83 ± 0.41^•^	3.00 ± 0.00^•^
Supplemented				
P-UROEE	0.50 ± 0.55^*^	0.50 ± 0.55^*^	0.17 ± 0.41^*^	0.17 ± 0.41
S-CsNPs	0.33 ± 0.52^*^	0.50 ± 0.55^*^	0.17 ± 0.41^*^	0.17 ± 0.41
S-UROEE-CsNPs	0.33 ± 0.52^*^	0.17 ± 0.41^*^	0.17 ± 0.41^*^	0.17 ± 0.41
Prophylactic				
P-UROEE	2.17 ± 0.75^•*^	0.83 ± 0.41^*^	2.33 ± 0.52^*^	1.33 ± 0.82^*^
P-CsNPs	1.50 ± 0.55^•*^	1.50 ± 0.55^•*^	1.67 ± 0.52^*^	1.00 ± 0.63^*^
P-UROEE-CsNPs	0.67 ± 0.52^•*^	0.83 ± 0.41^*^	0.67 ± 0.52^•*^	0.83 ± 0.41^•*^

NC; negative control group, S-UROEE; supplemented group with ultrasonicated *Rosmarinus officinalis* ethanolic extract at 100 mg/kg diet, S-CsNPs; supplemented group with chitosan nanoparticles at 20 mg/kg diet, S-UROEE-CsNPs; supplemented group with ultrasonicated *Rosmarinus officinalis* ethanolic extract- chitosan loaded nanoparticles at 20 mg/kg diet, P-UROEE; dietary prophylactic group with ultrasonicated *Rosmarinus officinalis* ethanolic extract at 100 mg/kg diet, P-CsNPs; dietary prophylactic group with chitosan nanoparticles at 20 mg/kg diet, P-UROEE-CsNPs; dietary prophylactic group with ultrasonicated *Rosmarinus officinalis* ethanolic extract- chitosan loaded nanoparticles at 20 mg/kg diet. Data are means ± standard deviation

*Significant (*P* < 0.05), when compared to positive control group. ++++highly strong (4), +++strong (3), ++moderate (2), +mild (1)

^•^Significant (*P* < 0.05), when compared to negative control group

Concerning the dietary prophylactic groups (P-UROEE, P-CsNPs and P-UROEE-CsNPs); significant (*P* < 0.05) reduction in CD4^+^ and CD8^+^ T lymphocytes protein expression was exhibited, when compared to the PC group at 6 DPPI and 6 DPSI; indicating that P-UROEE-CsNPs group had the highest remarkable reduction followed by P-CsNPs (moderately reduction) and then P-UROEE group that showed a slight reduction in CD4^+^ and CD8^+^ at 6 DPPI (Table 4, Figs. 16 and 18), while at 6 DPSI, the P-UROEE and P-UROEE-CsNPs groups demonstrated the highest reduction in CD4^+^ T lymphocytes followed by CsNPs group. Moreover, the P-UROEE and P-CsNPs groups indicated moderate reduction and P-UROEE-CsNPs group showed a marked decline in CD8^+^ T lymphocytes protein expression compared to the PC group (Table 4, Figs. 17 and 19).

**Fig. 20:**
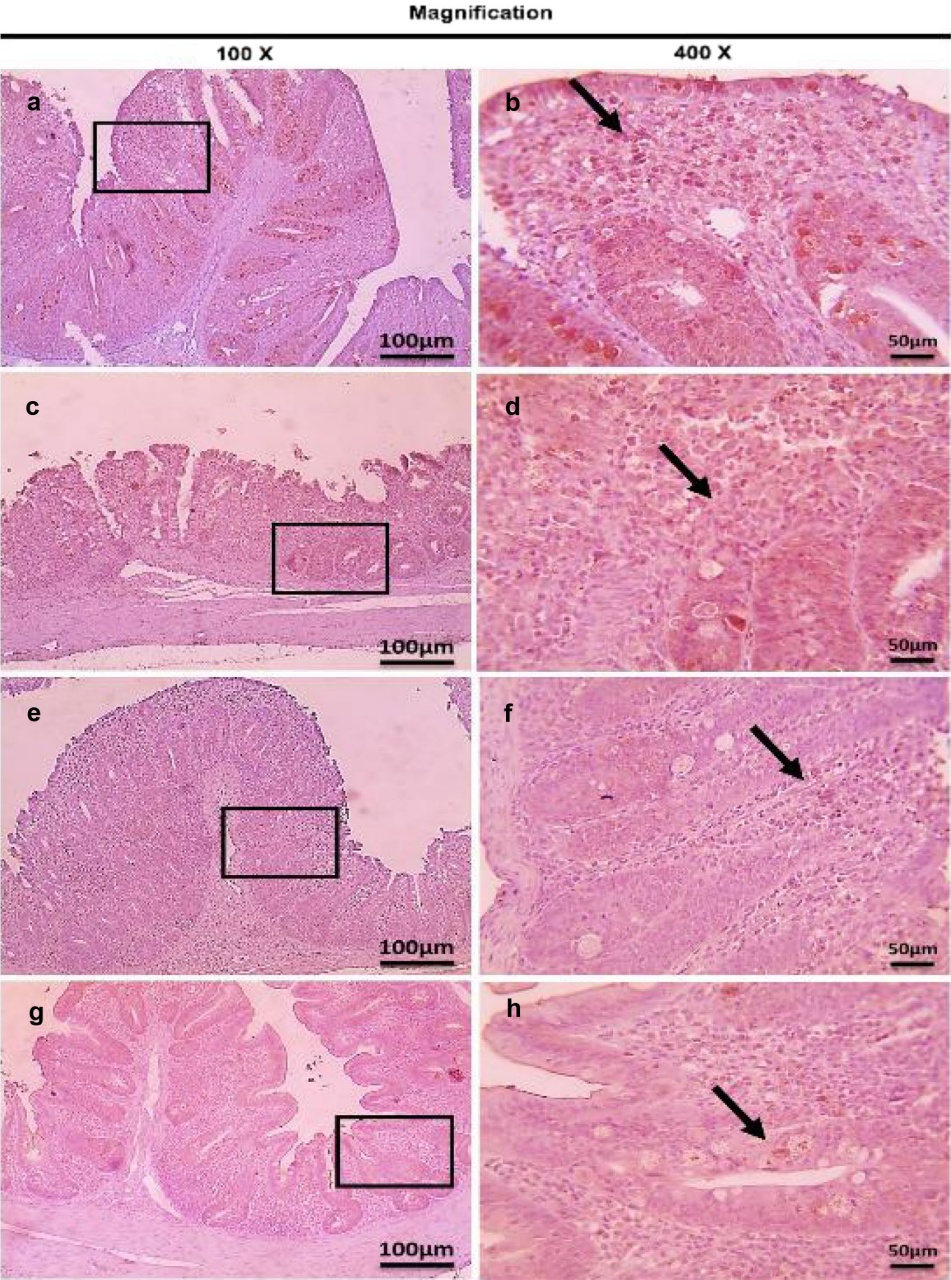
Microscopic pictures of immunostained cecal sections of infected broiler chickens with *E. tenella* in the therapeutic treatment groups against CD4^+^ T lymphocytes at 6 days post treatment (31 days old); showing marked positive brown expression (black arrow) in positive control group associated with severe *E. tenella* infestation (**a**, 100× and **b**, 400×), slightly decreased positive brown expression (black arrow) in therapeutic treatment group with ultrasonicated *Rosmarinus officinalis* ethanolic extract at 100 mg/kg B. W. (**c**, 100× and **d**, 400×), markedly decreased positive brown expression (black arrows) in therapeutic treatment groups with chitosan nanoparticles at 20 mg/kg B. W. (**e**, 100× and **f**, 400×), and ultrasonicated *Rosmarinus officinalis* ethanolic extract-chitosan loaded nanoparticles at 20 mg/kg B.W. (**g**, 100× and **h**, 400×). Bars = 100 µm for 100× and 50 µm for 400×

**Fig. 21:**
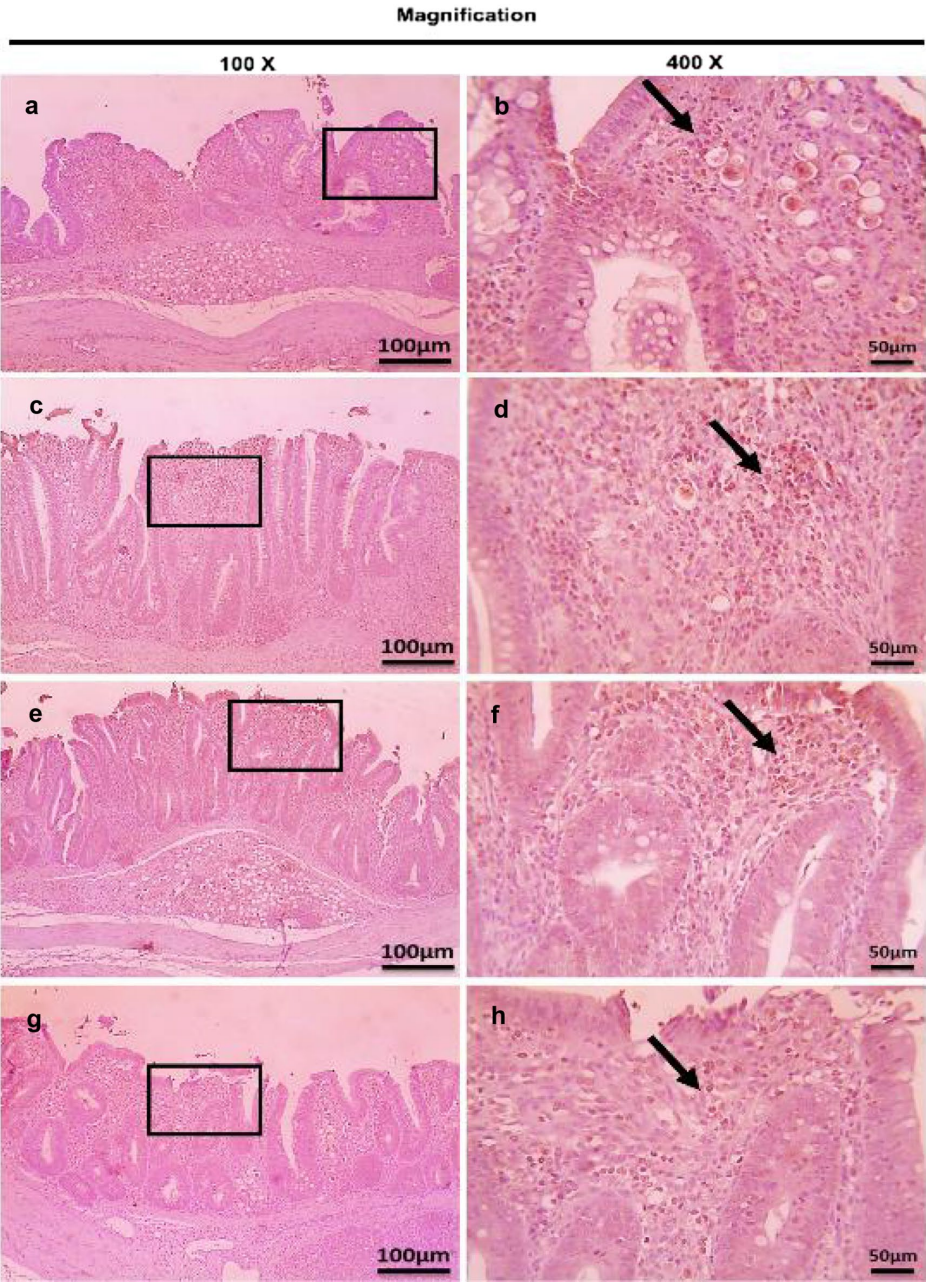
Microscopic pictures of immunostained cecal sections of infected broiler chickens with *E. tenella* in the therapeutic treatment groups against CD4^+^ T lymphocytes at 6 days post-secondary infection (41 days old); showing marked positive brown expression (black arrow) in positive control group associated with severe *E. tenella* infestation (**a**, 100× and **b**, 400×), slightly decreased positive brown expression (black arrow) in therapeutic treatment group with ultrasonicated *Rosmarinus officinalis* ethanolic extract at 100 mg/kg B. W. (**c**, 100× and **d**, 400×), moderately decreased positive brown expression (black arrow) in therapeutic treatment group with chitosan nanoparticles at 20 mg/kg B. W. (**e**, 100× and **f**, 400×), and markedly decreased positive brown expression (black arrow) in therapeutic treatment group with ultrasonicated *Rosmarinus officinalis* ethanolic extract-chitosan loaded nanoparticles at 20 mg/kg B. W. (**g**, 100× and **h**, 400×). Bars = 100 µm for 100 × and 50 µm for 400×

**Fig. 22:**
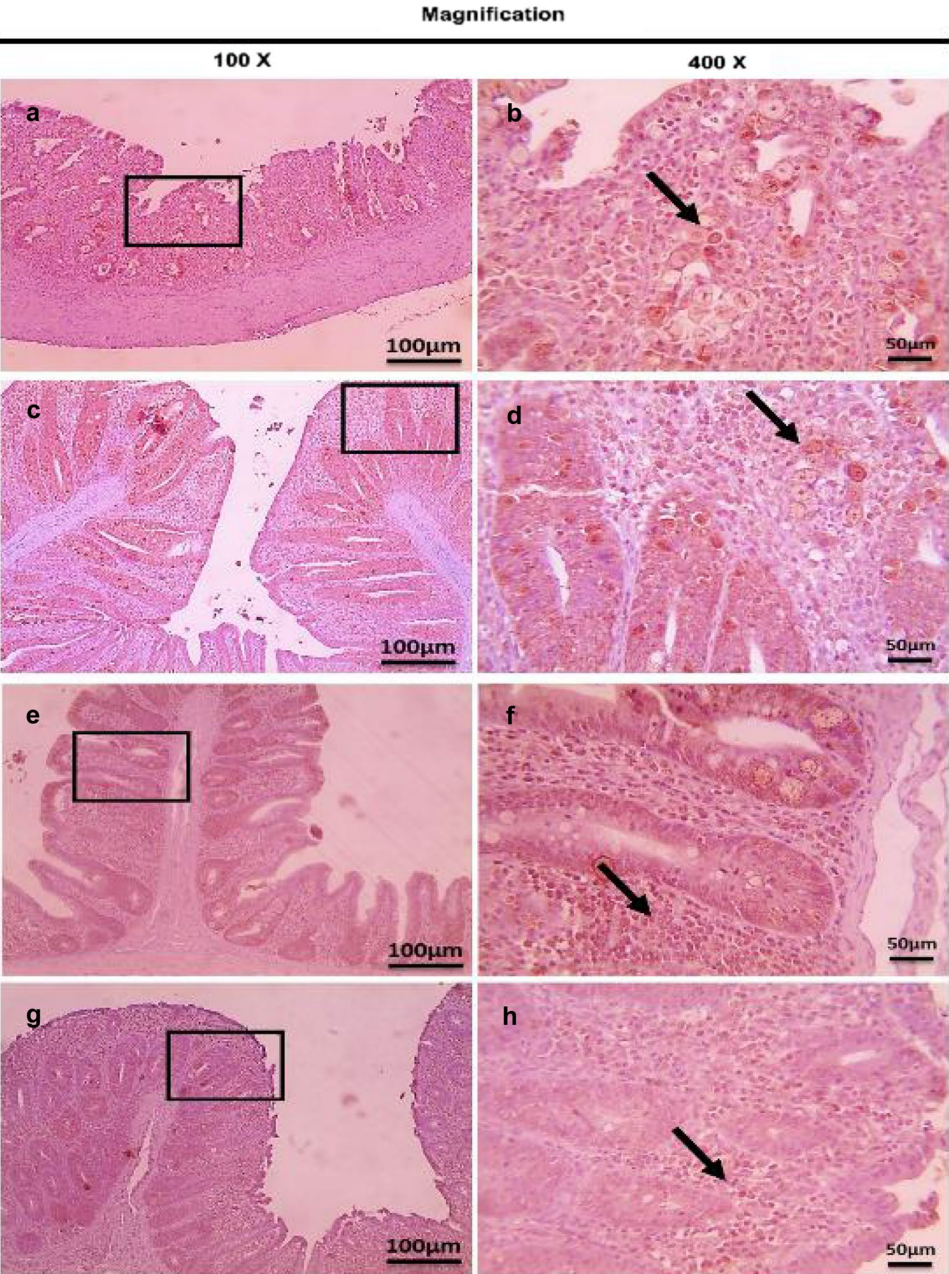
Microscopic pictures of immunostained cecal sections of infected broiler chickens with *E. tenella* in the therapeutic treatment groups against CD8^+^ T lymphocytes at 6 days post treatment (31 days old); showing marked positive brown expression (black arrow) in positive control group associated with severe *E. tenella* infestation (**a**, 100× and **b**, 400×), slightly decreased positive brown expression (black arrow) in therapeutic treatment group with ultrasonicated *Rosmarinus officinalis* ethanolic extract at 100 mg/kg B.W. (**c** 100× and **d**, 400×), moderately decreased positive brown expression (black arrows) in therapeutic treatment group with chitosan nanoparticles at 20 mg/kg B. W. (**e**, 100× and **f**, 400×) and markedly decreased positive brown expression (black arrows) in therapeutic treatment group with ultrasonicated *Rosmarinus officinalis* ethanolic extract-chitosan loaded nanoparticles at 20 mg/kg B. W. (**g**, 100× and **h**, 400×). Bars = 100 µm for 100 × and 50 µm for 400×

**Fig. 23:**
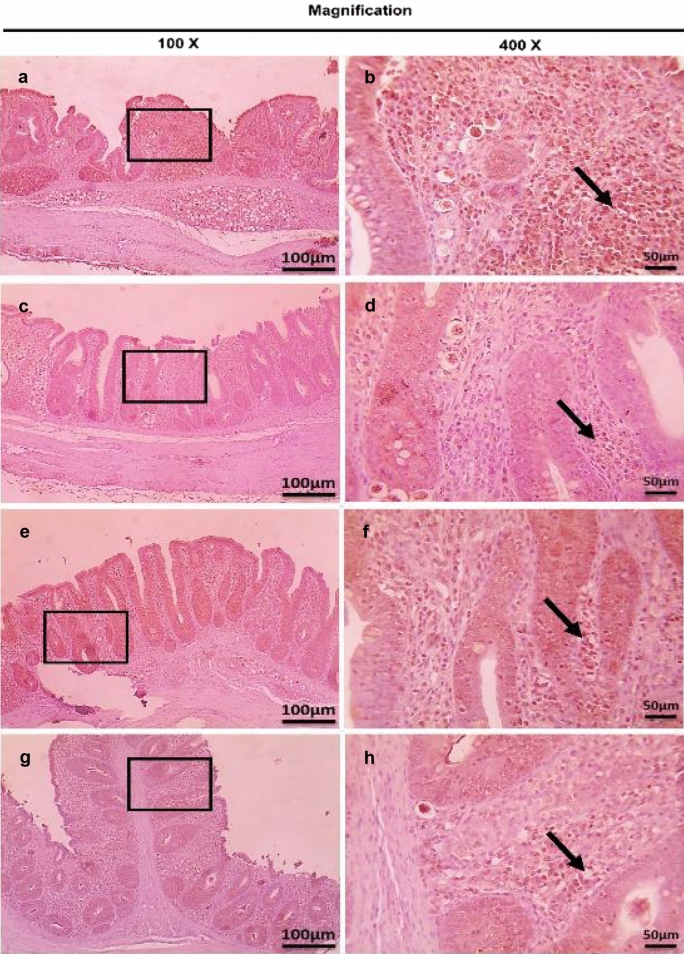
Microscopic pictures of immunostained cecal sections of infected broiler chickens with *E. tenella* in the therapeutic treatment groups against CD8^+^ T lymphocytes at 6 days post-secondary infection (41 days old); showing marked positive brown expression (black arrow) in positive control group associated with severe *E. tenella* infestation (**a**, 100× and **b**, 400×), moderately decreased positive brown expression (black arrow) in therapeutic treatment group with ultrasonicated *Rosmarinus officinalis* ethanolic extract at 100 mg/kg B. W. (**c**, 100× and **d**, 400×), and chitosan nanoparticles at 20 mg/kg B. W. (**e**, 100× and **f**, 400×), and markedly decreased positive brown expression (black arrow) in therapeutic treatment group with ultrasonicated *Rosmarinus officinalis* ethanolic extract-chitosan loaded nanoparticles at 20 mg/kg B. W. (**g**, 100× and **h**, 400×). Bars = 100 µm for 100 × and 50 µm for 400×

On the other hand, the therapeutic treatment group; T-UROEE showed significant (*P* < 0.05) slight decrease in CD4^+^ T lymphocytes, while T-CsNPs and T-UROEE-CsNPs showed a significant (*P* < 0.05) notable reduction in CD4^+^ T lymphocytes expression at 6 DPT in comparison to the PC group that showed a highly strong expression due to the highly infected cecal tissue with *E. tenella* at 6 DPT (Table 5, Fig. 20). At 6 DPSI, the therapeutic T-UROEE-CsNPs group had the highest remarkable reduction followed by T-CsNPs (moderately reduction) and then T-UROEE group that showed slight reduction in CD4^+^ T lymphocytes expression compared to the PC group (Table 5, Fig. 21). Additionally, the three therapeutic treatment groups (T-UROEE, T-CsNPs and T-UROEE-CsNPs); displayed significant (*P* < 0.05) reduction in CD8^+^ T lymphocytes protein expression, when compared to the PC group at 6 DPT indicating that P-UROEE-CsNPs group had the highest remarkable reduction followed by P-CsNPs and then P-UROEE group (Table 5, Fig. 22). At 6 DPSI, therapeutic treatment groups (T-UROEE, T-CsNPs) showed moderately decrease in CD8^+^ T lymphocytes protein expression and T-UROEE-CsNPs group revealed markedly decrease in CD8^+^ T lymphocytes protein expression when compared to the PC group (Table 5, Fig. 23).

**Table 5:** Average scores of semi-quantitative analysis of CD4^+^ and CD8^+^ T lymphocytes protein expression in cecum of infected broiler chickens with *E. tenella* and treated with ultrasonicated *Rosmarinus officinalis* ethanolic extract and its chitosan-loaded nanoparticles added/ Kg body weight (therapeutic effect)

Groups	CD4^+^ T lymphocytes		CD8^+^ T lymphocytes	
	Day 31 (6 DPT)	Day 41 (6 DPSI)	Day 31 (6 DPT)	Day 41 (6 DPSI)
NC	0.17 ± 0.41	0.17 ± 0.41	0.00 ± 0.00	0.00 ± 0.00
Positive control	4.00 ± 0.00^•^	3.17 ± 0.41^•^	3.67 ± 0.52^•^	3.00 ± 0.00^•^
T-UROEE	2.50 ± 0.55^*^	2.67 ± 0.52^*^	2.67 ± 0.52^•*^	2.00 ± 0.89^•*^
T-CsNPs	0.83 ± 0.41^•*^	1.50 ± 1.05^*^	2.00 ± 0.89^•*^	2.17 ± 0.41^•*^
T-UROEE-CsNPs	0.83 ± 0.41^•*^	0.67 ± 0.52^•*^	0.67 ± 0.52^*^	0.83 ± 0.75^*^

NC; negative control group, T-UROEE; therapeutic treatment group with ultrasonicated *Rosmarinus officinalis* ethanolic extract at 100 mg/kg B.W., T-CsNPs; therapeutic treatment group with chitosan nanoparticles at 20 mg/kg B.W., T-UROEE-CsNPs at 20 mg/kg B.W.; therapeutic treatment group with ultrasonicated *Rosmarinus officinalis* ethanolic extract- chitosan loaded nanoparticles. Data are means ± standard deviation

^*^Significant (*P* < 0.05), when compared to positive control group. ++++highly strong (4), +++strong (3), ++moderate (2), +mild (1)

^•^Significant (*P* < 0.05), when compared to negative control group

### Gene Expression of Pro-inflammatory Cytokines in Cecum of *E. tenella* Infected Chickens

**Fig. 24:**
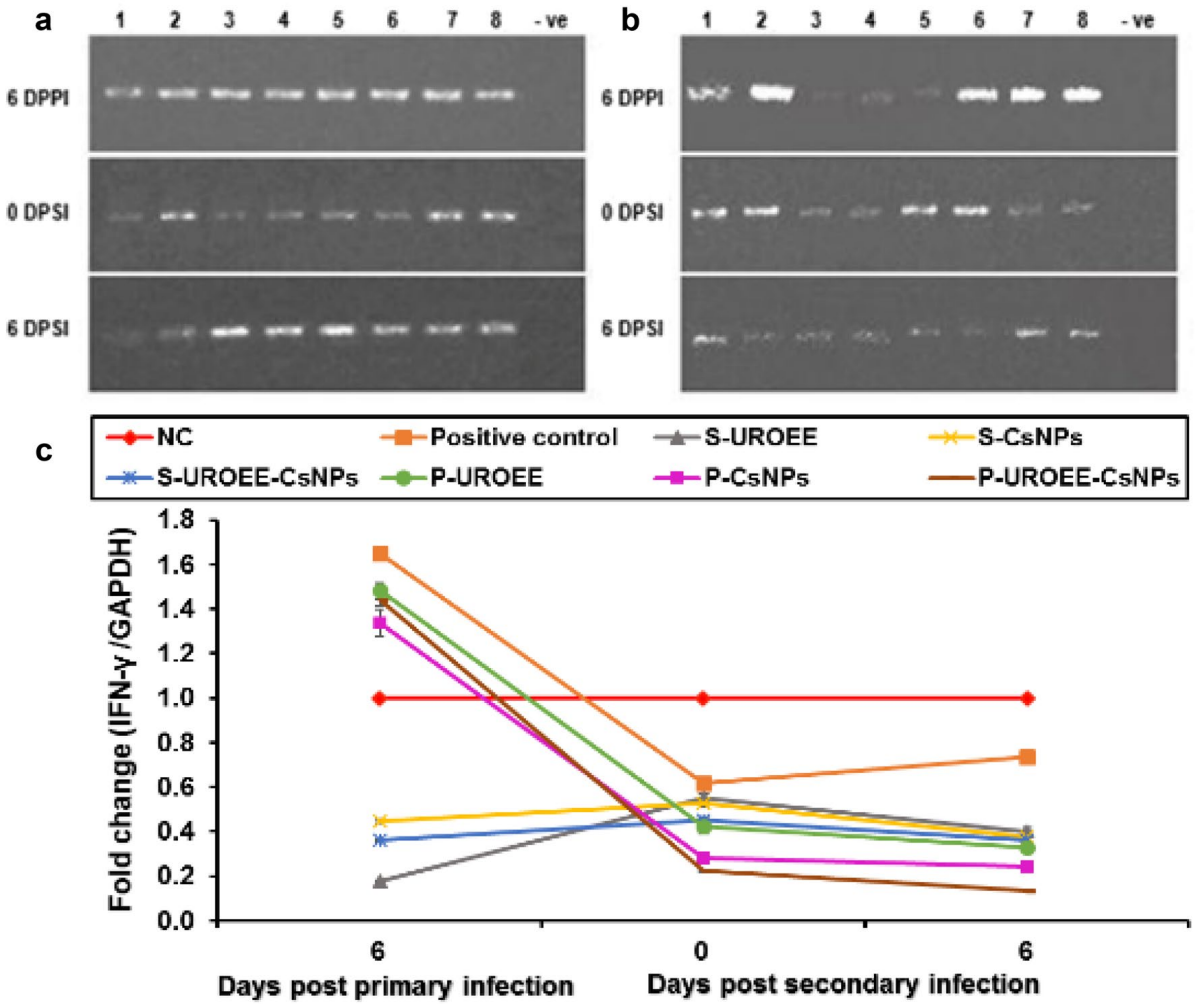
Agarose gel electrophoresis of semi-quantitative RT-PCR for chicken **a** GAPDH and **b** IFN-γ in the cecum of the dietary prophylactic groups at 6 days post-primary infection as well as at 0 and 6 days post-secondary infection with *E. tenella*. **c** Semi-quantitative RT-PCR analysis of chicken IFN-γ/GAPDH in the cecum. The expression values were obtained by semi-quantitative RT-PCR analysis were normalized to the GAPDH mRNA level and are shown as fold of change relative to the mRNA level in the negative control group. Values are mean ± SD. Lane 1: Negative control group, Lane 2: Positive control group, Lane 3: Dietary supplemented group with ultrasonicated *Rosmarinus officinalis* ethanolic extract at 100 mg/kg, Lane 4: Dietary supplemented group with chitosan nanoparticles at 20 mg/kg, Lane 5: Dietary supplemented group with ultrasonicated *Rosmarinus officinalis* ethanolic extract-chitosan loaded nanoparticles at 20 mg/kg, Lane 6: Dietary prophylactic group with ultrasonicated *Rosmarinus officinalis* ethanolic extract at 100 mg/kg diet, Lane 7: Dietary prophylactic group with chitosan nanoparticles at 20 mg/kg diet, Lane 8: Dietary prophylactic group with ultrasonicated *Rosmarinus officinalis* ethanolic extract**-**chitosan loaded nanoparticles at 20 mg/kg diet, –ve: Negative PCR reaction

**Fig. 25:**
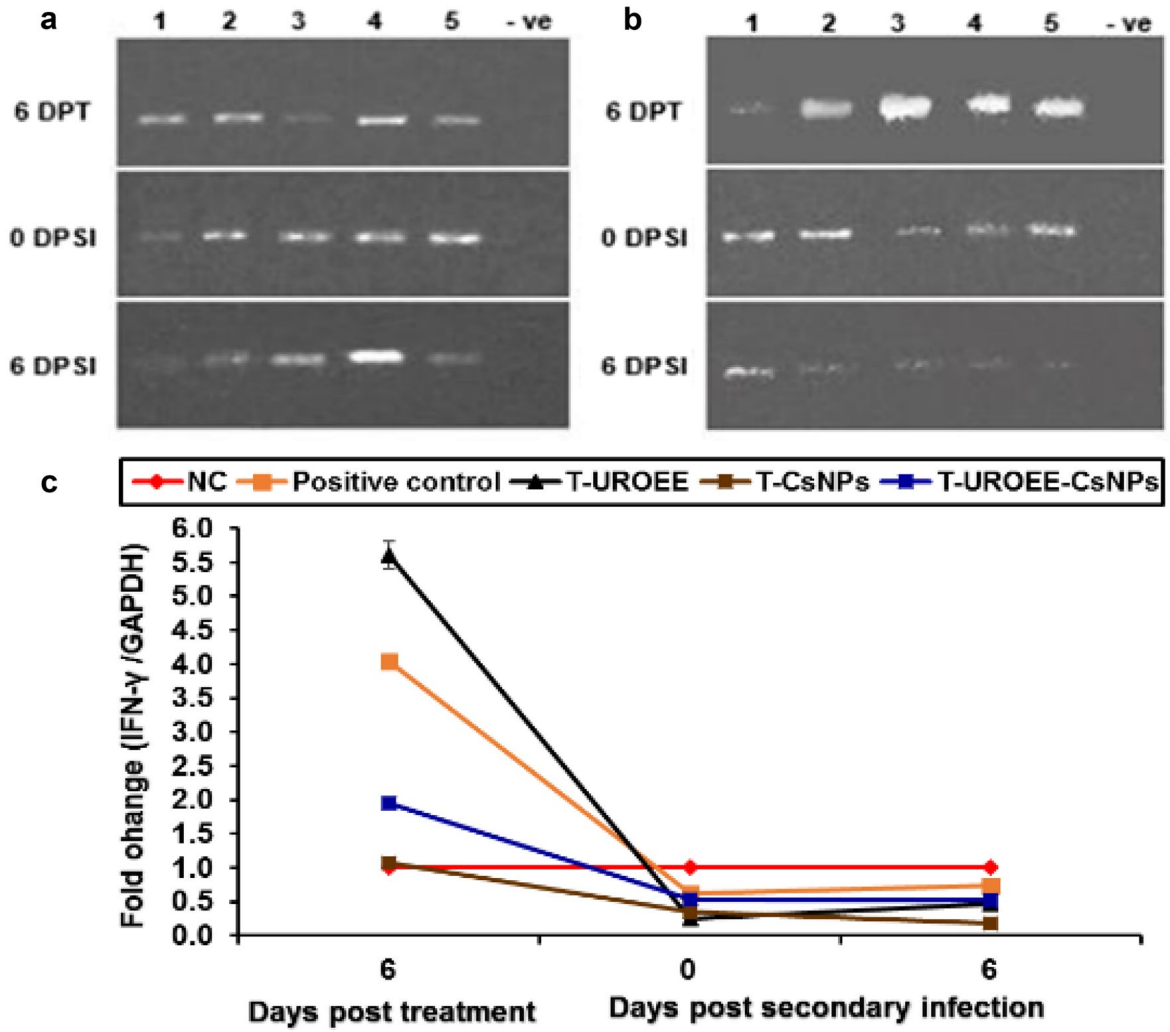
Agarose gel electrophoresis of semi-quantitative RT-PCR for chicken **a** GAPDH and** b** IFN-γ in the cecum of the therapeutic treatment groups at 6 days post treatment as well as at 0 and 6 days post-secondary infection with *E. tenella*. **c** Semi-quantitative RT-PCR analysis of chicken IFN-γ/GAPDH in the cecum. The expression values were obtained by semi-quantitative RT-PCR analysis were normalized to the GAPDH mRNA level and are shown as fold of change relative to the mRNA level in the negative control group. Values are mean ± SD. Lane 1: Negative control group, Lane 2: Positive control group, Lane 3: Therapeutic treatment group with ultrasonicated *Rosmarinus officinalis* ethanolic extract at 100 mg/kg B.W., Lane 4: Therapeutic treatment group with chitosan nanoparticles at 20 mg/kg B.W., Lane 5: Therapeutic treatment group with ultrasonicated *Rosmarinus officinalis* ethanolic extract-chitosan loaded nanoparticles at 20 mg/kg B.W., –ve: Negative PCR reaction

**Fig. 26:**
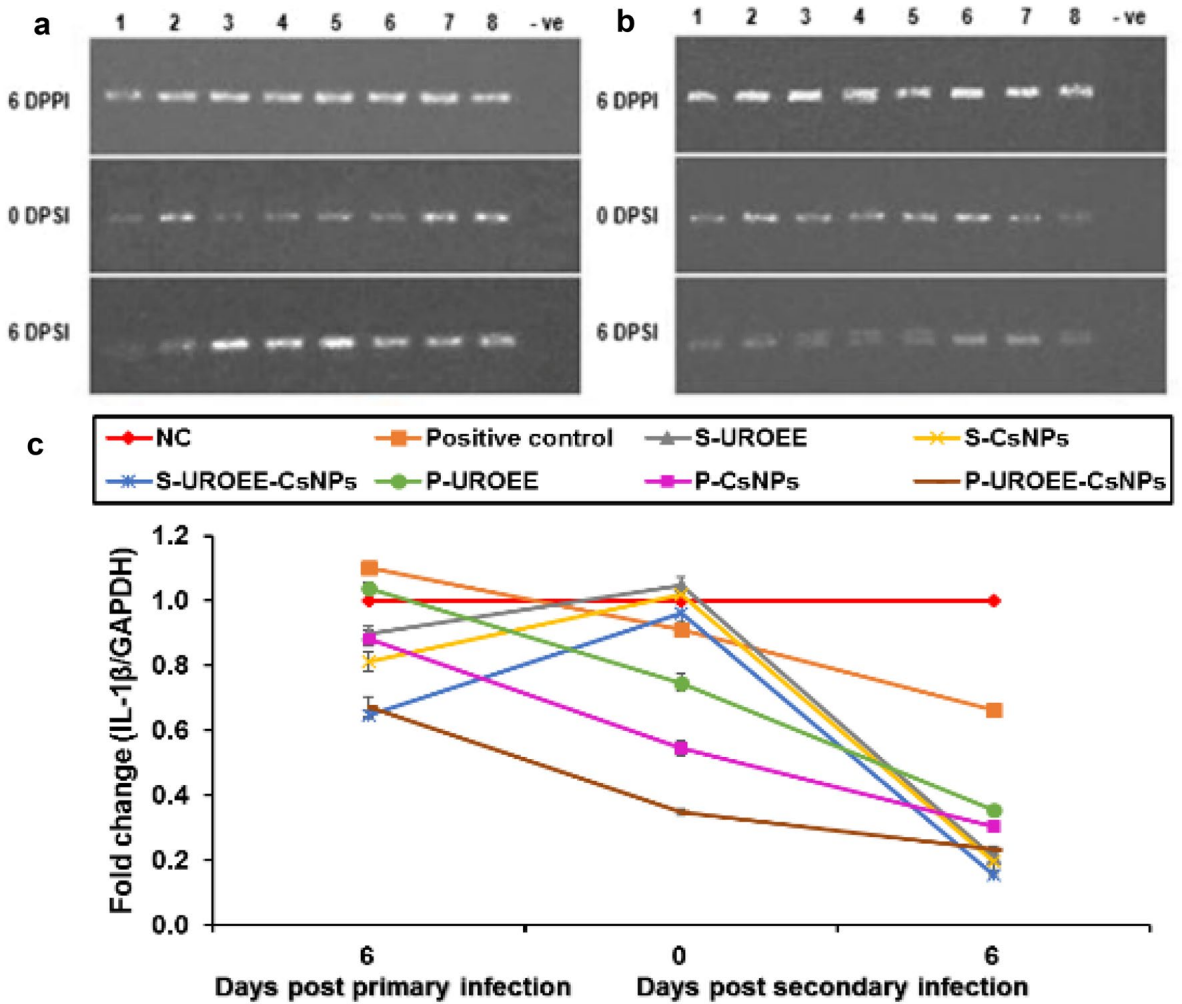
Agarose gel electrophoresis of semi-quantitative RT-PCR for chicken **a** GAPDH and **b** IL-1β in the cecum of the dietary prophylactic groups at 6 days post-primary infection as well as at 0 and 6 days post-secondary infection with *E. tenella*. **c** Semi- quantitative RT-PCR analysis of chicken IL-1β/GAPDH in the cecum. The expression values were obtained by semi-quantitative RT-PCR analysis were normalized to the GAPDH mRNA level and are shown as fold of change relative to the mRNA level in the negative control group. Values are mean ± SD. Lane 1: Negative control group, Lane 2: Positive control group, Lane 3: Dietary supplemented group with ultrasonicated *Rosmarinus officinalis* ethanolic extract at 100 mg/kg, Lane 4: Dietary supplemented group with chitosan nanoparticles at 20 mg/kg, Lane 5: Dietary supplemented group with ultrasonicated *Rosmarinus officinalis* ethanolic extract-chitosan loaded nanoparticles at 20 mg/kg, Lane 6: Dietary prophylactic group with ultrasonicated *Rosmarinus officinalis* ethanolic extract at 100 mg/kg diet, Lane 7: Dietary prophylactic group with chitosan nanoparticles at 20 mg/kg diet, Lane 8: Dietary prophylactic group with ultrasonicated *Rosmarinus officinalis* ethanolic extract-chitosan loaded nanoparticles at 20 mg/kg diet, –ve: Negative PCR reaction

**Fig. 27:**
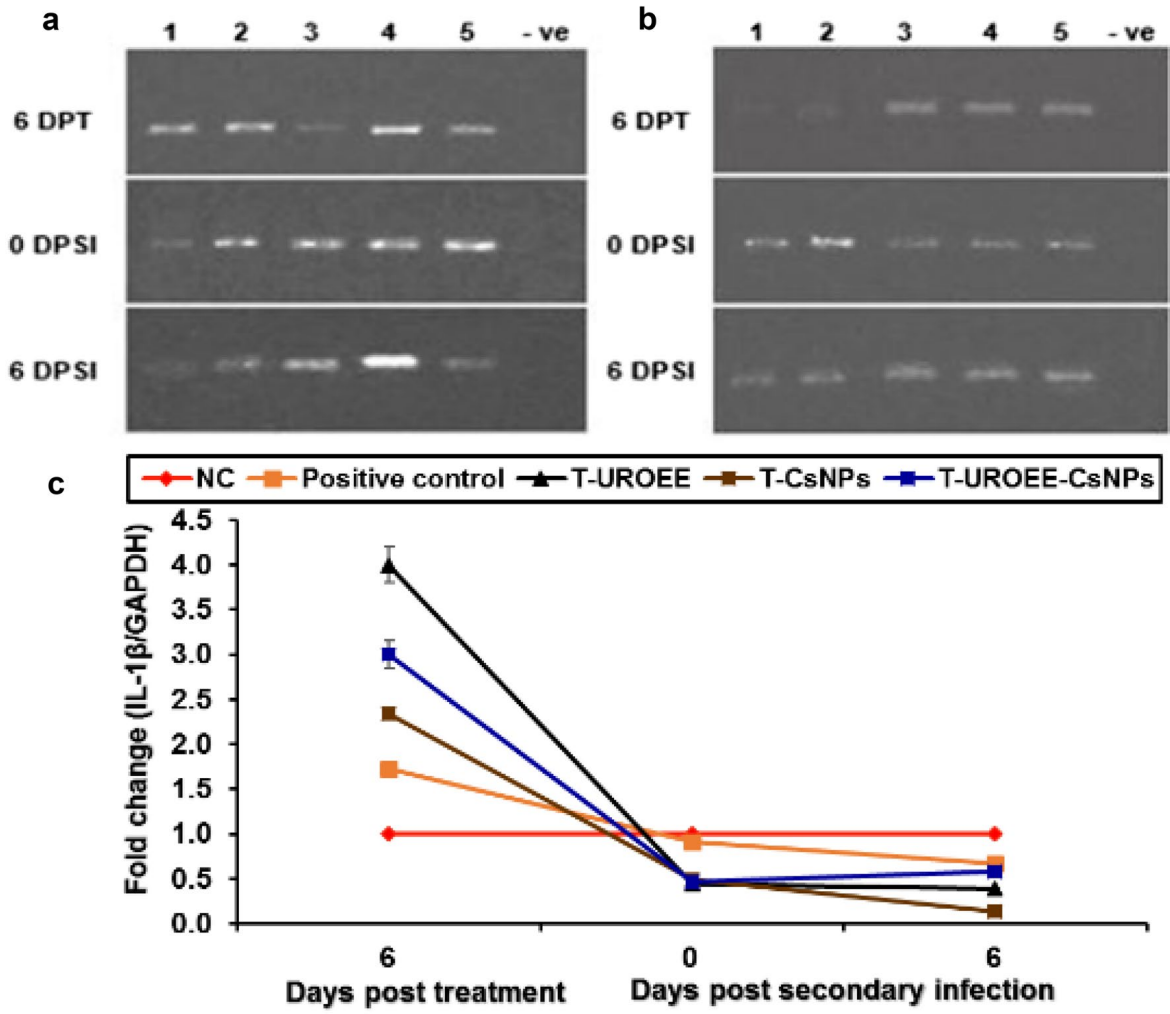
Agarose gel electrophoresis of semi-quantitative RT-PCR for chicken **a** GAPDH and **b** IL-1β in the cecum of the therapeutic treatment groups at 6 days post treatment as well as at 0 and 6 days post-secondary infection with *E. tenella*. **c** Semi-quantitative RT-PCR analysis of chicken IL-1β/GAPDH in the cecum. The expression values were obtained by semi-quantitative RT-PCR analysis were normalized to the GAPDH mRNA level and are shown as fold of change relative to the mRNA level in the negative control group. Values are mean ± SD. Lane 1: Negative control group, Lane 2: Positive control group, Lane 3: Therapeutic treatment group with ultrasonicated *Rosmarinus officinalis* ethanolic extract at 100 mg/kg B.W., Lane 4: Therapeutic treatment group with chitosan nanoparticles at 20 mg/kg B.W., Lane 5: Therapeutic treatment group with ultrasonicated *Rosmarinus officinalis* ethanolic extract-chitosan loaded nanoparticles at 20 mg/kg B.W., –ve: Negative PCR reaction

**Fig. 28:**
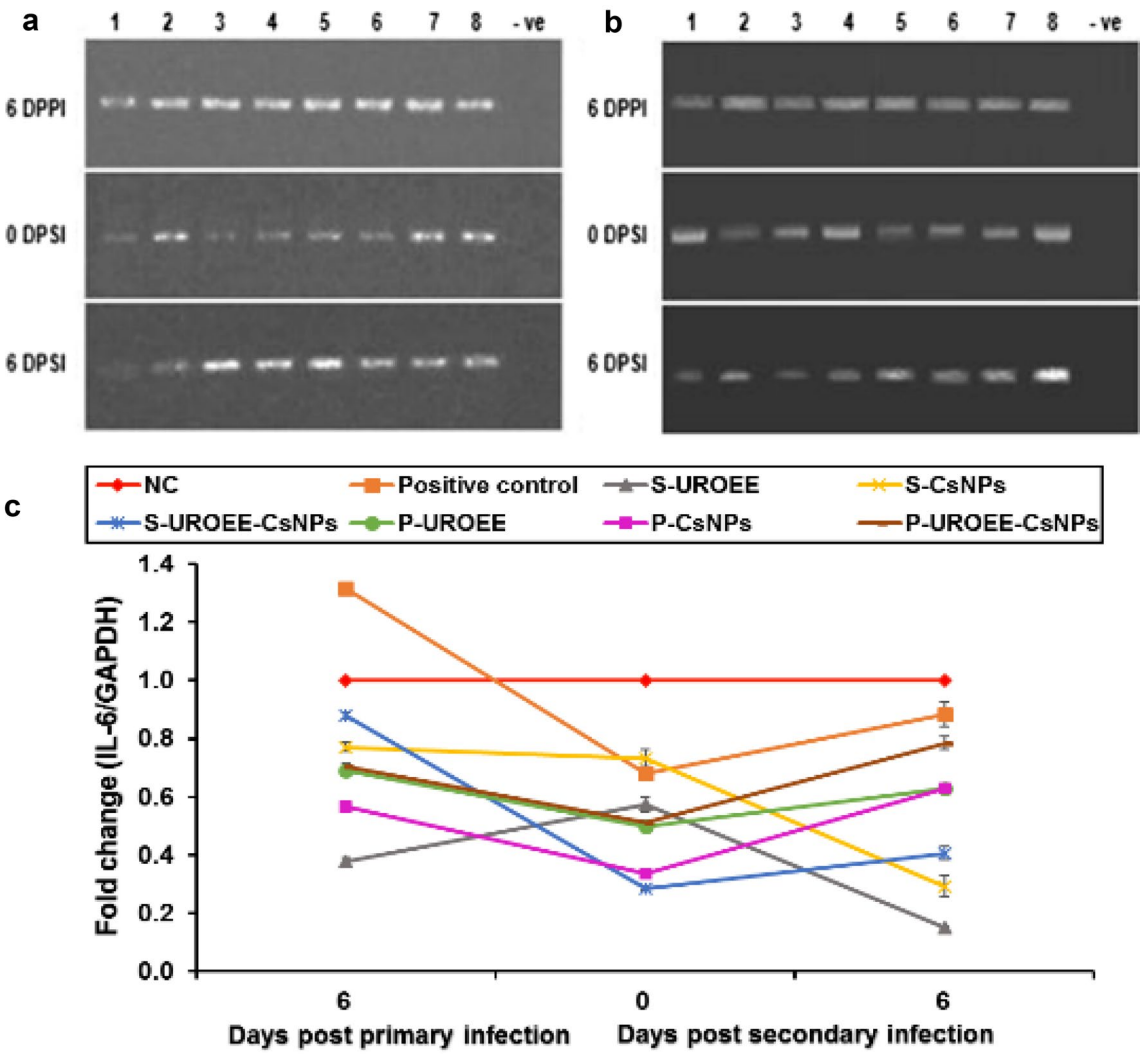
Agarose gel electrophoresis of semi-quantitative RT-PCR for chicken **a** GAPDH and **b** IL-6 in the cecum of the dietary prophylactic groups at 6 days post-primary infection as well as at 0 and 6 days post-secondary infection with *E. tenella*. **c** Semi-quantitative RT-PCR analysis of chicken IL-6/GAPDH in the cecum. The expression values were obtained by semi-quantitative RT-PCR analysis were normalized to the GAPDH mRNA level and are shown as fold of change relative to the mRNA level in the negative control group. Values are mean ± SD. Lane 1: Negative control group, Lane 2: Positive control group, Lane 3: Dietary supplemented group with ultrasonicated *Rosmarinus officinalis* ethanolic extract at 100 mg/kg, Lane 4: Dietary supplemented group with chitosan nanoparticles at 20 mg/kg, Lane 5: Dietary supplemented group with ultrasonicated *Rosmarinus officinalis* ethanolic extract-chitosan loaded nanoparticles at 20 mg/kg, Lane 6: Dietary prophylactic group with ultrasonicated *Rosmarinus officinalis* ethanolic extract at 100 mg/kg diet, Lane 7: Dietary prophylactic group with chitosan nanoparticles at 20 mg/kg diet, Lane 8: Dietary prophylactic group with ultrasonicated *Rosmarinus officinalis* ethanolic extract-chitosan loaded nanoparticles at 20 mg/kg diet, –ve: Negative PCR reaction

**Fig. 29:**
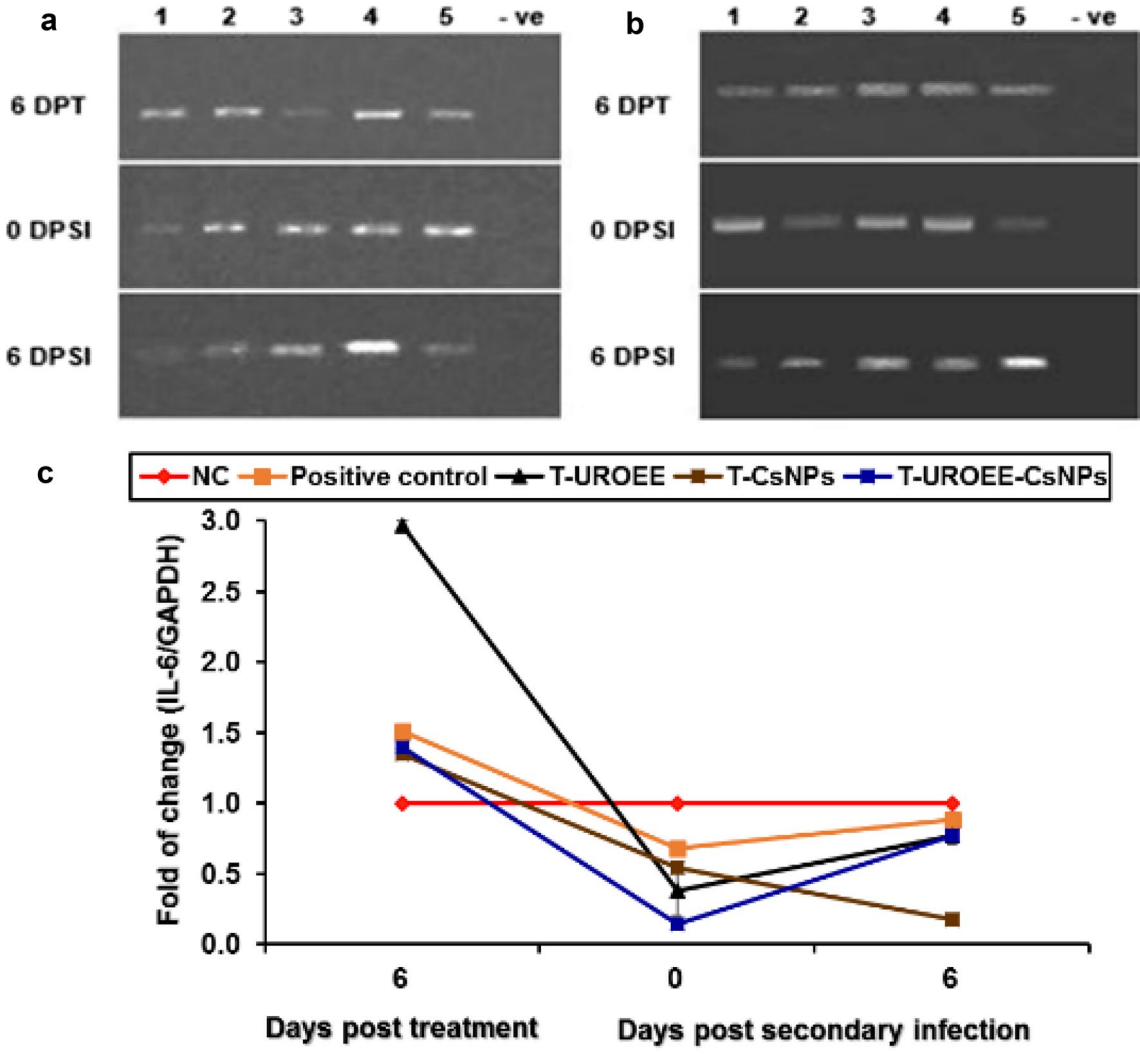
Agarose gel electrophoresis of semi-quantitative RT-PCR for chicken **a** GAPDH and **b** IL-6 in the cecum of the therapeutic treatment groups at 6 days post treatment as well as at 0 and 6 days post-secondary infection with *E. tenella*. **c** Semi-quantitative RT-PCR analysis of chicken IL-6/GAPDH in the cecum. The expression values were obtained by semi-quantitative RT-PCR analysis were normalized to the GAPDH mRNA level and are shown as fold of change relative to the mRNA level in the negative control group. Values are mean ± SD. Lane 1: Negative control group, Lane 2: Positive control group, Lane 3: Therapeutic treatment group with ultrasonicated *Rosmarinus officinalis* ethanolic extract at 100 mg/kg B.W., Lane 4: Therapeutic treatment group with chitosan nanoparticles at 20 mg/kg B.W., Lane 5: Therapeutic treatment group with ultrasonicated *Rosmarinus officinalis* ethanolic extract-chitosan loaded nanoparticles at 20 mg/kg B.W., –ve: Negative PCR reaction

Gene expression of the tested pro-inflammatory cytokines including IFN-γ, IL-1β and IL-6 in the cecum of negative control (NC; G1), positive control (G2), dietary supplemented groups (S-UROEE; G3, S-CsNPs; G4 and S-UROEE-CsNPs; G5), dietary prophylactic groups (P-UROEE; G6, P-CsNPs; G7 and P-UROEE-CsNPs; G8), and therapeutic treatment groups (T-UROEE; G9, T-CsNPs; G10 and T-UROEE-CsNPs; G11) are indicated in Figs. (24, 25, 26, 27, 28 and 29) and in supplementary material (Tables S1 & S2).

The data concerned IFN-γ mRNA expression are clarified in Figs. 24 and 25. This data demonstrated a significant upregulation (*P* < 0.05) in IFN-γ mRNA expression in *E. tenella* infected positive control group at 6 DPPI (~ 0.65-fold), while its expression was significantly (*P* < 0.05) downregulated by ~ 0.38-fold and 0.27-fold at 0 and 6 DPSI, respectively in comparison to NC group. With respect to the supplemented groups, a significant downregulation was recorded in IFN-γ mRNA expression in chickens of S-UROEE, S-CsNPs and S-UROEE-CsNPs groups after primary and secondary infection in relative to NC group; the expression was downregulated (*P* < 0.05) by ~ 0.82, 0.55, 0.64-fold at 6 DPPI as well as by ~ 0.45, 0.47 and 0.55-fold at 0 DPSI and by ~ 0.60, 0.63 and 0.64-fold at 6 DPSI in S-UROEE, S-CsNPs and S-UROEE-CsNPs groups, respectively (Fig. 24). Compared to the positive control group, the mRNA expression levels of IFN-γ of the dietary prophylactic groups (P-UROEE, P-CsNPs and P-UROEE-CsNPs) were significantly (*P* < 0.05) downregulated by ~ 0.17, 0.32 and 0.21-fold, respectively at 6 DPPI. However, its expression level was significantly downregulated (*P* < 0.05) by ~ 0.20, 0.34 and 0.40-fold at 0 DPSI and by ~ 0.40, 0.49 and 0.60-fold at 6 DPSI in P-UROEE, P-CsNPs and P-UROEE-CsNPs groups, respectively, in comparison to a positive control group (Fig. 24). In the therapeutic treated group with T-UROEE, IFN-γ mRNA expression was significantly (*P* < 0.05) upregulated by ~ 1.55-fold, while T-CsNPs and T-UROEE-CsNPs groups exhibited significant (*P* < 0.05) downregulation in IFN- γ mRNA expression by 2.98 and 2.10-fold, respectively, when compared to the positive control group that showed significant (*P* < 0.05) upregulation by 3.05-fold at 6 DPT. Moreover, significant downregulation (*P* < 0.05) was observed in the level of IFN-γ mRNA expression in T-UROEE, T-CsNPs and T-UROEE-CsNPs groups by ~ 0.37, 0.27 and 0.08-fold at 0 DPSI and by ~ 0.26, 0.24 and 0.21-fold at 6 DPSI, respectively in comparison to the infected chickens with *E. tenella* (Fig. 25).

The results of IL-1β mRNA expression are also shown in Figs. 26 and 27. The results showed that IL-1β mRNA expression in infected chickens with *E. tenella* (positive control group) was significantly upregulated (*P* < 0.05) at 6 DPPI by ~ 0.10-fold related to the NC group. In addition, its expression was significantly downregulated (*P* < 0.05) by ~ 0.09-fold at 0 DPSI and by ~ 0.34-fold at 6 DPSI compared to the NC group. The supplemented groups with UROEE, CsNPs, UROEE-CsNPs induced significant (*P* < 0.05) downregulation in IL-1β mRNA expression by ~ 0.10, 0.19 and 0.35-fold, respectively at 6 DPPI. Furthermore, IL-1β mRNA expression revealed non-significant changes at 0 DPSI, while it was downregulated with a significant value (*P* < 0.05) in S-UROEE, S-CsNPs and S-UROEE-CsNPs by ~ 0.79, 0.80 and 0.84-fold, respectively at 6 DPSI, when compared to the NC group (Fig. 26). Concerning the dietary prophylactic groups; an observed significant (*P* < 0.05) downregulation (~ 0.6, 0.22 and 0.43-fold), was detected in IL-1β mRNA expression of P-UROEE, P-CsNPs and UROEE-CsNPs groups, respectively at 6 DPPI in comparison to the positive control group. Moreover, an observed downregulation (*P* < 0.05) in its expression was detected in P-UROEE, P-CsNPs and P-UROEE-CsNPs groups by ~ 0.16, 0.37 and 0.56-fold) at 0 DPSI and by ~ 0.31, 0.36 and 0.43-fold at 6 DPSI, respectively in comparison to the positive control group (Fig. 26). With regard to the therapeutic treatment groups; a significant (*P* < 0.05) upregulation (~ 2.27, 0.61 and 1.27-fold) was detected in IL-1β mRNA expression of T-UROEE, T-CsNPs and T-UROEE-CsNPs groups, respectively at 6 DPT, in comparison to the infected group with *E. tenella* that revealed an increase by ~ 0.73-fold. However, a significant (*P* < 0.05) decrease in its expression was observed after secondary infection with *E. tenella* as it was reduced by ~ 0.46, 0.43 and 0.44-fold at 0 DPSI and by ~ 0.27, 0.53 and 0.08-fold at 6 DPSI in T-UROEE, T-CsNPs and T-UROEE-CsNPs groups, respectively compared to the infected chickens with *E. tenella* (positive control group) (Fig. 27).

The results concern IL-6 mRNA gene expression as shown in Figs. 28 and 29. The positive control group showed significant (*P* < 0.05) increases in IL-6 mRNA gene expression at 6 DPPI by ~ 0.32-fold, while its expression was downregulated with a significant value (*P* < 0.05) at 0 DPSI (~ 0.32-fold) and 6 DPSI (~ 0.12-fold) as relative to the NC group (Fig. 28). The dietary supplemented groups; S-UROEE, S-CsNPs and U-EEERO-CsNPs showed significant (*P* < 0.05) reduction in IL-6 gene expression at 6 DPPI (~ 0.62, 0.23 and 0.12-fold, respectively) in comparison to the NC group. Moreover, the three groups displayed significant (*P* < 0.05) downregulation following secondary infection at 0 DPSI (~ 0.43, 0.27 and 0.71-fold, respectively) and 6 DPSI (~ 0.85, 0.71 and 0.59-fold, respectively) (Fig. 28). For the three dietary prophylactic groups; P-UROEE, P-CsNPs and P-UROEE-CsNPs, a remarkable significant (*P* < 0.05) downregulation in IL-6 mRNA expression was exhibited by ~ 0.63, 0.75 and 0.62-fold, respectively at 6 DPPI, when compared to the positive control group. However, its expression was downregulated with a significant value (*P* < 0.05) following the secondary infection at 0 DPSI (~ 0.18, 0.35 and 0.17-fold, respectively) and 6 DPSI (~ 0.25, 0.25 and 0.10-fold, respectively) relative to the positive control group. On the other hand, an observable significant (*P* < 0.05) upregulation was documented in T-UROEE group (1.45-fold), while significant (*P* < 0.05) downregulation in IL-6 mRNA expression (~ 0.16 and 0.12-fold, respectively) was recorded in the T-CsNPs and T-UROEE-CsNPs groups, respectively at 6 DPT. Moreover, following the secondary infection, T-UROEE, T-CsNPs and T-UROEE-CsNPs groups revealed significant (*P* < 0.05) downregulation in IL-6 expression by ~ 0.30, 0.14 and 0.53-fold, respectively, at 0 DPSI and by ~ 0.11, 0.70 and 0.11-fold, respectively at 6 DPSI, when compared to the positive control group (Fig. 29).

### Gene Expression of Anti-inflammatory Cytokines in Cecum of *E. tenella* Infected Chickens

**Fig. 30:**
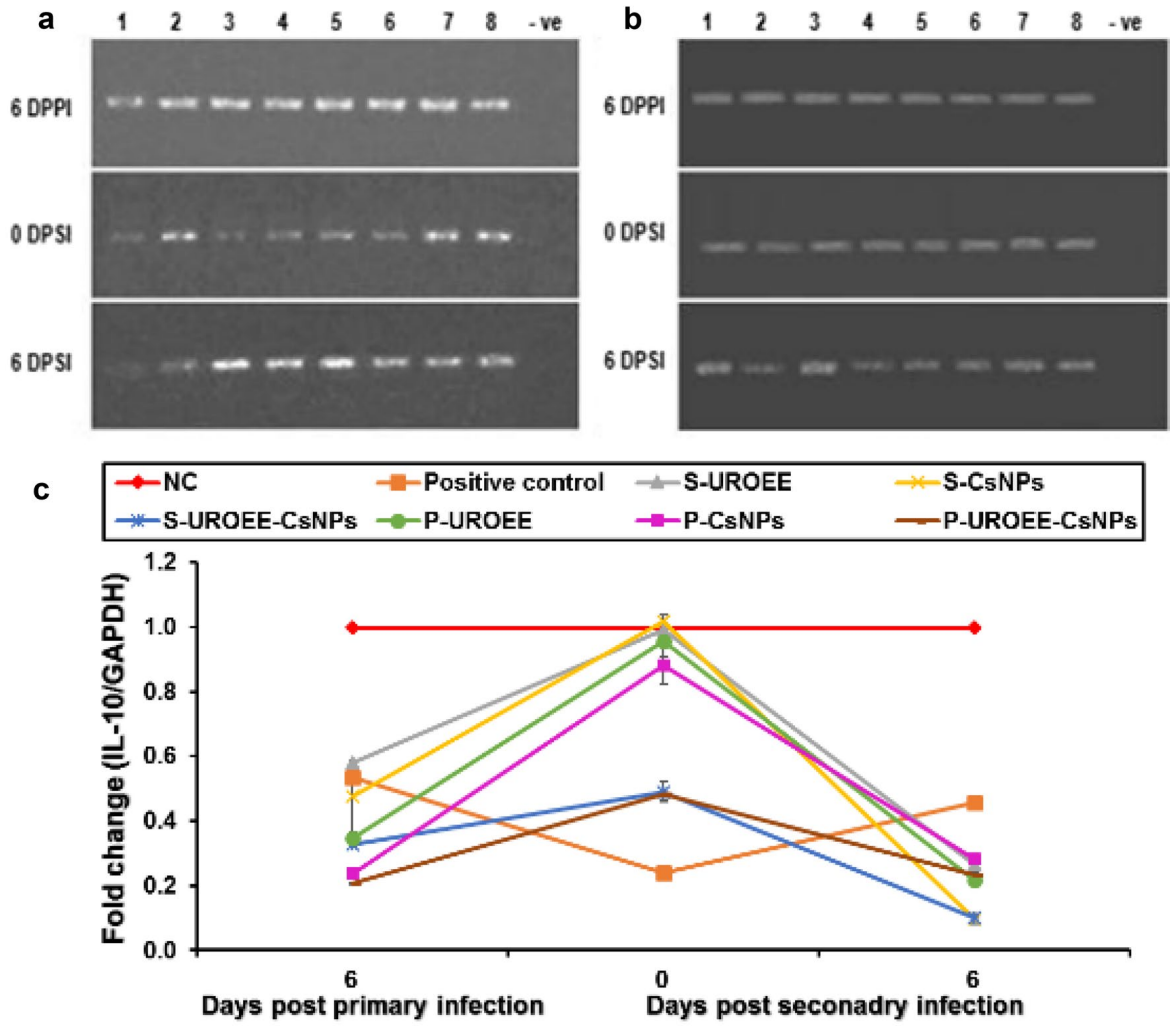
Agarose gel electrophoresis of semi-quantitative RT-PCR for chicken **a** GAPDH and **b** IL-10 in the cecum of the dietary prophylactic groups at 6 days post-primary infection as well as at 0 and 6 days post-secondary infection with *E. tenella*. **c** Semi-quantitative RT-PCR analysis of chicken IL-10/GAPDH in the cecum. The expression values were obtained by semi-quantitative RT-PCR analysis were normalized to the GAPDH mRNA level and are shown as fold of change relative to the mRNA level in the negative control group. Values are mean ± SD. Lane 1: Negative control group, Lane 2: Positive control group, Lane 3: Dietary supplemented group with ultrasonicated *Rosmarinus officinalis* ethanolic extract at 100 mg/kg, Lane 4: Dietary supplemented group with chitosan nanoparticles at 20 mg/kg, Lane 5: Dietary supplemented group with ultrasonicated *Rosmarinus officinalis* ethanolic extract-chitosan loaded nanoparticles at 20 mg/kg, Lane 6: Dietary prophylactic group with ultrasonicated *Rosmarinus officinalis* ethanolic extract at 100 mg/kg diet, Lane 7: Dietary prophylactic group with chitosan nanoparticles at 20 mg/kg diet, Lane 8: Dietary prophylactic group with ultrasonicated *Rosmarinus officinalis* ethanolic extract-chitosan loaded nanoparticles at 20 mg/kg diet, –ve: Negative PCR reaction

**Fig. 31:**
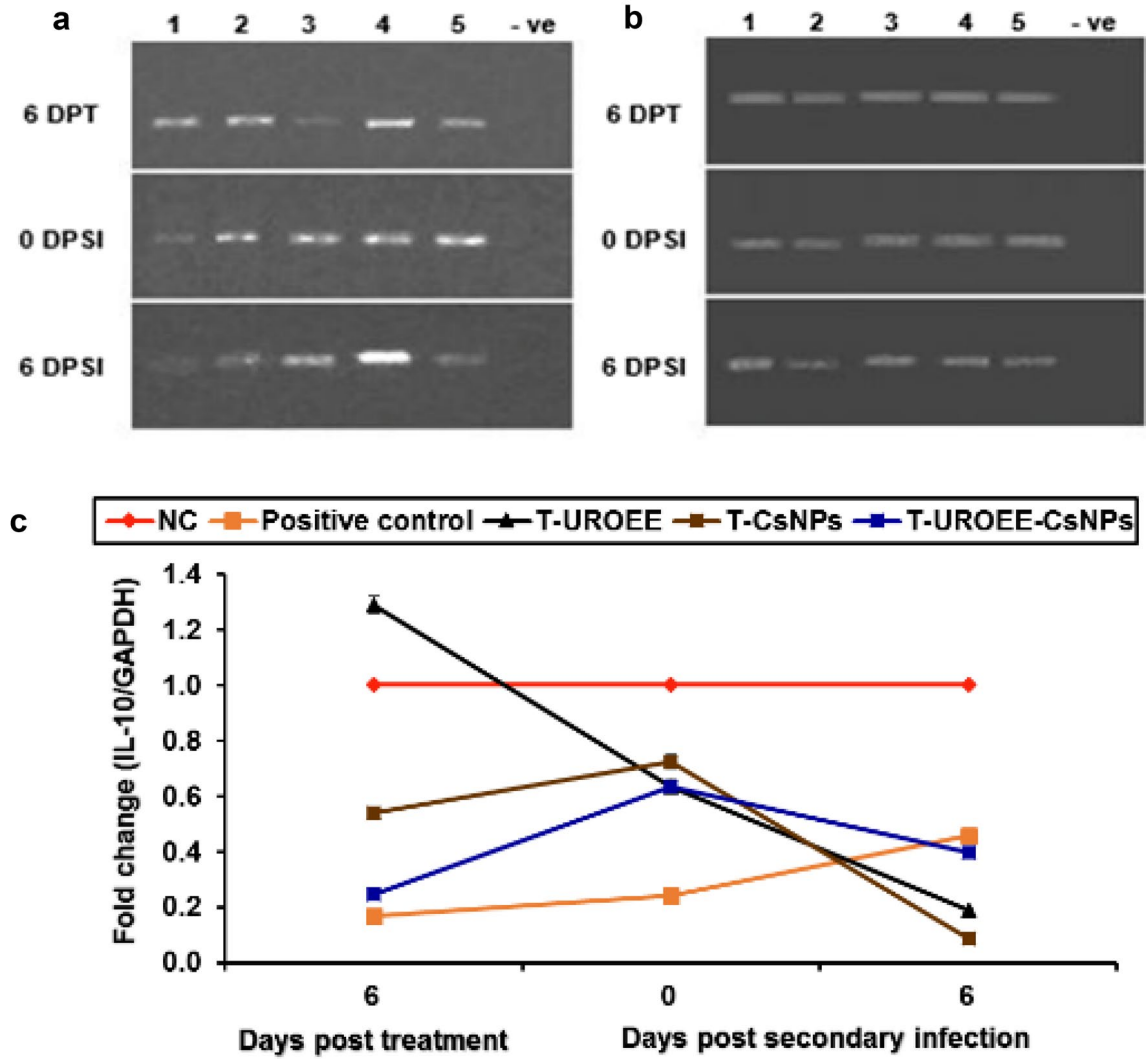
Agarose gel electrophoresis of semi-quantitative RT-PCR for chicken **a** GAPDH and **b** IL-10 in the cecum of the therapeutic treatment groups at 6 days post treatment as well as at 0 and 6 days post-secondary infection with *E. tenella*. **c** Semi-quantitative RT-PCR analysis of chicken IL-10/GAPDH in the cecum. The expression values were obtained by semi-quantitative RT-PCR analysis were normalized to the GAPDH mRNA level and are shown as fold of change relative to the mRNA level in the negative control group. Values are mean ± SD. Lane 1: Negative control group, Lane 2: Positive control group, Lane 3: Therapeutic treatment group with ultrasonicated *Rosmarinus officinalis* ethanolic extract at 100 mg/kg B.W., Lane 4: Therapeutic treatment group with chitosan nanoparticles at 20 mg/kg B.W., Lane 5: Therapeutic treatment group with ultrasonicated *Rosmarinus officinalis* ethanolic extract-chitosan loaded nanoparticles at 20 mg/kg B.W., –ve: Negative PCR reaction

**Fig. 32:**
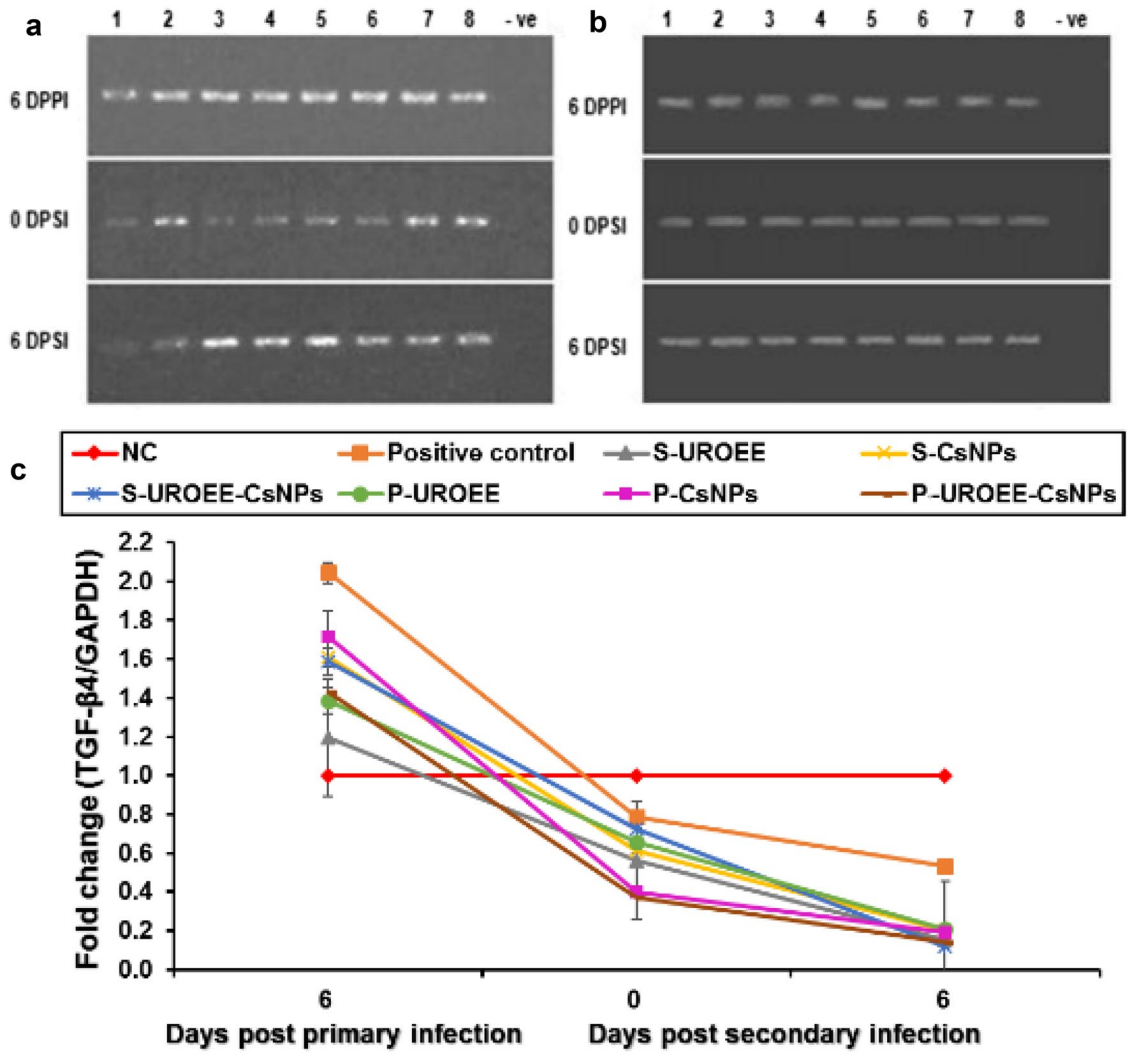
Agarose gel electrophoresis of semi-quantitative RT-PCR for chicken **a** GAPDH and **b** TGF-β4 in the cecum of the dietary prophylactic groups at 6 days post-primary infection as well as at 0 and 6 days post-secondary infection with *E. tenella*. **c** Semi-quantitative RT-PCR analysis of chicken TGF-β4/GAPDH in the cecum. The expression values were obtained by semi-quantitative RT-PCR analysis were normalized to the GAPDH mRNA level and are shown as fold of change relative to the mRNA level in the negative control group. Values are mean ± SD. Lane 1: Negative control group, Lane 2: Positive control group, Lane 3: Dietary supplemented group with ultrasonicated *Rosmarinus officinalis* ethanolic extract at 100 mg/kg, Lane 4: Dietary supplemented group with chitosan nanoparticles at 20 mg/kg, Lane 5: Dietary supplemented group with ultrasonicated *Rosmarinus officinalis* ethanolic extract-chitosan loaded nanoparticles at 20 mg/kg, Lane 6: Dietary prophylactic group with ultrasonicated *Rosmarinus officinalis* ethanolic extract at 100 mg/kg diet, Lane 7: Dietary prophylactic group with chitosan nanoparticles at 20 mg/kg diet, Lane 8: Dietary prophylactic group with ultrasonicated *Rosmarinus officinalis* ethanolic extract-chitosan loaded nanoparticles at 20 mg/kg diet, –ve: Negative PCR reaction

**Fig. 33:**
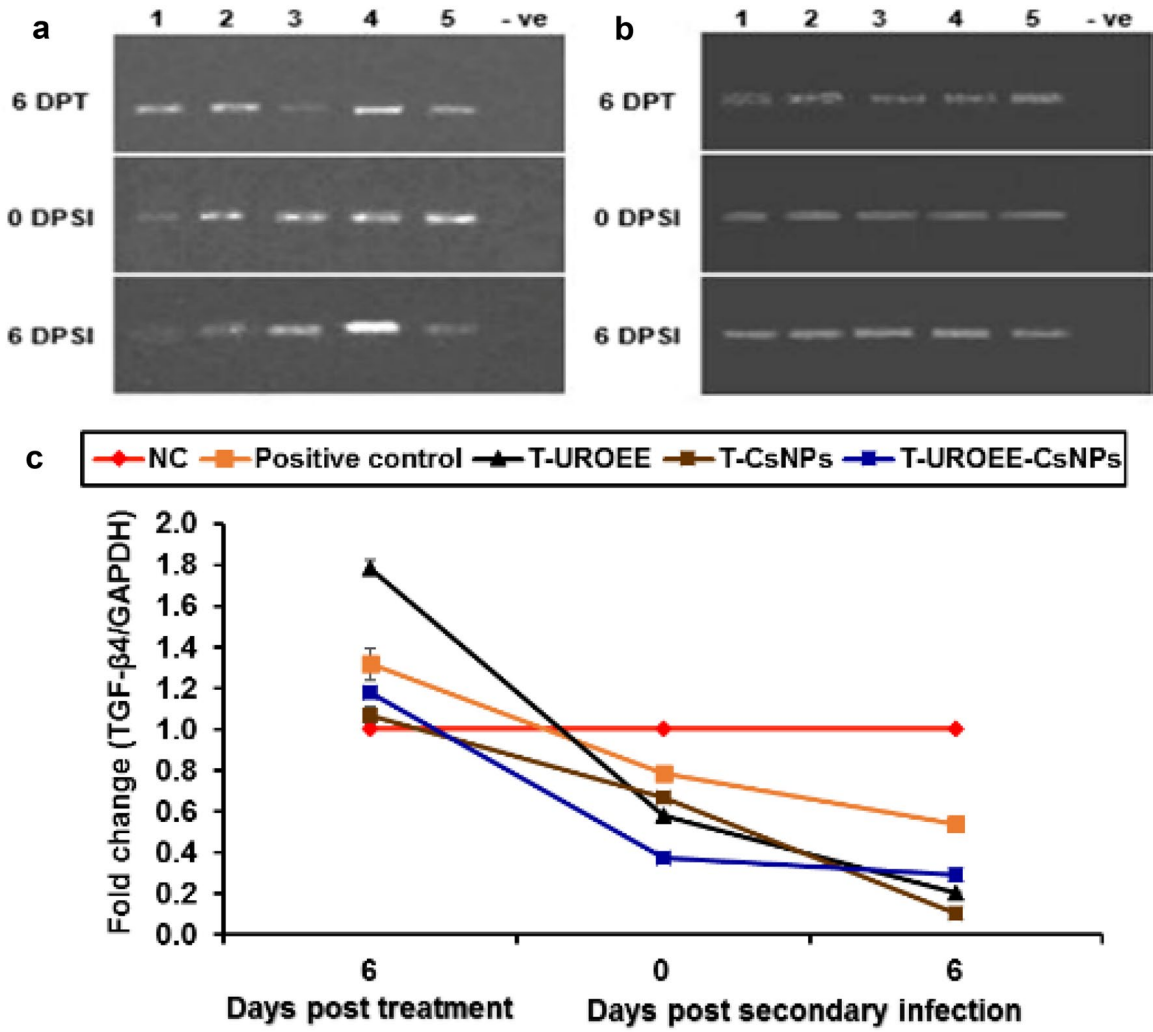
Agarose gel electrophoresis of semi-quantitative RT-PCR for chicken** a** GAPDH and **b** TGF-β4 in the cecum of the therapeutic treatment groups at 6 days post treatment as well as at 0 and 6 days post-secondary infection with *E. tenella*. **c** Semi-quantitative RT-PCR analysis of chicken TGF-β4/GAPDH in the cecum. The expression values were obtained by semi-quantitative RT-PCR analysis were normalized to the GAPDH mRNA level and are shown as fold of change relative to the mRNA level in the negative control group. Values are mean ± SD. Lane 1: Negative control group, Lane 2: Positive control group, Lane 3: Therapeutic treatment group with ultrasonicated *Rosmarinus officinalis* ethanolic extract at 100 mg/kg B.W., Lane 4: Therapeutic treatment group with chitosan nanoparticles at 20 mg/kg B.W., Lane 5: Therapeutic treatment group with ultrasonicated *Rosmarinus officinalis* ethanolic extract-chitosan loaded nanoparticles at 20 mg/kg B.W., –ve: Negative PCR reaction

Gene expression of the tested anti-inflammatory cytokines including IL-10 and TGF-β4 in the cecum of negative control (NC; G1), positive control (G2), dietary supplemented groups (S-UROEE; G3, S-CsNPs; G4 and S-UROEE-CsNPs; G5), dietary prophylactic groups (P-UROEE; G6, P-CsNPs; G7 and P-UROEE-CsNPs; G8), and therapeutic treatment groups (T-UROEE; G9, T-CsNPs; G10 and T-UROEE-CsNPs; G11) are shown in Figs. (30, 31, 32 and 33) and in supplementary material (Tables S3 and S4).

The levels of expressed mRNA for IL-10 in the cecum of infected chickens of *E. tenella* (positive control group) were significantly (*P* < 0.05) downregulated following primary (~ 0.47 at 6 DPPI) and secondary infection (~ 0.76 and 0.54-fold at 0 and 6 DPSI, respectively) when comparing to the non-infected chickens (NC group) (Fig. 30). In the three dietary supplemented groups with UROEE, CsNPs and UROEE-CsNPs, the mRNA expression level of IL-10 was significantly (*P* < 0.05) decreased following primary infection by (~ 0.42, 0.52 and 0.67-fold, respectively) at 6 DPPI, when compared to NC group. In addition, the expression of this anti-inflammatory cytokine was non-changed (*P* > 0.05) in both S-UROEE, S-CsNPs groups and downregulated (*P* < 0.05) in S-UROEE-CsNPs group (~ 0.51-fold) at 0 DPSI, while significant downregulation was documented at 6 DPSI in the three supplemented groups; S-UROEE, S-CsNPs and S-UROEE-CsNPs (~ 0.74, 0.90 and 0.90-fold, respectively) in relation to the NC group (Fig. 30). With respect to the dietary prophylactic groups; when comparing them with the positive control group, it was found that mRNA expression level of IL-10 was significantly (*P* < 0.05) downregulated by ~ 0.18, 0.29 and 0.32-fold at 6 DPPI in P-UROEE, P-CsNPs and P-UROEE-CsNPs groups, respectively. On the other hand, the three dietary prophylactic groups showed a significant (*P* < 0.05) increase in the mRNA expression level of IL-10 by ~ 0.72, 0.64 and 0.24-fold at 0 DPSI and then downregulated at 6 DPSI (~ 0.24, 0.18 and 0.23-fold) in the three groups, respectively, when compared to the positive control group (Fig. 30). In the therapeutic treatment groups (T-UROEE, T-CsNPs and T-UROEE-CsNPs), IL-10 mRNA expression showed significant (*P* < 0.05) upregulation (~ 1.12, 0.37, 0.25-fold) in T-UROEE, T-CsNPs and T-UROEE-CsNPs groups, respectively at 6 DPT, compared to the positive control that showed significant downregulation by 0.83-fold. Otherwise, the mRNA expression level of IL-10 was significantly (*P* < 0.05) upregulated in the three therapeutic groups; T-UROEE, T-CsNPs and T-UROEE-CsNPs by ~ 0.39, 0.49 and 0.39-fold, respectively at 0 DPSI, while, at 6 DPSI; its expression was downregulated (~ 0.27, 0.37 and 0.06-fold) with a significant value (*P* < 0.05) in the three therapeutic groups, respectively relative to the positive control group (Fig. 31).

Comparing the positive control group with the NC group, the mRNA expression of TGF-β4 was significantly upregulated (*P* < 0.05) at 6 DPPI (~ 1.04-fold). Otherwise, its expression was significantly (*P* < 0.05) reduced at 0 and 6 DPSI by ~ 0.22 and 0.46-fold, respectively relative to the NC group (Fig. 32). A significant (*P* < 0.05) upregulation in TGF-β4 expression level was recorded in the three dietary supplemented groups; S-UROEE, S-CsNPs and S-UROEE-CsNPs at 6 DPPI (~ 0.19, 0.61 and 0.59-fold, respectively). Despite this, its expression was downregulated with a significant value (*P* < 0.05) after secondary infection by ~ 0.44, 0.39 and 0.27-fold at 0 DPSI as well as by ~ 0.85, 0.79 and 0.88-fold, respectively at 6 DPSI, respectively when compared to NC group (Fig. 32). Regarding the dietary prophylactic groups, significant decreases (*P* < 0.05) in TGF-β4 mRNA expression level was documented by ~ 0.66, 0.32 and 0.61-fold in P-UROEE, P-CsNPs and P-UROEE-CsNPs groups, respectively at 6 DPPI. However, its expression level was also significantly (*P* < 0.05) decreased by ~ 0.12, 0.38 and 0.41-fold at 0 DPSI and by ~ 0.33, 0.35 and 0.40-fold in P-UROEE, P-CsNPs and P-UROEE-CsNPs groups, when compared to the positive control group (Fig. 32). Comparing the therapeutic treated groups with UROEE, CsNPs and UROEE-CsNPs to the infected group with *E. tenella* (positive control group), it was observed that there were significant (*P* < 0.05) increase in TGF-β4 expression of T-UROEE group by ~ 0.46-fold and significant (*P* < 0.05) decreases in TGF-β4 expression of CsNPs and UROEE-CsNPs by ~ 0.26 and 0.20-fold, respectively at 6 DPT. In addition, its expression was significantly (*P* < 0.05) downregulated after secondary infection at 0 DPSI (~ 0.20, 0.11 and 0.41-fold) and 6 DPSI (~ 0.34, 0.44 and 0.25-fold) in the three therapeutic groups; T-UROEE, T-CsNPs and T-UROEE-CsNPs, respectively in comparison to the positive control group (Fig. 33).

## Discussion

Accurate identification of *Eimeria* spp. is important not only for the diagnosis of disease but also for management of subclinical infection, development and application of effective control strategies, as well as biological and epidemiological study [45]. The emergence of the polymerase chain reaction (PCR) allows the precise identification of the seven species of *Eimeria* spp. [46, 47]. The amplification of regions of the internal transcribed spacer-1 (ITS-1) of the ribosomal DNA is now a widely available technique to discriminate between all *Eimeria* spp. using PCR molecular techniques [48, 49]. The ITS is a piece of non-functional RNA located between structural ribosomal RNAs (rRNA) on a common precursor transcript. This region of the genome contains several segments used for *Eimeria* spp. identification, including the 5′ external transcribed sequence (5′ ETS), 18S rRNA, ITS-1, 5.8S rRNA, ITS-2, 26S rRNA, and finally the 3′ ETS [50]. The identification of *Eimeria* species in the present study was based on a molecular PCR assay that revealed the presence of amplicons with 278 bp on 1% agarose gel using species-specific primers targeting ITS-1 region for *E. tenella*. The same amplicon size for *E. tenella* was confirmed by [38, 39].

Coccidiosis is a protozoan infection, caused by coccidia that has a negative impact on the health and performance of most vertebrates (*Eimeria* spp. in birds and livestock or *Isospora* sp in cats and dogs) [51]. In the present study, the typical symptoms of coccidiosis appeared only in the infected broiler chickens with *E. tenella* (positive control group) but not in the treatment groups and these symptoms include depression, ruffled feathers, reduction of food intake, weight loss, emaciation and decreased activity, accompanied with progressive weakness. Similar findings were reported by [52] who indicated that infected chickens with *E. tenella* suffered from signs of coccidiosis, such as crouching, loss of appetite, depression, and ruffled feathers and these symptoms not appear in the treated groups with *Cinnamomum verum* bark and *Rumex nervosus* leaves.

Coccidiosis is an extremely common infectious disease that destroys the intestinal epithelial cells of birds and causes terrible bloody diarrhea [53]. In this study, five DPI, extensive bloody diarrheal feces were observed in the positive control and the therapeutic treatment groups, while mild bloody diarrhea was seen in the three prophylactic groups. Therefore, the decreased hemorrhagic diarrhea may protect infected birds from secondary infections, inflammatory responses, and toxic substances ingestion [54].

Fecal oocyst count was used to investigate the susceptibility of chickens to coccidiosis [55]. The spread of oocysts in feces is a risk factor for the spread of coccidiosis in intensive farming [56]. In the present study, the loading of UROEE on CsNPs significantly reduced the oocysts output/ gram feces, followed by CsNPs and then UROEE groups, when compared to the positive control group. Similarly, chitosan exhibited anti-*Eimeria* activity as it reduced the number of fecal oocyst output in infected mice with *E. papillata*; this stands for that chitosan treatment impairs intracellular development and replication of *E. papillata* in the jejunal epithelium of mice [57]. The excretion of oocyst shedding was decreased in the treated group with *Ferulago angulata* hydroalcoholic extract, compared to the positive control group in infected broilers with mixed *Eimeria* spp. [58]. The use of crude methanol extract of *Garcinia kola* significantly reduced oocysts excretion in infected chickens with *E. tenella* [54]. However, the most reduced number of oocysts output was accountable for the prophylactic treatment groups suggesting that UROEE and its chitosan-loaded nanoparticles could play an important role in controlling large-scale avian coccidiosis outbreaks in poultry farms.

Histopathological examination is still one of the most common methods for determining the extent of tissue degeneration and cyto-architectural changes [59]. The chicken intestine has a very diverse microbiota, which interacts with the host and forms a stable and coordinated intestinal system [60]. The cecal microbiota affects the health and productivity of chickens by regulating nutrient absorption and metabolism, immune activity and function, and pathogen exclusion [60, 61]. In the present study, microscopic observations of the cecal tissue of infected broiler chickens with *E. tenella* appeared damaged with severe inflammation, desquamation of superficial epithelium villous atrophy and the structure was vague, when compared to the non-infected chickens. Furthermore, the mucosa and Lieberkühn’s crypts epithelium lining appeared also infected with a remarkable enormous amount of different developmental endogenous parasitic stages including oocysts, schizonts and merozoites occupying the sites of absorptive epithelium (this destructive effect was noticed after primary infection and also continued following the secondary infection with *E. tenella*. In chickens, *E. tenella* specifically targets the ceca, causing damage to the cecal mucosal barrier and therefore causing intestinal microecological disturbance [4]. That penetration of *E. tenella* damaged the cecum villus, sloughed off cecal intestinal epithelial cells, and increased the thickness of the muscularis and serosa significantly [62]. Moreover, this study showed that the use of UROEE, CsNPs and UROEE-CsNPs against infected chickens with *E. tenella* induced improvements in cecal tissue architecture as the shape of villi, glands and lamina propria reobtained their normal structure with decreased number of intracellular parasitic stages and enormous vacuolated schizonts were also seen after the primary and secondary infection. This result is the same with [57] who reported that chitosan treatment impairs intracellular development and replication of *E. papillata* in the jejunal epithelium of mice. In addition, [63] indicated that herbal plants such as *Plectranthus spp* has been shown to repair some lesion and decreased some destruction in the cecum tissue of broiler chickens experimentally infected with *E. tenella* oocysts. A recent study by [64] demonstrated that garlic alcoholic extract has a positive effect on cecal tissue by reducing cecal epithelial necrosis, coccidial stages, and mononuclear cells in the lamina propria. Herbal anticoccidial supplements have therapeutic properties due to the presence of bioactive compounds that improve immunity and antioxidant status, reduce intestinal inflammation, modulate gut microflora, and reduce parasitosis [65]. Flavonoids are well known for their antioxidant effect due to their redox properties. Some flavonoids act on host-parasite interactions, and others disturb the development or metabolism of protozoan parasites [66]. However, the positive involvement of rosemary bioactivity is also reported due to its richness in flavonoids constituents [67]. This could likely be the explanation regarding oocyst output decrease, and the reduction in histopathological lesion findings of this study. Chitosan supplementation improved the intestinal absorptive capacity through increased intestinal villi length [68, 69].

T-cell-mediated immunity by intestinal intraepithelial lymphocytes represents the main component of protective immunity to *Eimeria* infections. This includes the T-helper (CD4^+^) and T-cytotoxic (CD8^+^) cells [5]. T cells play the most important role in response to primary or challenge coccidia infection [70]. In the herein study, IHC semi-quantitative analysis of cecal tissue revealed remarkable significant increases in CD4^+^ and CD8^+^ T lymphocytes protein expression in infected chickens with *E. tenella* that associated with severe coccidian infestation following primary infection more than that after secondary infection. However, a significant decrease in CD4^+^ and CD8^+^ T cells protein expression was demonstrated in either the prophylactic or therapeutic treatment groups with UROEE, CsNPs or UROEE-CsNPs, when compared to the positive control group. Previous studies have shown that following a primary *Eimeria* infection, CD4^+^ and CD8^+^ T lymphocytes increase shortly after primary infection, but decrease during later infection in the small intestine [71]. CD4^+^ T cells are effectors of *Eimeria* resistance during early coccidian infection and could be a source of early IFN-γ [72]. Similar results indicated that higher numbers of CD4^+^ lamina propria lymphocytes were detected within 24 h of intra-cecal inoculation of *E. tenella* sporozoites [73] and increased CD4^+^ IEL were detected in the duodenum during early *E. acervulina* infection [74]. CD8^+^ T cells are important due to their ability to fight intracellular pathogens, such as coccidia [75]. CD8^+^ T cells have been suggested to play a role in the transport of the sporozoites [76]. When oocysts are accidentally ingested, the sporozoites are liberated and migrate to their specific invasion site in the intestine as the cecum in the case of *E. tenella*. CD8^+^ T cells migrating to the cecal tonsils in chickens infected with *E. tenella*, could account for the higher concentrations during early infection [75].

Cytokines are important molecules that control the host immune response to *Eimeria* infection in poultry [77]. The present study demonstrated a significant upregulation in mRNA expression levels of the pro-inflammatory cytokines including IFN-γ, IL-1β and IL-6 in *E. tenella* infected chickens following primary infection, while their expression was significantly downregulated following secondary infection in comparison to the NC group. During primary infection, a pathogen spreads too rapidly, does too much damage and proliferates to a level sufficient to elicit an adaptive immune response and then stimulates the production of antibodies and effector T cells that in turn produce high levels of cytokines to eliminate the pathogen from the body, while in a secondary infection to the same antigen, the antibody and memory T cells remaining in an immunized individual prevent the activation of naive B and T cells but memory cells are rapidly activated and can respond much more quickly and effective than the primary response [78]. The herein results are in agreement with [79] who revealed that the transcripts of the pro-inflammatory cytokines IFN- γ, IL-1β and IL-6 were increased following primary infection, while their expression was slightly changed following secondary infection in *E. tenella*-infected chickens. The *E. tenella* infection caused upregulation in the pro-inflammatory cytokines; IFN- γ, IL-1β and IL-6 in chickens caecal tissue [80]. Also, [81] indicated that IFN-γ transcript levels were shown to be upregulated in the cecum, tonsils, spleen, and intestinal intraepithelial lymphocytes during the course of *E. tenella* infection. When chickens were infected with *E. tenella* oocysts, IFN-γ and IL-1β expression was increased in the cecum at 7 DPI, compared to the control value [82]. In addition, There was an increase in mRNA expression of the pro-inflammatory cytokines IFN-γ, IL-1β and IL-6 in the jejunum of *E. papillata*-infected mice at 5 DPI compared to the non-infected ones [83, 84]. The gene expression of IFN-γ in samples of the duodenum and jejunum was significantly increased in challenged birds compared to the unchallenged ones at 7 DPI with mixed *Eimeria* species (*E. tenella, E. acervulina* and *E. maxima*) [85]*.* In a recent study, the jejunal gene transcripts for IFN-γ were increased in infected birds with mixed *Eimeria* species compared to the non-infected ones [86]. IL-6 was usually indicative of the initiation of an acute-phase response and were upregulated following primary infection with *E. maxima* [87]. Infection with *Eimeria* spp. upregulates the expression of pro-inflammatory cytokines that play an important role in the intestinal inflammation that is a feature of the disease [88, 89]. The high production of IFN-γ may contribute to clearance of the infection and the development of immunity to reinfection [1, 82]. IL-1β is a powerful pro-inflammatory cytokine secreted by many different cell types, with stimulated macrophages being the main producer [82]. Meanwhile, it has been proposed that increased expression of IL-6 in chickens may stimulate a population of heterophils, avian equivalents of mammalian neutrophils, with increased capability of responding to and eliminating infectious pathogens [90]. For homeostasis, the pro-inflammatory cytokine IL-6 was upregulated during infection [91]. In the present study, the use of UROEE and its chitosan-loaded nanoparticles as dietary prophylactic agents and therapeutic treatments could ameliorate the inflammation induced by *E. tenella* through the downregulation of pro-inflammatory IFN-γ mRNA expression following primary and secondary infection except T-UROEE group comparing to the positive control group. The present results are consistent with previous reports by [89] who indicated that garlic treatment downregulated the increased IFN-γ expression level induced in *E. papillata*-infected mice at 4 DPI. Chickens given a diet containing *Capsicum* and turmeric oleoresins prior to *E. tenella* infection, had reduced levels of transcripts encoding IFN-γ compared with un-supplemented controls [92]. Also, the herein result agrees with [83] who indicated that berberine attenuates the inflammatory response to *E. papillata* in the mouse jejunum by reducing mRNA expression levels of IFN-γ at 5 DPI. Moreover, pomegranate greatly reduced the mRNAs of the inflammatory cytokine INF-γ compared to the infected mice with *E. papillate* at 5 DPI [93]. The treatment with selenium nanoparticles was effective in ameliorating the upregulation of the IFN-γ gene that was associated with inflammation induced by *E. papillata* at 5 DPI [84]. In the current study, the pro-inflammatory cytokine IL-1β mRNA expression was significantly downregulated following primary and secondary infection with *E. tenella* when using UROEE and its chitosan-loaded nanoparticles as dietary prophylactic agents against *E. tenella* parasitic infection. However, the use of these materials as therapeutic treatments could reduce IL-1β mRNA expression only following the secondary infection. Furthermore, the herein study revealed that the dietary prophylactic additives of UROEE, CsNPs and UROEE-CsNPs as well as the therapeutic treatment with CsNPs and UROEE-CsNPs but not UROEE could reduce the inflammatory response induced by *E. tenella* following primary and secondary infection through the reduction of the pro-inflammatory cytokine IL-6 mRNA expression, when compared to the positive control group. Berberine attenuates the inflammatory response to *E. papillata* in the mouse jejunum by reducing mRNA expression levels of IL-6 and IL-1β at 5 DPI [83]. In addition, pomegranate greatly reduced the mRNAs of the pro-inflammatory cytokine IL-1β compared to the infected mice with *E. papillate* at 5 DPI [93]. The treatment with selenium nanoparticles is effective in ameliorating the upregulation of IL-1β gene that was associated with inflammation induced by *E. papillata* at 5 DPI [84]. Recently, *Rumex nervosus* extract could significantly downregulate the IL-6 and IL-1β gene expression in the cecal tissue of infected chickens with *E. tenella* [80].

IL-10 is an anti-inflammatory cytokine that inhibits the function of macrophages and dendritic cells, including their production of pro-inflammatory cytokines [94]. In the current study, the levels of expressed mRNA for the anti-inflammatory IL-10 in the cecum were significantly downregulated following primary and secondary infection with *E. tenella* when compared to the non-infected chickens. This result is consistent with previous results demonstrating that the levels of transcripts encoding IL-10 in intestinal intraepithelial lymphocytes were decreased in parasite-infected broiler chickens with *E. acervulina* [95]. Another study by [57] demonstrated the decreased mice IL-10 expression following *E. papillata* infection. In addition, the cecum of infected chickens with *E. tenella* had decreased IL-10 expression [96]. IL-10 inhibits the synthesis of pro-inflammatory cytokines (including IL-1β, and IL-6), thus down-regulating inflammatory Th1 responses [97, 98]. Protozoan parasites can use IL-10 to downregulate host immunity and reduce pathogen-damaging inflammatory responses [99]. In the present study, the three dietary-supplemented groups with UROEE, CsNPs and UROEE-CsNPs showed that the mRNA expression level of IL-10 was significantly decreased following primary and secondary infection, when compared to NC group. The levels of transcripts for the anti-inflammatory IL-10 cytokine increased in uninfected anethole-fed birds compared with controls [100]. In the current research, when comparing the dietary prophylactic and therapeutic treatment groups with the positive control group, it was found that mRNA expression level of IL-10 was significantly upregulated following primary infection then downregulated following secondary infection with *E. tenella*. The transcript level for IL-10 was increased in *E. acervulina* infected, anethole-treated birds compared with untreated controls [100]. The levels of expressed mRNA for IL-10 were increased in the chitosan treatment group compared to the infected group with *E. papillata* at 5 DPI [57]. IL-10 has been suggested as a master regulator, acting through a negative feedback mechanism to prevent the overexpression of Th1 and Th2 immune responses [101]. In the immune system, TGF-β plays an important role in the development of T lymphocytes and has important anti-inflammatory activity [102]. TGF-β4 has been found to be important in regulating immune function in coccidia-infected IECs of chicken [103]. Comparing the infected group with *E. tenella* to the non-infected one in this study, the mRNA expression of TGF-β4 was significantly upregulated following primary infection. Otherwise, its expression was significantly reduced following secondary infection relative to the NC group. TGF-β4, in particular, has been found to increase in chickens after coccidian infection [104]. Following primary *E. tenella* infection, the level of TGF-β4 expression was increased, while it was slightly changed following secondary infection [79]. The expression of TGF-β4 was increased in coccidian-infected chickens with *E. acervuline* [105]*.* This result is also consistent with that reported by [106], who found that TGF-β4 expression was mainly enhanced at the late phase of infection. Moreover, the level of expressed mRNA for TGF-β was increased in infected mice with *E. papillata* at 5 DPI compared to the non-infected [57]. TGF-β4 expression was increased at 8 DPI in the spleens of birds challenged with *E. tenalla* [107]. The production of TGF-β has been found to be combined with the production of both IFN-γ and TNF-α following *Eimeria* infection [108]. The secretion of TGF-β in the mouse intestine has been found to be protective against *Eimeria* infection [109]. The present study also indicated that the dietary prophylactic groups exhibited significant decreases in TGF-β4 mRNA expression level following primary infection. However, its expression level was significantly decreased following the secondary infection, relative to the positive control group. The expression of TGF-β was decreased at 5 DPI after chitosan treatment in comparison to the non-treated infected mice with *E. papillate* [57]. TGF-β4 has been found to be important in regulating immune function in coccidia-infected intestinal intraepithelial lymphocytes of chicken [103]. Chitosan exhibited anti-*Eimeria* activity, through decreasing mRNA expression of TGF-β in the mouse jejunum infected with *E. papillata*, perhaps due to its anti-inflammatory and antioxidant activity [110]. The plant extract and essential oil mixtures could inhibit *Eimeria tenella* invasion both in vitro and in vivo, demonstrating their potential as anticoccidial agents [111]. Essential oil-based preparations have been marketed as an alternative natural anticoccidial, as they have multiple biological properties that directly inhibit *Eimeria* spp. They also upregulate the host immune response, improve the antioxidant capacity, and exhibit antimicrobial activity upon ingestion [3].

## Conclusion

Collectively, *R. officinalis* ethanolic extract and its chitosan-loaded nanoparticles is a promising natural prophylactic agent with anticoccidial, protective immunomodulatory and anti-inflammatory properties that can be used as an effectual dietary additive to improve immunological resistance to *E. tenella* experimental infection in broiler chickens and can also be used as a therapeutic treatment to reduce pathological effects of cecal coccidiosis that could be a potential alternative to anticoccidial commercial drugs. However, further studies are required to understand the metabolic activity, the role and mechanism of action of each component during the infection, focusing on joint interaction of feed additive with host gut microbiota towards the control of chicken coccidiosis to provide efficacious treatment measures. Therefore, it is planned to make chromatographic analysis as HPLC for example to identify the role and mechanism of components present in *Rosmarinus officinalis* ethanolic extract and its chitosan-loaded nanoparticles and study the dietary and therapeutic anticoccidial effect and mechanism of each component separately with comparing to some commercial anticoccidial drugs.

### Supplementary Information

Below is the link to the electronic supplementary material.

Supplementary file1 (DOCX 26 KB)

## Data Availability

All data of this study can be provided on request.
