# Supplementary material for: Prophylactic and Therapeutic Efficacy of Ultrasonicated Rosmarinus officinalis Ethanolic Extract and its Chitosan-Loaded Nanoparticles Against Eimeria tenella Infected Broiler Chickens
Source: Acta Parasitol. 2024 Mar 16;69(1):951–99. doi: 10.1007/s11686-024-00793-3 (PMC11001757; doi:10.1007/s11686-024-00793-3)
Supplement: Supplementary file 1 — Supplementary file1 (DOCX 26 KB) [file 11686_2024_793_MOESM1_ESM.docx]

**Prophylactic and therapeutic efficacy of ultrasonicated *Rosmarinus officinalis* ethanolic extract and its chitosan-loaded nanoparticles against *Eimeria tenella* infected broiler chickens**

**Shaimaa M. Kasem^a,*^, Nabila M. Mira^a^, Ibrahim B. Helal^b^, Magdy E. Mahfouz^a^**

**^a^** Zoology Department, Faculty of Science, Kafrelsheikh University, Kafr ElSheikh 33516, Egypt.

^b^ Zoology Department, Faculty of Science, Tanta University, EL Gharbia 31527, Egypt.

**^*^Corresponding author:**

Shaimaa M. Kasem, Zoology Department, Faculty of Science, Kafrelsheikh University, Kafr ElSheikh, Egypt. E-mail address: shaimaakasem48@yahoo.com.

**Journal:** Parasitology Research

**Table S1** Fold change of pro-inflammatory cytokines gene expression in the cecum of infected broiler chickens with *E. tenella* of the dietary prophylactic groups with ultrasonicated *Rosmarinus officinalis* ethanolic extract and its chitosan-loaded nanoparticles

| **Groups** | **IFN-γ/GAPDH** | | | **IL-1β/GAPDH** | | | **IL-6/GAPDH** | | |
| --- | --- | --- | --- | --- | --- | --- | --- | --- | --- |
|  | **Day 27**  **(6 DPPI)** | **Day 35**  **(0 DPSI)** | **Day 41**  **(6 DPSI)** | **Day 27**  **(6 DPPI)** | **Day 35**  **(0 DPSI)** | **Day 41**  **(6 DPSI)** | **Day 27**  **(6 DPPI)** | **Day 35**  **(0 DPSI)** | **Day 41**  **(6 DPSI)** |
| **NC** | 1.00±0.00 | 1.00±0.00 | 1.00±0.00 | 1.00±0.00 | 1.00±0.00 | 1.00±0.00 | 1.00±0.00 | 1.00±0.00 | 1.00±0.00 |
| **Positive control** | 1.65±0.01^•^ | 0.62±0.02^•^ | 0.73±0.03^•^ | 1.10±0.02^•^ | 0.91±0.03^•^ | 0.66±0.02^•^ | 1.32±0.00^•^ | 0.68±0.02^•^ | 0.88±0.04^•^ |
| **S-UROEE** | 0.18±0.01^•^ | 0.55±0.02^•^ | 0.40±0.02^•^ | 0.90±0.02^•^ | 1.01±0.05 | 0.21±0.01^•^ | 0.38±0.01^•^ | 0.57±0.02^•^ | 0.15±0.00^•^ |
| **S-CsNPs** | 0.45±0.01^•^ | 0.53±0.02^•^ | 0.37±0.03^•^ | 0.81±0.03^•^ | 1.02±0.02 | 0.20±0.03^•^ | 0.77±0.01^•^ | 0.73±0.03^•^ | 0.29±0.04^•^ |
| **S-UROEE-CsNPs** | 0.36±0.00^•^ | 0.45±0.01^•^ | 0.36±0.01^•^ | 0.65±0.01^•^ | 0.96±0.03 | 0.16±0.01^•^ | 0.88±0.01^•^ | 0.29±0.01^•^ | 0.41±0.02^•^ |
| **P-UROEE** | 1.48±0.04^•*^ | 0.42±0.03^*^ | 0.33±0.01^•*^ | 1.04±0.02^*^ | 0.75±0.03^•*^ | 0.35±0.01^•*^ | 0.69±0.00^•*^ | 0.50±0.01^•*^ | 0.63±0.01^•*^ |
| **P-CsNPs** | 1.33±0.06^•*^ | 0.28±0.01^•*^ | 0.24±0.01^•*^ | 0.88±0.02^•*^ | 0.54±0.02^•*^ | 0.30±0.01^•*^ | 0.57±0.01^•*^ | 0.33±0.01^•*^ | 0.63±0.01^•*^ |
| **P-UROEE-CsNPs** | 1.44±0.02^•*^ | 0.22±0.00^•*^ | 0.13±0.00^•*^ | 0.67±0.03^•*^ | 0.35±0.01^•*^ | 0.23±0.01^•*^ | 0.70±0.01^•*^ | 0.51±0.01^•*^ | 0.78±0.02^•*^ |

NC; negative control group, S-UROEE; supplemented group with ultrasonicated *Rosmarinus officinalis* ethanolic extract at 100 mg/kg diet, S-CsNPs; supplemented group with chitosan nanoparticles at 20 mg/kg diet, S-UROEE-CsNPs; supplemented group with ultrasonicated *Rosmarinus officinalis* ethanolic extract- chitosan loaded nanoparticles at 20 mg/kg diet, P-UROEE; dietary prophylactic group with ultrasonicated *Rosmarinus officinalis* ethanolic extract at 100 mg/kg diet, P-CsNPs; dietary prophylactic group with chitosan nanoparticles at 20 mg/kg diet, P-UROEE-CsNPs; dietary prophylactic group with ultrasonicated *Rosmarinus officinalis* ethanolic extract-chitosan loaded nanoparticles at 20 mg/kg diet. Data are means ± standard deviation. ^•^ Significant (P < 0.05), when compared to negative control group. ^*^ Significant (P < 0.05), when compared to positive control group.

**Table S2** Fold change of pro-inflammatory cytokines gene expression in the cecum of infected broiler chickens with *E. tenella* of the therapeutic treatment groups with ultrasonicated *Rosmarinus officinalis* ethanolic extract and its chitosan-loaded nanoparticles

| **Groups** | **IFN-γ/GAPDH** | | | **IL-1β/GAPDH** | | | **IL-6/GAPDH** | | |
| --- | --- | --- | --- | --- | --- | --- | --- | --- | --- |
|  | **Day 31**  **(6 DPT)** | **Day 35**  **(0 DPSI)** | **Day 41**  **(6 DPSI)** | **Day 31**  **(6 DPT)** | **Day 35**  **(0 DPSI)** | **Day 41**  **(6 DPSI)** | **Day 31**  **(6 DPT)** | **Day 35**  **(0 DPSI)** | **Day 41**  **(6 DPSI)** |
| **NC** | 1.00±0.00 | 1.00±0.00 | 1.00±0.00 | 1.00±0.00 | 1.00±0.00 | 1.00±0.00 | 1.00±0.00 | 1.00±0.00 | 1.00±0.00 |
| **Positive control** | 4.05±0.08^•^ | 0.62±0.02^•^ | 0.73±0.03^•^ | 1.73±0.03^•^ | 0.91±0.03^•^ | 0.66±0.02^•^ | 1.51±0.01^•^ | 0.68±0.02^•^ | 0.88±0.04^•^ |
| **T-UROEE** | 5.60±0.20^•*^ | 0.25±0.01^•*^ | 0.47±0.02^•*^ | 4.00±0.20^•*^ | 0.45±0.05^•*^ | 0.39±0.01^•*^ | 2.96±0.03^•*^ | 0.38±0.17^•*^ | 0.77±0.06^•*^ |
| **T-CsNPs** | 1.07±0.05^*^ | 0.35±0.02^•*^ | 0.19±0.01^•*^ | 2.34±0.05^•*^ | 0.48±0.02^•*^ | 0.13±0.00^•*^ | 1.35±0.02^•*^ | 0.54±0.02^•*^ | 0.18±0.01^•*^ |
| **T-UROEE-CsNPs** | 1.95±0.08^•*^ | 0.54±0.03^•*^ | 0.52±0.01^•*^ | 3.00±0.16^•*^ | 0.47±0.02^•*^ | 0.58±0.03^•*^ | 1.39±0.02^•*^ | 0.15±0.01^•*^ | 0.77±0.06^•*^ |

NC; negative control group, T-UROEE; therapeutic treatment group with ultrasonicated *Rosmarinus officinalis* ethanolic extract at 100 mg/kg B.W., T-CsNPs; therapeutic treatment group with chitosan nanoparticles at 20 mg/kg B.W., T-UROEE-CsNPs; therapeutic treatment group with ultrasonicated *Rosmarinus officinalis* ethanolic extract-chitosan loaded nanoparticles at 20 mg/kg B.W., Data are means ± standard deviation. ^•^Significant (P < 0.05), when compared to negative control group. ^*^Significant (P < 0.05), when compared to positive control group.

**Table S3** Fold change of chicken anti-inflammatory cytokines gene expression in the cecum of infected broiler chickens with *E. tenella* of the dietary prophylactic groups with ultrasonicated *Rosmarinus officinalis* ethanolic extract and its chitosan-loaded nanoparticles

| **Groups** | **IL-10/GAPDH** | | | **TGF-β4/GAPDH** | | |
| --- | --- | --- | --- | --- | --- | --- |
|  | **Day 27**  **(6 DPPI)** | **Day 35**  **(0 DPSI)** | **Day 41**  **(6 DPSI)** | **Day 27**  **(6 DPPI)** | **Day 35**  **(0 DPSI)** | **Day 41**  **(6 DPSI)** |
| **NC** | 1.00±0.00 | 1.00±0.00 | 1.00±0.00 | 1.00±0.00 | 1.00±0.00 | 1.00±0.00 |
| **Positive control** | 0.53±0.00^•^ | 0.24±0.01^•^ | 0.46±0.02^•^ | 2.04±0.06^•^ | 0.78±0.02^•^ | 0.54±0.01^•^ |
| **S-UROEE** | 0.58±0.01^•^ | 0.99±0.02 | 0.26±0.01^•^ | 1.19±0.04^•^ | 0.56±0.01^•^ | 0.15±0.01^•^ |
| **S-CsNPs** | 0.48±0.11^•^ | 1.02±0.02 | 0.10±0.02^•^ | 1.61±0.05^•^ | 0.61±0.01^•^ | 0.21±0.03^•^ |
| **S-UROEE-CsNPs** | 0.33±0.01^•^ | 0.49±0.03^•^ | 0.10±0.00^•^ | 1.59±0.07^•^ | 0.73±0.00^•^ | 0.12±0.00^•^ |
| **P-UROEE** | 0.35±0.01^•*^ | 0.96±0.05^*^ | 0.22±0.01^•*^ | 1.38±0.07^•*^ | 0.66±0.01^•*^ | 0.21±0.01^•*^ |
| **P-CsNPs** | 0.24±0.01^•*^ | 0.88±0.06^•*^ | 0.28±0.01^•*^ | 1.72±0.14^•*^ | 0.40±0.01^•*^ | 0.19±0.00^•*^ |
| **P-UROEE-CsNPs** | 0.21±0.00^•*^ | 0.48±0.02^•*^ | 0.23±0.01^•*^ | 1.43±0.06^•*^ | 0.37±0.01^•*^ | 0.14±0.01^•*^ |

NC; negative control group, S-UROEE; supplemented group with ultrasonicated *Rosmarinus officinalis* ethanolic extract at 100 mg/kg diet, S-CsNPs; supplemented group with chitosan nanoparticles at 20 mg/kg diet, S-UROEE-CsNPs; supplemented group with ultrasonicated *Rosmarinus officinalis* ethanolic extract-chitosan loaded nanoparticles at 20 mg/kg diet, P-UROEE; dietary prophylactic group with ultrasonicated *Rosmarinus officinalis* ethanolic extract at 100 mg/kg diet, P-CsNPs; dietary prophylactic group with chitosan nanoparticles at 20 mg/kg diet, P-UROEE-CsNPs; dietary prophylactic group with ultrasonicated *Rosmarinus officinalis* ethanolic extract-chitosan loaded nanoparticles at 20 mg/kg diet. Data are means ± standard deviation. ^•^ Significant (P < 0.05), when compared to negative control group. ^*^ Significant (P < 0.05), when compared to positive control group.

**Table S4** Fold change of chicken anti-inflammatory cytokines gene expression in the cecum of infected broiler chickens with *E. tenella* of the therapeutic treatment groups with ultrasonicated *Rosmarinus officinalis* ethanolic extract and its chitosan-loaded nanoparticles

| **Groups** | **IL-10/GAPDH** | | | **TGF-β4/GAPDH** | | |
| --- | --- | --- | --- | --- | --- | --- |
|  | **Day 31**  **(6 DPT)** | **Day 35**  **(0 DPSI)** | **Day 41**  **(6 DPSI)** | **Day 31**  **(6 DPT)** | **Day 35**  **(0 DPSI)** | **Day 41**  **(6 DPSI)** |
| **NC** | 1.00±0.00 | 1.00±0.00 | 1.00±0.00 | 1.00±0.00 | 1.00±0.00 | 1.00±0.00 |
| **Positive control** | 0.17±0.01^•^ | 0.24±0.01^•^ | 0.46±0.02^•^ | 1.32±0.08^•^ | 0.78±0.02^•^ | 0.54±0.01^•^ |
| **T-UROEE** | 1.29±0.03^•*^ | 0.63±0.02^•*^ | 0.19±0.01^•*^ | 1.78±0.04^•*^ | 0.58±0.01^•*^ | 0.20±0.01^•*^ |
| **T-CsNPs** | 0.54±0.02^•*^ | 0.73±0.03^•*^ | 0.09±0.00^•*^ | 1.06±0.04^•*^ | 0.67±0.02^•*^ | 0.10±0.00^•*^ |
| **T-UROEE-CsNPs** | 0.25±0.01^•*^ | 0.63±0.02^•*^ | 0.40±0.00^•*^ | 1.12±0.01^•*^ | 0.37±0.01^•*^ | 0.29±0.01^•*^ |

NC; negative control group, T-UROEE; therapeutic treatment group with ultrasonicated *Rosmarinus officinalis* ethanolic extract at 100 mg/kg B.W., T-CsNPs; therapeutic treatment group with chitosan nanoparticles at 20 mg/kg B.W., T-UROEE-CsNPs; therapeutic treatment group with ultrasonicated *Rosmarinus officinalis* ethanolic extract-chitosan loaded nanoparticles at 20 mg/kg B.W., Data are means ± standard deviation. ^•^Significant (P < 0.05), when compared to negative control group. ^*^Significant (P < 0.05), when compared to positive control group.
